# The African Critical Illness Outcomes Study (ACIOS): a point prevalence study of critical illness in 22 nations in Africa

**DOI:** 10.1016/S0140-6736(24)02846-0

**Published:** 2025-03-01

**Authors:** The African Critical Illness Outcomes Study (ACIOS) Investigators, Tim Baker, Juan Scribante, Muhammed Elhadi, Adesoji Ademuyiwa, Babatunde Osinaike, Christian Owoo, Daniel Sottie, Karima Khalid, Adam Hewitt-Smith, Arthur Kwizera, Fitsum Kifle Belachew, Degsew Dersso Mengistu, Yared Boru Firissa, Tirunesh Busha Gemechu, Gaudencia Dausab, Unotjari Kauta, Kaveto Sikuvi, Nahla Kechiche, Kelan Bertille Ki, Martin Mukenga, Dolly Munlemvo, Mustapha Bittaye, Abubacarr Jagne, Mohamed Abdinor Omar, Hassan Ali Daoud, Mohamed Faisal, Mahmoud Elfiky, Mpho Seleke, Tarig Fadalla, Alshaima Koko, Alemayehu G Bedada, Gilles Niengo Outsouta, Marie Elombila, Ahmed Rhassane El Adib, Meryem Essafti, Dino Lopes, Atilio Morais, Pisirai Ndarukwa, Newten Handireketi, Fred Bulamba, Busisiwe Mrara, Hyla-Louise Kluyts, Marian Kinnes, Gillian J Bedwell, Hanel Duvenage, Gwendoline Arendse, Luke Hannon, Landon Myer, Anneli Hardy, Carl Otto Schell, Rupert M Pearse, Bruce M Biccard, Mohammed Adem, Tizeta Belachew, Ermiyas Belay, Yared Boru, Turunesh Busha, Selam Daniel, Abiy Dawit, Brook Demissie, Degsew Dersso Mengistu, Kokeb Desta, Kelem Desta, Desta Galcha, Nuroadis Guchima, Peniel Kenna, Fitsum Kifle, Tewodros Kifleyohanes, Betelehem Mulye, Bezaye Zemdkun, Gwen Arendse, Gillian J Bedwell, Bruce M Biccard, David G Bishop, Marcelle Crowther, Lucy Cunnama, Yamkela Desemela, Hanel Duvenage, Rowan Duys, Robert A Dyer, Margot Flint, Aphelele Futshane, Simphiwe Gumede, Anneli Hardy, Kasandji F Kabambi, Marian Kinnes, Hyla-Louise Kluyts, Edson Mafana, Salome Maswime, Steve Molaoa, Lebogang Moloi, Busisiwe Mrara, Lwandile Mtshabe, Ezile J Ninise, Linda Pohl, Rosalind V Prinsloo, Catherine F Mokotla, Rev. Mzubanzi Mdunyelwa, Tim Baker, Upendo J Bandeke, Happines Biyengo, Aneth C Kaliza, Karima Khalid, Bernard Mbwele, Juma S Kitwara, Elibariki Mkumbo, Linda Mlunde, Rafael S Shayo, Anab F Issa, Alma O Damasy, Godfrey Barabona, Charles Machumu, Anna Hvarfner, Sabra Hussein, Elvis Amunyo, Fred Bulamba, Richard Gamubaka, Adam Hewitt-Smith, Assen Kamwesigwe, Muzamiru Kawiso, Justine Khanyalano, James Kidulu, Peter Magala, Joanitah Nakibuule, Joan Naluyima, Hasifah Namutebi, Rose Nandutu, Juliana Nanimambi, David Okanya, John Steven Walutsyo, Herbert Kiwalya, Aneel Bhangu, Justine Davies, Priyanthi Dias, Ryan Ellis, Charles Fadipe, Alexander J Fowler, Thomas Hamborg, Borislava Mihaylova, Jolene Moore, Rupert M Pearse, Timothy J Stephens, Nicola J Vickery, Cecilia Vindrola, Shingirai Muzondiwa, Kagiso Wadikonyana, Tariro Mubika, Wabotlhe Ntwayapelo, Abdramane Ouattara, Papougnezambo Bonkoungou, Martin Lankoundé, Nathanael Bamogo, Bayé Nébié, Salam Savadogo, Ismael Guibla, Daouda Barro, Latifa Aida Sawadogo, Guy Thierry Ki, Bego-wena Bado, Ibrahim Alain Traore, Salah Traore, Wendelamita Kaboré, Salifou Napon, Arouna Louré, Marame Diop, Marie Elombila, Otiobanda Gilbert Fabrice, Niengo Outsouta Gilles, Mpoy Emy Monkessa Christ Mayick, Iwoba Rebet Sarrah Armanda, Koumou Banga Fernand Régis, Jean Jacques Kalongo, Patrick Mukuna, Frank Nguvulu, Berthe Barhayiga, Patrick Mukuna, Martin Mukenga, Jeremie Kalambayi, Ted Likongo, Sarah Magdy Abdelmohsen, Mohie El-Din Mostafa Madany, Ahmed Saber Abdelrahman, Sarah Mansour, Ayalew Zewdie Tadesse, Daniel Adugna Dashura, Alemayehu Beharu, Seble Ashagre Awoke, Tsegay Gebreanenia Hagos, Eyuel Teshome, Amare Yohans Mekonen, Abnet Ewnetie Emirie, Desta Galcha Gerbu, Weldemichael Yigebahal Belay, Aregahegn Mulgeta Munka, Begashaw Melesse Dicha, Addisu Tesfaye Koster, Moges Tessema Hesbeto, Zerihun Tesfaye, Wondimu Dori, Roba Kebede, Mihret Kaleb, Selamawit Yizelkal, Abraham T Markos, Melaku Getachew, Fanta Wondimneh, Tilahun Teshager, Gamachu Bekuma, Emnet Tesfaye Shimber, Kindie Nigatu Woubshet, Kibru Kifle Karicha, Melaku Teshale, Gemechis Melkamu Fufa, Addis Ketemaoboro, Mastewal Yiseni Yimer, Tilahun Mebre Defersha, Tewodros Assefa Gossa, Dereje Zewdu, Tilahun Temesgen, Khedir Fikadu, Shimelis Getu, Sime Assefa, Feleke Habte, Dado Jabbie, Marie Saal Mendy, Isatou Bobo Jallow, Fanta Fofana, Lamin FTT Fadera, Bubacarr Jabbie, Karimadi Fofana, Divine Nko-Onye Moseri, Sheikh Omar Bittaye, Fatou Jatta, Anna Donkor, Awa Mboob, Abubacarr Jagne, Fatou Jallow, Kajali Camara, Majula Ceesay, Lamin Sanyang, Kawsu Fatty, Muhammed Touray, Lamin O Beyai, Ebrima A Jallow, Modou Lamin Conteh, Bubacarr Sowe, Roheyatou Jawara, Ndumbeh Manneh, Oluwayemisi Esther Ekor, Emmanuel Owusu Ofori, Patience Koggoh, Luke Adagrah Aniakwo, Evans Kofi Agbeno, Ganiyu Adebisi Rahman, Kwasi Agyen-Mensah, Thomas Agyen, Martin Morna, Michael Nortey, Makafui Yigah, Ansumana Bockarie, Temabore Victoria Daboner, Yvonne Ayerki Nartey, Abigail Serwaa Boateng, Prince Arthur, Quainoo Arnold Kofi, Papa Kojo Mbroh, Edwina Okaikai Obodai, John Benjamin Annan, Kwaku Nti Acquah, Kwadwo Amoah, Gloria Ababio, David Sackey, Emmanuel Tagoe, Jonathan Kinphul, Arko Akoto Ampaw, Foster Amponsah-Manu, Philip Lartey, Gabriel Attipoe-Djagmah, Ijeoma Aja, Francis Wuobar, Audrey Addy, Kwesi Edwin-Tenkorang, Ransford Aduah, Denutsui Etornam Rhoda, Ekuamoah Violet, Esther Adjei-Acquah, Agnes Avorwulanu, Ruth Dzifa Ntumi, Charles Edem Tsivanyo, Tetteh A Kwame, Pinto Henry Kabukie, Andrea Asante, Kingsley Damilare Adeoye, David Danquah Antwi-Agyei, David Abass, Mercy Asare, Kwame Diaw, Isaac Asenso Brobbey, Rose Mensah, Dominic Awuah, Afia Kyere, Ebenezer Oduro-Mensah, Roxana Segborwotso, Marion Amo-Mensah, Reuben Grumah, Alberta Twumasi-Boakye, Aubrey Tigwii, Selywn Agyemang, Mercy Akoto, Agnes Amonoo-Mends, Martha Dsane-Lamptey, Ambe Obbeng, Henrietta Nana Serwaa Fiscian, Simeon Biney, Dorcas Osei-Poku, Nana Ama Christian, Frederickson Pobee, Dorinda Norbetha Lee, Magdalene Boamah, Ayishetu Ndeogo, Mamm-Mavies Tsotsoo Tetteh, Adu-Brempong Carl, Zelda Robertson, Linda Addai, Gideon Appiah-Boadu, Al-Hassan Dasana Andani, Salamatu Nantongma, Joseph Oliver-Commey, Lawrence Ofori-Boadu, Irene Bandoh, Akwasi Antwi-Kusi, Moses Siaw-Frimpong, Eunice Aboagye, Sophia Naa Ayikailey Ankrah, Stephen Opoku Ofori, Pearl Nyarko Korang, Samuel Nana Prempeh Agyeman-Gyebi, John Adabie Appiah, Yaw Ampem Amoako, Felix Owusu Osae, Stephen Sarfo, Kwaku Gyasi Danso, Oppong Philip Peprah, Martha Poku, Maame Temah Appiah-Berko, Ebenezer Akomea Agyin, Anita Eseenam Agbeko, Paa Ekow Hoyte-Williams, Emmanuel Osei Kankam, Joseph Bonney, Shiela Afua Kedze, Dennis Dwomoh Nkrumah, Eva Adu-Boakye, Yaw Larbi, Kwabena Addow Opare-Addo, Amanda Badjo Amankwata, Christian Owoo, Kwadwo Opoku-Darko, Daniel A Sottie, Pokua Sarpong, Grace-Imelda Obeng-Adjei, Lorraine Baffour-Awuah, Ernest Aniteye, Desmond Seshie, Simitsewa Amoo-Aidoo, Adjoa Ofei, Supriya Wassiamal, Anita Ohenewa Yawson, Nancy Asiedua Larbi, Kwame Afriyie Gyamera, Kelvin Asamoah-Agyepong, Kwaku Obeng, Naa Martekuor Vanderpuye, Cornelia Quarcoopome, Khadijah Bandawu, Aisha Sumaila, Nuhaila Alhassan, Lydia Apraku-Peprah, Leslie I Adam-Zakariah, Kwadwo Darko, Bless-Michael Bonney, Raphaela Agyarko, Felicia Birch Freeman, Henry Kwesi Bulley, Nana Serwaa Agyeman Quao, Steve Blankson, Aba Yorke, Priscilla Allotey, Theophyllus Addo, Gifty Addo, Theodore Boafor, Maryanne Zuolo, Kofi Adzi Gudugbe, Henry Kumi, Ken Atobrah-Apraku, Eric Tetteh, Efua Thompson, Jerry Coleman, William Klah, Michael Ntumy, Kwaku Asah-Opoku, Gordon Amoh, Nicholas Siklere, Precious Owoo, Eunice Nyankah, Faisal Adjei, Elvis Ohemeng-Mensah, Jefferson Owusu-Adae, Prince Larvie, Clarence Abeareba Basogloyele, Latif Saiba, Dela Dinku, Ernest Yorke, Yaw Ofori-Adjei, Jane Afriyie-Mensah, Fiifi Duodu, Beatrice Baaye, Woedem Tettey, Akosua Agyen Frimpong, Vishnu Abayateye, Kwame Darko, Michael Kwapong-Nyarko, Solomon Atindama, Joachim Amoako, Kenneth Baidoo, Estella Bilson-Amoah, Eugene Owusu-Achaw, Evans Sefa Asiedu, Daasebre Ahensan, Kow Entua Mensah, Innocent Adzamli, Charles Kofi Amoah, Desrie Gyan, Matthew Owusu Boamah, Paa Kwesi Blankson, Prudence Nutsuklo, Daniel Baddoo, Isaac Asiedu, Dela Fiagbe, Esinu Akosua Agbeli, Kingsley Abankwa, Patrick Bankah, Omane Acheamfour Okrah, James Evans Mensah, George Darko Brown, Doreen Anderson, Elikem Ametepe, Geoffrey Birikorang, Priscilla Vandyke, George Nketiah, Alfred Seedah, Adejoke Aiyenigba, Barbara Boi, Serwah Amoah, Comfort Gaituah, Edith Ntumy, Imoro Braimah Zeiba, Rachel Kumi Adamson, Nana Yaa Asuama Afful, Emily Martha Nortey, Freda Amoateng, Senyo Fumador, Esther Brobbey, Samuel Antwi Oppong, Priscilla Agyemang-Duah, Alfred Edwin Yawson, Kwame Ofori Boadu, Rita Larsen-Reindorf, Emmanuel Sam Kwabena Baffoe, Fredrick Gyamfi Apraku, Nana Takyiaw Borki Owusu, Margie Kyei, Yaw Akyina, Alex Agbanu, Joshua Dennis Krah, Osei Amponsah-Kwatiah, Kwame Danso, Kamarudeen Korku Hussein, Appiah Minka, Rosemond Boah, Louis Osei, Hajara Alhassan, Sarah Dwomoh, Ernestina Sarfo, Rita Amponsah, Prosper Kwaku Gbekor, Laila Adutwum, Yaa Bema, Mabel Osei-Tutu, Eric Kwame Detoh, Seth Obiri, Michael Gyamfi, Paul Afriyie, Seth Asante Egyin, Randolph Baah Adu, Victor Vandel Adjadeh, Albert Amissah Asiedu, Julius Abuku, Joycelyn Maame Esi Darkwah, Richard Dankwah, Edmund Oko Codjoe, Dorcas Rockson, Bernard Nyamekye Antwi, Gifty Obo-Ninsin Baidoo, George Kwame Prah, Petrina Bingab, Emmanuel Koomson, Emmanuel Ghartey, Frederick Duah Yao, Panyin Benyimah Avemee, Anyemedu Asare Fredovich, Isaac Korku Akotoye, Josephine Okine, Agnes A Anane, Akosua Owusu Sarpong, Marion Okoh-Owusu, Amoako Duah, Kwame Ekremet, Nana Akosua Oppong-Nkrumah, Joseph Tanlongo, Dela Andrea Nutsugah, Kordai Mould, Susan Siabi, Kwame Anim-Boamah, Mohamed Fathi Khmera, Aya Rasem Khmir, Abdulrahman Mohammed Alghziwi, Khayri Karban, Areej Dakshi, Mohamed Saleh Addalla, Taha Khaled Elfaituri, Taha Alkabat, Ahmed Muhsin Alatiweel, Mohammed Salaheddin Dabaie, Abdulhamid Shaban, Ali Abdulnasir Kredan, Abdulalim Ramadan Kuridan, Abdurrahman Abdussalam Haddud, Ahmed Eldeeb, Ahmed Jreibi, Nafati Taher Alnafati, Nabeel Ateeyah Faraj Atiyah, Mohamed Naser Lawgali, Aml Emran Khalleefah Alwirfili, Mustafa Khalil Abdallah Elrgeig, Rawan Othman, Fatma Ali IKday, Marwa I.M Shoukrie, Msara Jamal Haider, Majdi Ehmeda S Kamil, Ameerah Ali Hassan Rahoumah, Kamla Ali, Honayda Almuakkif, Shahed Alaref, Aya Haddad, Amal Nasser, Maram Abdulgani, Alsnosy Abdullah Khalefa Mohammed, Osama Alemenefie, Ahlaam M Ali Ayad, Ahmed Elfaituri, Anwar Salah Hussain Mohamed, Reem Abdullah Salim Alkikli, Alaedden Abdalla Akhmag, Saleh Abdulla Hadia, Salim Almaqtouf, Mohamed Alsori Alharari, Ali Alsouri Alharari, Rawia Adel Mohamed Draa, Abdulmunem Mustafa Olu, Marwa Almabrouk Alazomi, Randah Dhu Omar Aldeeb, Arwi Kara, Ayoub Akwaisah, Mohamed Al Gharyani, Mostafa El Awami, Aya Essa, Abtisam Alharam, Aeshah Aboukaleesh, Fatimah Aboukaleesh, Noura Kareem, Narjis Husayn, Mohamed Adel, Fatimah Mohammed Alenani, Iesra Eldagheili, Abdul-muhaymin Almuquryaf, Rabab Alkurghali, Sara Alsaeiti, Hadeil Abd Elaziz, Salma Akhlaif, Fawzia Alferjani, Wijdan Sayfulnasr, Dalal Salah, Muna Denini, Ahmed Ahmayda, Saja Almugla, Salwa Ali Awidat, Abdulsalam Albadri, Fatima Elfeituri, Arwi Adrees, Mohammed Abosedra, Ameerah Abraheem, Fathiah Elferjani, Malak Barghathi, Yasmin Elmgawob, Amani Zoubi, Nourfan Altarhouni, Asia Bolifa, Malak Areibe, Ehab Othman, Suhaib Issa, Tasnim Hasan, Salem Senussi, Marwah Aleidhah, Fadelalla Elmozoghi, Nisren Alsalme, Fatma Elashhab, Almotasem Bellah Elsharif, Abdulmuez Abdulmalik, Rana Shembesh, Eman Bureziza, Sara Abdelmaged, Hana Faraj, Najla Alaguri, Mohammed Salem, Mohamed Alamrony, Mustafa Amraja, Mohammed Elferjani, Mohammed Alabeedi, Mohammed Yahmad, Mubarka Alzarouq, Hamida El Magrahi, Abir Ben Ashur, Salem Ali, Hibah Bileid Bakeer, Akram Alkaseek, Haitam Shames, Aya Alqaarh, Hashim Aborkhis, Widad Nouralddeen Faraj Amhimmid, Hala Misbah Ali, Taha Husayn Alhadi Alfeeras, Sundes Daba, Ahmed Abdurrahman Algeblawi, Abdulwahab Alzeldeen Abdalei, Fatima Asedeq Abdulali, Ahmed Ismael Saleh, Abdulhamid Mohamed Alailesh, Mohamed Moftah Assalhi, Mohanad Taher Bintaher, Boshra Basher Hashim, Weam Mohammed Drah, Abdelaziz Mahjub Gobbi, Eman Mohammed Abdalhafit Alabani, Hamdan Bashir Hilan, Omar Mohamed Ertaiba, Ahmed Muftah Taweel, Wesal Tarik Yahya Hamad, Hadia Mohamed Omar Aldilfaq, Aya Adam Hassan Mousa, Abdurraouf Abusalama, Kusay Ayad, Abdurrahim Elzoubi, Mohammed Altarabulsi, Alabas Almigheerbi, Ahmad Alfayad, Hatim Elgbaili, Abdulmuhaymen Elmeshrgie, Mohammed Albashri, Mouna Ahmed A Abuhshaima, Khadeja Mohammed Mohammed Alawal, Essraa Ali Alhudhiry, Anwaar Ali Othman Eshnaf, Safia Adem Abdulla, Firdous Assadeq Mllie, Mona Abdu Alsalam, Etidal Ali Abuanniran, Bushra Oezo, Sara Egreara, Nabila Abdalkader, Huda Abdelmajed, Hajer Abdulhamid, Ghazala Abouklaish, Ahmed Alajeeli, Khawla Alalage, Ibtehaj Albaewi, Malak Albusayifi, Azhaar Aldeeb, Ali Algarradhi, Najwa Alkowash, Safa Almaakef, Mohammed Almahjoubi, Hana Altam, Masarah Ateeyah, Sahar Azzaz, Hajir Bahroun, Doha Blaou, Safa Barayik, Bilqays Habhab, Mohamed Hassan, Saja Khalifa, Aya Maiw, Yasmeen Meelad, Safiya Mohamed, Ali Omar, Ali Tirihbat, Saifaleslam Elsahli, Abdulwahhab Fakroun, Manal A. Mohammed Zaglam, Areej Alhassan Abdullah Benghuzi, Shaymaa Moftah Salem, Sari Sulayman Abdulhafith, Hana Adrees Hasan, Najwa Ibrahim, Mohammed M. M Abdaljalil, Eman Moftah Faraj, Amani Mousay Abdulmawlay Mohammed, Malak Mashery, Eman Ahmed Moftah, Halima Soliman Abdualnabi, Eman Gaith Ahwaia, Fatama Mohamed Salem Selaman, Esra Abdulaziz Hamad Albarani, Dua Aljali, Aminah Eid Abd Alsameea, Morad G Rahel, Murajia Mahmoud, Aisha Mabrouk, Marzouga Mabrouk, Rehab Rejab, Ayyah Emran, Anas Mohammed Aboutartour, Batool Ahmed Abdulkarim, Buthuynah Mohammed Alsaeh Alqahwash, Laila Ramadan Askar, Nada Ali Omran Dhem, Amer Mohamed Abdulaziz Mosbah, Khalid Asad Bin Qanad, Abdurraouf Musbah Khalifa Said, Qabs Madi, Amal Ibrahim Furjane Khalifa, Mohamed Hamed Said, Abdulrhim Omar Mohammed Almeshri, Marwa Muftah Daloub, Yara Talal Mohammed Bariun, Ahmed Nureddin Ben Shaban, Dareen Ahmed Elmahdi, Dawoud Amhimmid Saeid, Firas Nureddin Hammas, Mahad Mahmoud Qaeim, Feras Mohamed Shneib, Nouri Khalefah Afheej, Muad Fathi Abuhallalah, Abdalla Mustafa Abdalla Hdidan, Abdulmalik Ibrikat, Aisha Abdulfatah Gehimi, Aisha Alwarfally, Randah Dhu Omar Aldeeb, Mawada Mohammed Elgeriane, Rabeeah Abd Aldaem AbuAlneeran, Abdulhamid Mohammed Alsagheer, Safa Zaydan Ammar, Arwa Tawfeeq Abdulnabi, Bassam Erhoma, Mohamed Mustafa Elghazal, Mohamed Abdusalam Elhaderi, Mohammmed Yousef Elimselati, Esra Abdulhafed Abuaen, Esra Ben Zahra, Abduladim Omran Ezaddin, Hidaya Badri Dozan, Wadad Mohammed Jomaa, Lobna Shawesh, Mosab Emhemmed Matous, Ghaida Abdalla Naana, Nada Abdulmonam Elhoush, Ola Abdulqadir Alsharif, Rawia Adel Mohamed Draa, Ritaj Mahmoud Ghasem Agha, Reyam Abdalla Naana, Abdulsabur Mohamed Salih, Sanabil Mansour Abdullah, Serag Lameen Almzainy, Tasneem Musbah Fara, Najat Ben Hasan, Abdulmalik Abeed, Hajer Abusnina, Majdolin Miloud Almahjoub, Radhwan Alsaedi, Lubnah Alsunousi Mohammed Alokshi, Manal Almaqrahi, Hudi Dalaf, Fahed Gareb, Sehar Naji Ashini, Mwada Tarek Mehdi, Malefane Motsama, Mpata Seleso, Mpine Maqolo, Maphiri Ramafikeng, Tina Ntee Ntsane, Thato Molahlehi, Amohelang Moreki, Lerato Mohlalisi, Molahlehi Makhalanyane, Noi Khoeli, Lebohang Lebina, Samia Errami, Soulaimane Laaziri, Ayoub Bousselham, Iltimass Gouazar, Mohamed-Sami Melouane, Hind Essalim, Rim Belarbi, Rihab Belfouzy, Hasna Laghmami, Oumama Boujidi, Salma Amahmid, Riyad Ghailan, Said Errahouy, Adam Mohamed Aajly, Oussama Ennamra, Nouha Smily, Raouia Fares, Abdellah Agnaou, Dino Mariano Lopes, Elka José Nhaduco, Francisco Jaime Gonçalves Taimo, Nadia Gertrudes Miséria Joaquim Estafeira, Onésia Lucia Sérgio Chitsembe Mombassa, Sebastião Moisés, Mouzinho Saide, Cesaltina Lorenzoni, Luis Gonçalves Ferrão, Gaudencia Dausab, Nasjtasha Pieterse, Anna Hangula, Fhenny Moongo, Natasha Nghitukwa, Rejoice Makongwa, Dhruvin Das, Njohela Mwandemele, Christian Ndambi, Larisa Jafta, Hilma Uugwanga, Maria Sibolile, Seuna Karuaihe, Bitoma Thotho Amisi, Munikasu Christopher, Sinvula Lutombi, Masongo Annie, Matomola Chuma, Chipman Kamilla, Nzwile Alphoniso, Sisamu Mwiza Daphine, Kawana Concilia, Estha Masasa, Lilungwe Sonnety, Njahi Kaiba, Evans Sanga, Rebecka Martin, Hilma Namuhuya, Vute Nekwaya, Loide Johannes, Goodman Uushona, Daylight Manyere, Elina Muulu, Lindah Kabende, Joseph Chinanga, Chloe Paulse, Natasha !Gontes, Hambeleleni Kamati, Frans Nambinga, Loide Namwandi, Nasjtasha Pieterse, Anna Hangula, Fhenny Moongo, Natasha Nghitukwa, Faith Dzenga, Dhruvin Das, Njohela Mwandemele, Christian Ndambi, Ndapunikwa Nghihalwa, Justina Shikongo, Tracy Mweti, Larisa Jafta, Hilma Uugwanga, Melissa Spiegel, Maria Sibolile, Seuna Karuaihe, Ibrahim Salim Abdullahi, Auwal Adamu, Rabiu Mohammed Bashir, Abubakar Muhammad Ballah, Isa Bashiru Ibrahim, Yusuf Raiyanu Jasawa, Aliyu Bashir Adamu, Saidu Yusuf Yakubu, Bilkisu Adamu, Oluseyi Oyebode Ogunsua, Shafaatu Ismail Sada, Tunde Talib Sholadoye, Alfa Yakubu, Halima Olufunmilola Abdulsalam, Fomete Benjamin, Samuel Isa Gana, Abdulkadir Muhammad Kabiru, Hamisu Yakubu, Rabiu Isah Mohammed, Sufyan Ibrahim, Umma Suleiman Bawa, Ganiyat Ronke Olagunju, Babangida Salahu Mohammed, Mohammad EL-Amin Idris, Michael Odigbo, Fatima Mahmud-Ajeigbe, Aghadi Ifeanyi Kene, Tasiu Saadu, Sheidu Owuda Abdullahi, Rabi'at Muhammad Aliyu, Mudi Awaisu, Chitumu Dotiro, Stephen G Gana, Lucy E Okwajebi, Muhammad Daniyan, Abdulghaffar Adeniyi Yunus, Olutayo A Gana, Oguntayo Olanrewaju Adekunle, Muhammad Lawal Abubakar, Ibrahim Ibrahim Lawal, Atiku Likunga Atiku, Ukwubile Linus, Ahmad Bello, Ahmad Tijjani Lawal, Aminu Muhammad Balarabe, Anisah Yahya, Shehu Toro Muhammad, Emmanuel Raphael Abah, Fakuta Tathiya Naiwa, Hussaini Yusuf Maitama, Nwoye Ugo Daniel, Elizabeth Ogboli-Nwasor, Abdullahi Sudi, Muhammad Raji Mahmud, Ugwu Euphemia Mgbosoro, Igele Agom Cletus, Abass Oluwaseyi Ajayi, Emmanuel Paktama Bwala, George Duke Mukoro, Habibu Balarabe, Sani A Abubakar, Bilqis O Muhammad, Ademola O Adeleye, Sunday O Ajike, Donatus O Egwu, Richard L Ewah, Promise O Ubanatu, Cletus U Onwe, Nathan O Mbawike, Joshua A Adebayo, Chijioke Udu, Nneka N Chiege, Stephen O Nwafor, Stephen C Eke, Pearl C Eke, Chinyere S Onoka, Juliana Chi-Nwogo, Mary Okoyari, Oluchi F Ogah, Chinenye T Agbo, Obiageli A Obi, Mercy N Nwangwu, Loveth N Ugochukwu, Kelechi Adindu, Stella A Ugochukwu, Obiora C Bernard, Sunday V Nwali, Omotayo Felicia Salami, Clifford Imonitie, Usman Kolawole Ajayi, Ayodeji Emmanuel Babalola, Abiodun Idowu Okunlola, Cecilia Kehinde Okunlola, Segun Alex Atolani, Olumuyiwa Ariyo, Tesleem Olayinka Orewole, Olakunle Fatai Babalola, Adedayo Idris Alawu, Paul Olukayode Abiola, Omagbeitse Henry Abiyere, Augustine Adebayo Adeniyi, Adewumi Bakare, Ajayi Adeleke Ibijola, Ibrahim Salisu, Auwal Mohammed Abdullahi, Naziru Garba Shuaibu, Ibrahim O Shuaibu, Akeem Ibiyemi, Jafar Halliru, Sanusi Bala, Sani Kasim Inuwa, Gamaraddeen Abdullahi Muhammed, Auwal Haruna, Amina Umar Akeel, Yahaya Lawal Kankara, Irene Irenosen Akhideno, Kelvin Salami, Peter Itua, Patrick Iniaghe, Rachael Izekor, Christian Uanzekin, Ehizojie Fidelis, Samuel Isaiah Nuhu, Henry Yammoh Embu, Mangai Audu Ngeh, Kefas Thomas Malau, Musa Abdullahi Aliyu, Husseina Amina Aliyu, Udoka Okorie, Oluwaseun David Oladokun, Obashina Ayodele Ogunbiyi, Oluwayemisi Bamidele Oluwadun, Oluwafunmilayo Aderemi Ikotun, Akintayo Olugbemi Ogunjuboun, Yetunde Adebimpe Oyeyode, Nasiru Akobe Suleiman, Folayinka Ayofunke Ogunmuyiwa, Ilochi Uchechukwu Nnaji, Ibrahim Olajide Dada, Fatima Ajuma Ojabo, Muyiwa Rotimi, Adesoji Ademuyiwa, Damilola Awotunde, Chisom Agwu, Oluwajuwon Afolayan, Jennifer Okei, Abidat Ashimi, Oluwadarasimi Adeboyeku, Abimbola Ogunbadejo, Daniel Lawal, Shakirat Adejumo, Goodness Donye, Tobiloba Lawal, Gideon Osadare, Titilola Awosika, Jared Oseghale, Danielle Obiwulu, Christianah Otegbola, Emmanuel Williams, Maduabuchi Paul Ufoegbunam, Chika A Ndubuisi, Friday G Okonna, Obioma Richards Akwada, Donald E Ogolo, Fatungase Oluwabunmi Motunrayo, Shoyemi Ramotalai Oluwatoyin, Shotayo Oluwakemi Adenike, Adefuye Bolanle Olufunlola, Ogunjimi Luqman Opeoluwa, Nwokoro Chigbundu Collins, Ogundele Ibukunolu Olufemi, Amosun Lukmon, Olusola Kayode Idowu, Babatunde B Osinaike, Afolabi Adebayo Oladeji, Oluwaseun Kehinde Adebayo, Mutiu A Jimoh, David A Aderinto, Yemi Raheem Raji, I Adeola Fowotade, Ajibola Oladiran, Oluwakemi Badejo, Kehinde Abraham Ojifinni, Adigun Tonitomi, Mosimabale J Balogun, Taiwo A Lawal, Oyeyemi E Dada, Yewande Olaoye Babalola, Oluwasanmi Adekunle Ajagbe, Olurotimi Olaolu Akinola, Oluseun O Saanu, Olatunji Okikiola Lawal, Olayinka Ramotu Eyelade, Adegbolahan Jacob Fakoya, Olukemi A Adekanmbi, Mukaila O Akinwale, Arinola Adeyoola Sanusi, Foluke Oladele Sarimiye, James Ayokunle Balogun, Matias Ogbonia Orji, Afieharo I Michael, Rukiyat Adeola Abdus-Salam, Dare Isaac Olulana, Omobolaji Oladayo Ayandipo, Oludolapo Olawunmi Afuwape, Sikiru Adekola Adebayo, Tarela Frederick Sarimiye, Oluseyi O. Agboola, Thankgod C Okonkwo, Augustine Oghenewveyin Takure, Adekunle Daniel, Douglas Efe, Akpabio Uforo Ezekiel, Imuetinyan Rashida Edeki, Aluya Eseosa Faith, Ehiorobo Samson Edohen, Omorogiuwa Idemudia Oduware, Aighobahi George Akpede, Peter Ikponmwosa Agbonrofo, Michael Ediale, Joel Enaholo, Otasowie Osagie, Oduware Emmanuel Ehigiegba, Noruwa Patience Ekhator, Omorogbe Scott Osahon, Osaheni Osayomwanbo, Stella A Eguma, Sunday O Sangolade, Chinedu J Anachunam, Nkoyo E Enyenihi, Victoria E Ndoma, Augustine O Odemwingie, Olanrewaju Olubukola Oyedepo, Benjamin Olusomi Bolaji, Christianah Iyabo Oyewopo, Abdulrasheed Adegoke Nasir, Olayide Sulaiman Agodirin, Lookman Oluwatosin Lawal, Anne Oluwabunmi Mokuolu, Audu Idrisa, Ahmed Hamman Gabdo, Nuhu Ali, Jamila Audu Idrisa, Mohammed A Ahmed, Mohammed A.S Abdullahi, Ali Mohammed Ramat, Ibrahim Musa Kida, Babagana Bako, Hassan M Dogo, Babagana Usman, Jibril Khalil, Hamman Ibrahim Garandawa, Sulaiman M Maina, Olayinka Adewunmi, Maryam Usman Kashim, Job Otokwala, Vernatious Aniobi, Busola Alagbe-Briggs, John Udo, Ageh Hannah, Hilal Mohamed Nor, Abdishakor Mohamud Ahmed, Mohamed Abdi Ahmed, Abdullahi Said Hashi, Mohamed Sheikh Hassan, Mohamed Farah Yusuf Mohamud, Nasra Mohamud Hilowle, Marian Muse Osman, Sakariye Abdullahi Hassan, Suleyman Abdullahi Mohamed, Timothy Kimutai, Abdullahi Mohamed Mohamud, Mouna Ahmed Abdillahi, Fatima Eid Ibrahim, Omar Aden Yusuf, Mohamed Said Awil, Hamse Awil Mohamoud, Ayoub Mohamed Suleiman, Mohamoud Abdullahi Ismail, Samira Maxamed Macalin, Abdirahman Abdirisak Ahmed, Sebenzile Sikhakhane, Usha Singh, Jayd Kanjee, Nishen Gokal, Nqobile Zulu, Sabihah Murchie, Mausum Beeput, Nokukhanya Shange, Sarisha Haripersad, Leona Ravinath, Kerissa Naidoo, Thabang Kolanyane, Njabulo Ntuli, Lusanda Magwenyane, Fezile Mkhize, Thubelihle Nsele, Lethuxolo Shange, Angela Mary Thain Hartwig, Relebohile Joyce Khoabane, Mfundo Gubhela, Bongeka Mfecane, Musa Sarile, Thobile Dlalisa, Nashlen Naidoo, Shepherd Nzenza, Jacob Myeni, Kwanele P Majozi, Mlungisi P Shange, Zakhele Nxumalo, Ziphokuhle Khumalo, Mujinga Sylvie Biaya, Siphelele Kubheka, Rodney Magwenya, Thuba Mazibuko, Nkanzimulo Dlamini, Khayelihle Jobe, Mondli Mfeka, Lindokuhle Sangweni, Snegugu Zondi, Mbongeleni Zuma, Siphesihle Njoko, Mounir Raddadi, Lindokuhle Zungu, Judy-Liesel Hardie, Marion Irene Frost, Mandisa Sfundo Ziqubu, Elsje Bester, Anli van Niekerk, Heinri Edwards, Joy Awokiyesi, Sinazo Ngini, Carli Whitehead, Theroshnie Kisten, Halalisiwe Khanyi, Shakeel Kader, Matthew Jones, N Buthelezi, Tessa Korda, Chris Hooper, Urekha Ballasur, Susan Brown, Alicia Ackerman, Ahmad Asmal, Muhammad Asmall, Nico Hudson, Rene Kruger, Kedibone Mbanga, Vuyisa Mdingi, Molifi Moshoadiba, Zanine Moyce, Fezile Mthimkhulu, Akhie Narain, Marsha Ramburuth, Tapiwa Sibanda, Sivenderen Thaver, Cherade Wilson, Nondumiso Zondi, Mkholisi Gama, Genevie Borrageiro, Martha Elizabeth Bronkhorst, Danielle Charlotte Hendricks, Mampho Mochaoa, Yonela Tsewu, Mbuso Nkala, Lwanzo Mathe, Deliwe Nkosi, Roel Matos-Puig, Ernest Muragijeyesu, Ria Devi Naidoo, Nompumelelo Precious Sibiya, Nthabiseng Precious Molokwane, Shilendra Harripersad, Dyavan Singh, Vajra Chandrasekhar, Arisha Ramkillawan, Nomandla Tsibiyane, Nandini Gramoney, Thubelihle Jali, Michelle Terry Dolores Smith, Sinovuyo Madikane, Ayanda Ntinga, Sinenhlanhla Mkhize, Zukiswa Cabangana, Sakhisosenkosi Zamimpilo Hlela, Parvania Munthree, Takudzwa Shava, Lieze Geldenhuys, Dilshad Shaik, Jithin Mohan, Mihir Patel, Sabeeha Khan, Fathima-Zahra Rahiman, Richard Tatenda Chikosi, Alexandra Bench, Marcel Simpson, Natalie Hendricks, Freda-Heléne Leuvennink, Ingebor Jäger, Rowan Duys, Margot Flint, Simphiwe Gumede, Abhilash Nair, Aleta Sibi, Aliya Bhorat, Ayabonga Yedwa, Chloe Ash, Danika Govender, Darren Piaray, Dineo Kotu, Jacob Blou, Jani Gerber, Jenna Piercy, Johann Fourie, Joseph Ruiz von Walter, Joshua Louis, Khelan R Dheda, Khulile Singata, Mahdiya Bhayat, Mayilan Chetty, Nabiha Ebrahim, Naho Khorombi, Nina Mabusela, Olachi Emeruem, Phi Nguyen, Rahul Joseph, Robyn Brown, Samiya Bhorat, Suvina Chanerika, Tiashan Moodley, Zaeem Ebrahim, Zander Skye Isaac, Mariette Grobbelaar, Dave Bishop, Marzanne Nel, Leesa Bishop, Sanele Magagula, Kirsten Marais, Victor Ruhinda, Suhail Sayad, Nerlicia Sewnarain, Aphiwe Mtshengu, Tsakani Mabunda, Sisanda Tiya, Dipo Adeyemi, Muhummed Uwais Moosa, Lwazi Goso, Anika Lucas, Emma Scheepbouwer, Nabeela Motala, Glenda Watt, Nok'zotha Njoko, Gaby Nel, Bonga Mthembu, Melissa Purdon, Previn Johnson, Humeshan Naidoo, Jan Kriel, Howard Wain, Buhlebonke Skhakhane, Asheeq Soobader, Abdur Razaaq Lamera, Melanie Lesch, Cara Dunn, Hilde Turkstra, Marcello Leita, Tajil Manmohan, Natanael Janse van Rensburg, Nkosi Nxumalo, Thandiwe Mchunu, Jisheel Kanaye, Tamsin Singh, Bonga Khoza, Melissa Yolanda Hippolite, Nonhlanhla Zimase, Nonhlanhla Madlala, Stephanie Scriba, Graeme Hofmeyer, Richard Southey, Michaela Peters, Muetu Kapena, Kasandri Govender, Alishka Naidoo, Fiona Lourens, Andisiwe Ngcobo, David Govender, Yasheen Maharaj, Lwazi Mntungwa, Dhivendra Singh, Suman Mewa Kinoo, Ruvashni Naidoo, Kishan Anand Naidu, Nhlakanipho Ngubane, Maaneka Ramadhin, Randolph Green-Thompson, Nyameka Maluleke, Karthik Naidoo, Sne F Mkhize, Patrick Kanana, Brian Ubisi, Mfundo Ndlela, P Hiralal, S Lakhani, Balungile Dzingwe, Andile Mfene, Tumisho Bokgobelo, Nithin Khoon Khoon, Aadi Modi, Chanel Dalais, Zubair Omar, Meegashen Narainsamy, Nombuso Mlawu, Saba Sivuyisiwe, Taskeen Mather, Nozipho P. Mabaso-Langa, B.S.B Mbhele, S.Z Nyawose, J.A Nzimande, P.P Moele, M.B Ndimande, Nonhlanhla N Zulu, Marcin Kopieniak, Christian Kyanda-Kaboza, Farrah Khan, Etienne Venter, Nondumiso Masondo, Bonginkosi Nene, Abiodun Lamina, Nkosi Sibahle, Samukelesiwe Kubheka, Tilou Thobane, Mzamo Mnguni, Andrew Miller, Steyn Botha, Melandie Fourie, Reinhard Schmidt, Chris Westwood, Devandiran Harriraman Rungan, Devarani Naidoo, Adushan Govender, Zizipho Noluthando Madikizela, Siddharth Mohan, Bavna Hira, Keshree Naidoo, Valsura Ramsundar, Brendan Moonsamy, Mlibo Mthembu, Mark Blaylock, Eddie Tembe, Busisiwe Cawe, Michael Ojonimi Ameh, Osahon Daniel Erebor, Sijabulile Cassius Sosibo, Sikelela Siyibane, Charles Choto, Mlamli Dotye, Zolelwa Zibuye Nandi, Sanelisiwe Sivuyile Ngceba, Thulisa Anela Qaziyana, Chwayita Katshwa, Unathi Simamkele Ntshongwana, Sivuyise Gwazela, Lerato Pakade, Siphosihle Msutu, Singleton Luxolo Sandla, Thobeka Portia Ngcobo, Sandiso Yekani, Vuyolwethu Sotashe, Khanya Galela, Abongile Sukwana, Busisiwe Mrara, Lindubuhle Beba, Mawande Mayibenye, Samuel Alomatu, Siqhamo Magadla, Gloria Hyera, Siphesihle Khoza, Lelethu Jwambi, Mzomhle Kiza, Athandiwe Qhonono, Lungisa Petse, Nomvuzo Saqu, Lwandile Mtshabe, Sibi Joseph, Yondela Sidoyi, Banele Kokose, Anele Dayimani, Chwayita Makrexeni, Emma Muendo Loko, Apelele Futshane, Sinesipho Ndedwa, Nosiphiwo Semane, Aphiwe Mqhayi, Jacob Blou, Rahul Joseph, Joseph Ruiz von Walter, Phi Nguyen, Joshua Louis, Chris Pearce, Robin Veitch, Samiya Bhorat, Aliya Bhorat, Dineo Kotu, Margot Flint, Nina Mabusela, Sandile Dube, Makura Ndhlovu, Siphiwe Ngema, Kaajal Brijlall, Thabani Nyathikazi, Kashal Ramsaroop, Ameer Beeharry, Stefan van der Walt, Chiara Anne Baars, Karishma Heeramun, Sabrina Pillay, Xander Botha, Christopher Brits, Chad Young, Andrew Monahan, Mikaeel Abdool, Brendon Chetty, Anele Thabiso Ndlangisa, Asanda Marawu, Phumzile Khuboni, Philiswa Nkondlo, Siphe Xabendlini, Abu Bakr Arbee, Michael Robert Cooke, Hsin-Nua Wang, Jeandré Malherbe, Craig Sydney Scholtz, Michael Santana, Jo Shadwell, Julia Tooke, Paul Martin Pretorius, Zamancwango Mncwango, Luke Miguel Fourie, Daniel McElhenny, Riza Ehlers, Andile Twele, Julia Kenmuir, Rory Livanos, Gregory Wiid, Lwande Gongota, Anele Thabiso, Zaza Shange, Dela Maiwald, Henri van der Merwe, Reena Panicker, Saidur Molla, Sarel Kruger, Sanam Chuturgoon, Laila Choonara, Werner Erasmus, Aashiqui Ramith, Alana Williams, Anathi Sokanyile, Dane Rampini, Emma Howes, Gabriella Kwant, Heilisha Dehaloo, Kaylin Adams, Seelo Mvelase, Mthokozisi Nsele, Ridwa Hajee, Saahil Bhanial, Tajil Manmohan, Sajit Alladeen, Robert Stevenson, Preston Moodley, Shirish Sewpersad, Spheh Ndlovu, Marli Ellis, Rajesh Ramjee, Myint Aung, Prashant Gokal, Omishka Hirachund, Sizwe Zungu, Allison Smith, Wayne Rees, Nishan Pillay, A Hirjee, Khaya Matanzima, Pavan Kishendutt, Ravi Mishra, Yoshua Bwambale, Mphathiseni Dlamini, Dedi Mutondo, Mbayo Yuma, Chane Retief, Wandile Shandu, Precious Mzobe, Tando Mpotulo, Emily du Plessis, Judith Maria Oosthuizen, Nicquin Nervin Adams, Andrea Snyman, Sunnet Ellis, Jacobus Rademan, Lisa Combrink, Herman Hendrik van der Linden, Pieter Gerhardus Marais, Matshidisho Nodoba, Thazriq Eksteen, Yusuf Ameen, Sidharth Singh, Tamilla Nieuwoudt, Michaela Bredenkamp, Nitesh Naranbhai, Anke van der Linden, Adam Asghar, Nazreen Ahmed, Lungile Mthethwa, Zein Elabideen Darwish, Aavishkar Rooplall, Kamintha Govender, Kershan Munien, Mohamed Nadeem Ali, Zahraa Vahed, Thembelihle Pretty Hloni, Siyabonga Ximba, Phumelele Ntinga, Leeyen Singh, Sunhera Sukdeo, Jabulilie Solomon, Nikeziwe Ncwane, Nokwanda Mabaso, Pumla Zondi, Nhlakanipho Shabalala, Tiffany Pratt, Mmapali Mokapela, Chuene Hlahla, Nkateko Chauke, Petrus Jansen van Vuuren, Chloe Howell, Sanjula Pillay, Dineo Mashigwane, Fred Mulunda, Jannes Moolman, André Oosthuizen, Allison Muller, Maryll Stuurman, Zahnne Fullerton, Ziyaad Limalia, Abigail Davies, Kelsey Bester, Margot Flint, Simphiwe Gumede, Mergan Naidoo, Dylan Barnard, Jadon Haridutt, Leigh Coetzee, Shanaaz Fortune, Deveena Jasmin Maharaj, Muhammad Yusuf Sayed Essop, Nicholas Fine, Mazin Mahir, Alhussein Hamid, Abdelfatah Abdelmageed, Sara Abdalla, Shadan Elsayied, Moaj Ahmed, Alamin Ali, Aneth Kaliza, Linda Mlunde, Elibariki Mkumbo, Rafael Shayo, Godfrey Barabona, Happines Biyengo, Edson Gaston Luvakubusa, Atupyane Mati, Simforosa A Atanas, Monica N Lucas, Michael J Munuo, Zulfa S Matola, Greyson Victor Joseph, Asadulillah Kidusi, Scholastica B Bitesigirwe, Dionisia A Mkonga, Jenitha Joseph Cheru, Petronela Malema, Shija Mathias, Maynard Samuel Makere, Chrystal Tarah Munyanyi, Kelvin Fidelis Rutahoile, Linnah Abraham Njau, Rutendo Gina Mutimukulu, Precious Nicole Gunha, Leonard Joseph Mrosso, Serena Silayo, Flora Mbuyu, Joyce Materu, Mary John Chuwa, Mathayo Thomas, Grace Charles, Dina J Lihangaka, Herieth Mwenda, Bebatus Nyamahanga, Cholela Braison Augustino, Pascal Robert Dalama, Remigius Richard Apolinary, Edward Mathias Nsemwa, Magreth Gitige Mangi, Catherine Gatwa, Geofrey Oscary Kayombo, Winfrida Yena Ndebele, Keneth Mashauri Kyando, Stella Maneno Jacob, Sixtus Ruyumbu Safari, Filbert Francis Ilaza, Shafi Hamis, Monica Peter, Rehema Mushi, Sajda Ally, Heneriko Benedicto, Gustavu Mbunda, Reuben Andeshi, Abel Elias, Willium Anstone, Fransesco Aron, Saguda Matondo, Suzana Constantine, Ezekiel Komanya, Samwel Asantaeli, Francis Joseph Gwejo, James Gambaseni, Hubert August Ngowi, Lucky Harran Mkocha, Sesilia Michael, Onesmo Seth Laizer, Dede Dotto Chapajuja, Arserius Rutaiwa, Jackline P Kokuhirwa, Efgenia M Bombo, Domina J Muikila, Leah D Mwelinde, Deusdedith William, Liberatus Mushi, Bonphace B Msimbano, Clement J Mchomvu, Charles Charles Petro, Pendo N Yombo, Tekla Timotheo, Christian L Kusekwa, Servat Ikigijo, Patricia Osward, Ephrem Tahhani, Stamili Mkumba, Senso SURNAME, Hadija Wanguvu, Edwin Edward Ernest, Edwin Edward, Eliab J Daud, Innocent B Sanga, Anna J Kalinga, Rania Ammar, Mounir Bouaziz, Fatma Kolsi, Rahma Daoued, Mouna Jerbi, Noureddine Rekik, Amel Bouzid, Hayet Zitouni, Sahar Elleuch, Salma Toumi, Fatma Khanfir, Sana Omri, Kamel Kolsi, Mohamed Ben Hmida, Moamed Maalej, Kais Chaabane, Riadh Mhiri, Samar Bellil, Houda Belmabrouk, Oussama Jaoued, Imen Bannour, Sawsen Chakroun, Nessrine Ben Saad, Marwen Baccar, Lassaad Sahnoun, John Kiboma Wogabaga, Mercy Logose, Yaya Miriam Jackline, Roggers Odongo, Aruho Moses, Innocent Musiime, Joan Naluyima, Peter Magala, Moses Tommy Wobudubire, Mary Nambuba, Gerald Waniala, Joram Esemu, Getrude Nakiria, Emily Chesang, Zamu Logose, Salim Nabila, Sophie Namasopo, John Kalungi, Isooba Safiyu Ayub, Amon Asindu, Elizabeth Namuyala, Isaac Muyanja, Ivan Mwebesa, Ruth Muhindo, Anthorny Wasukira, Mary Innocent Waswa, Ronald Aruho, Moses Mwebaza Kakooza, Grace Nambuya, Brenda Balungi, Joseph Emuron, Clare Ameri, Roggers Kedi, Constance Amulen, Gladys Nabukenya, Joan Nachuka, Priscilla Mercy Adikin, Peace Draleru, Naume Etoko Akello, Federeth Nabisubi, Hasifah Namutebi, Glades Isina, Jennifer Kipwola, Derrick Muhwana, John Paul Ochieng, Fred Salya, Harriet Patricia Asekenye, Christine Tino, Doreck Nahurira, Assen Kamwesigye, Jude Mulowoza, Ronald Aridriga, Sam Orech, Brenda Namugga, Richard Gamubaka, Fred Maiso, Joshua Orikiriza, Andrew Lemu, Jena Amos Chebet, Moses Chelangat, Bruno Onen Chan, Zainab Kabasemeza, Jane Nakibuuka, Paul Omagor, Lynn Martha Nattabi, Vanessa Nantale Lubulwa, Benard Oyang, Moses Arinaitwe, Eddy Cantong, Vanessa Nanono, Dramanigo Kodjo, Maria Assimwe, Bright Nagaba, Ceasor Julis Owor, Nicholas Kabugo, Emmanuel Eemu, Dickson Kamoga, Charles Emuduko, Sheif Semakula, Cornelius Sendagire, Tomanya Kakuru Kenneth, Susan Nabunya, Joyce Wamala N, Claire Namuwaya, Chelsea Edwards Sanyu, Kenneth Innocent Nyeko, Mwesigwa Lukwago Seezi, Allan Phillip Barigye, Herman Tabula Mpumbu, Derrick Mukurasi, Ronnie Omoro, Maurine Lenia, Catherine Namutebi, Sarah Namatovu, Ndaiziwei Masukhume, Samson Pomo, Abubakar Bala Muhammad, Lofty-John Anyanwu, Datti Alhassan Muhammad, Salahu Dalhat, Bashir Yunusa, Mustapha Ibrahim Usman, Abdulrahman Muhammad, Abdulrazak Ajiya, Ademola Babatunde, Ramadan Mansur Aliyu, Aisha Nalado, Alfa Mika'il Abdullahi, Aliyu Ibrahim, Amal Galadanci, Aminu Abba, Mamuda Atiku, Atiku Jibrilla, Bello Muideen Abodunde, Hamza Muhammad, Ibrahim Musa Idris, Isma'il Jibrin, Baba Ahmad, Kabir Adamu Musa, Maria Assiimwe, Shehu Kana, Mahmoud Kawu Magashi, Mohammad Aminu Mohammad, Musa Zango, Mustapha Miko Abdullahi, Muzzammil Abdullahi, Usman Abubakar Nagoma, Nasiru Ishaq, Oseni Ganiyu, Saminu Muhammad, Shamsudeen Muhammad, Sulaiman Daneji, Idris Usman Takai, Zynat Sani Alhassan, Tijjani Nasiru Nagwamutse, Misbahu Haruna, Abdulrahman Abba Sheshe, Garba Ilyasu, Musa Babashani, Suleiman Abdulrashid, Musa Baba Maiyaki

## Abstract

**Background:**

Critical illness represents a major global health-care burden and critical care is an essential component of hospital care. There are few data describing the prevalence, treatment, and outcomes of critically ill patients in African hospitals.

**Methods:**

This was an international, prospective, point prevalence study in acute hospitals across Africa. Investigators examined all inpatients aged 18 years or older, regardless of location, to assess the coprimary outcomes of critical illness and 7-day mortality. Patients were classified as critically ill if at least one vital sign was severely deranged. Data were collected for the available resources at each hospital and care provided to patients.

**Findings:**

We included 19 872 patients from 180 hospitals in 22 African countries or territories between September, 2023 and December, 2023. The median age was 40 (IQR 29–59) years, and 11 078/19 862 (55·8%) patients were women. There were 967/19 780 (4·9%) deaths. On census day, 2461/19 743 (12·5%) patients were critically ill, with 1688/2459 (68·6%) cared for in general wards. Among the critically ill, 507/2450 (20·7%) patients died in hospital. Mortality for non-critically ill patients was 458/17 205 (2·7%). Critical illness on census day was independently associated with subsequent in-hospital mortality (adjusted odds ratio 7·72 [6·65–8·95]). Of the critically ill patients with respiratory failure, 557/1151 (48·4%) were receiving oxygen; of the patients with circulatory failure, 521/965 (54·0%) were receiving intravenous fluids or vasopressors; and of patients with low conscious level, 387/784 (49·4%) were receiving an airway intervention or placed in the recovery position.

**Interpretation:**

One in eight patients in hospitals in Africa are critically ill, of whom one in five dies within 7 days. Most critically ill patients are cared for in general wards, and most do not receive the essential emergency and critical care treatments they require. Our findings suggest a high burden of critical illness in Africa and that improving the care of critically ill patients would have the potential to save many lives.

**Funding:**

National Institute for Health and Care Research (NIHR) Global Health Group in Perioperative and Critical Care (NIHR133850).

## Introduction

Critical illness has been defined as a state of ill health with vital organ dysfunction, a high risk of imminent death if care is not provided, and the potential for reversibility.^1^ Critical illness is the most severe form of acute illness, and can be due to underlying conditions of every aetiology in every patient group.^1^, ^2^, ^3^ The importance of critical illness is illustrated by the high level of resource provision for critical care in high-income countries. The global incidence of critical illness is estimated at 30–45 million people each year using data from specific diagnoses in a North American intensive care unit registry,^4^ but the true figure might be higher as the majority of patients with critical illness are cared for in general wards and emergency units, not intensive care units.^5^, ^6^, ^7^ Recent World Health Assembly resolutions have emphasised the importance of critical care to resilient health-care systems, and improved population health.^8^, ^9^

Data describing the prevalence of critical illness and its care in Africa are scarce. A recent White Paper by the International Federation for Emergency Medicine and the World Federation of Intensive and Critical Care calls for improved evidence about critical illness and critical care in low-resource settings to enable the strengthening of critical care services.^7^ We know there are far fewer intensive care beds in Africa compared with other parts of the world (<1 per 100 000 population compared with 34 and 29 per 100 000 population in the USA and Germany, respectively).^10^, ^11^, ^12^ However, the burden of critical illness in Africa, the associated outcomes, the current care provided to critically ill patients, and the resources available to manage critical illness remain unknown. As overall disease burdens and mortality rates are high in Africa,^13^, ^14^, ^15^, ^16^ it is likely that critical illness burdens are also high. Data from Malawi suggest that one in five hospital inpatients are critically ill,^17^ and in Tanzania one in ten patients presenting to an emergency unit are critically ill.^18^ The fundamental elements of essential emergency and critical care (EECC) are well described, and include simple therapies such as oxygen, intravenous fluids, and correct positioning of the critically ill patient.^19^ The availability and readiness of EECC resources have only previously been assessed in hospitals in Tanzania.^20^ Data from Malawi suggest that EECC has not been implemented universally as 89% of adults with hypoxia, and 75% of children who died of pneumonia, did not receive oxygen therapy.^21^, ^22^

**Research in context**

**Evidence before this study**

No formal search was done, but a literature review of critical illness in Africa identified only one international study of the burden of critical illness in a mixed cohort of hospitalised patients which included African hospitals. Amongst 3652 patients from two hospitals in Malawi, two hospitals in Sri Lanka, and four hospitals in Sweden, the prevalence of critical illness was 12·0%, with an associated hospital mortality of 18·7%. Importantly, 19 out of 20 critically ill patients were receiving care in a general ward in Malawi rather than in an intensive care unit. The prevalence of critical illness was markedly higher in Malawi and associated with a higher mortality rate than in Sri Lanka or Sweden at 20·6%. We also identified one single-centre study of the prevalence of critical illness in a hospital in Uganda where 11·7% of hospital in-patients were critically ill, with a 7-day in-hospital mortality rate of 22·6%. We found no international studies of the prevalence of critical illness across African hospitals. A recent White Paper by the International Federation for Emergency Medicine and by the World Federation of Intensive and Critical Care calls for improved evidence on critical illness and the current state of critical care in low-resource settings to enable the strengthening of critical care services. Three studies have estimated the number of intensive care beds in Africa to be fewer than 1 per 100 000 population, compared with 34 and 29 per 100 000 population in the USA and Germany, respectively. However, given the very scarce provision of intensive care units in African countries, many patients will not have access to such units and limiting research to intensive care units does not describe the true prevalence of critical illness in African hospitals. Furthermore, despite recommendations of the clinical processes and the resources required to provide essential emergency and critical care (EECC), to our knowledge there are no studies reporting the care provided or the resources currently available across Africa. These data are crucial for health-system planning, both in normal times and when preparing for possible future pandemics and public health emergencies.

**Added value of this study**

The African Critical Illness Outcomes Study (ACIOS) was a point prevalence study of critical illness and 7-day mortality among almost 20 000 adult hospital in-patients across 180 hospitals in 22 African countries or territories. The study has found a large burden of critical illness: 12·5% of in-hospital patients were critically ill, of whom 20·7% subsequently died—compared with 2·7% of the non-critically ill patients. The majority of critically ill patients were cared for in general wards, rather than in intensive care units or high care units. EECC was provided to only half of the patients who needed it.

**Implications of all the available evidence**

The ACIOS findings suggest a large and neglected burden of critical illness, and a high incidence of preventable deaths from critical illness, in Africa. In many cases, the provision of basic critical care through the equitable and systems-based implementation of EECC could have a substantial impact on these preventable patient deaths in Africa, saving many lives from acute diseases of every aetiology.

We undertook the African Critical Illness Outcomes Study (ACIOS) to determine the prevalence of critical illness, the care provided to critically ill patients, and the patients’ outcomes among all adult inpatients in a sample of acute hospitals in African countries. This evidence is needed to inform health policy across the continent of Africa.

## Methods

### Study design and participants

ACIOS was an international, prospective, point prevalence study of critical illness among adult inpatients in acute hospitals in Africa. The study was open to all African countries, and we included all countries and territories that registered and fulfilled national and local ethics and regulatory requirements. Hospitals were recruited through the African Perioperative Research Group and the EECC network. Local investigators in each hospital selected a single day to collect study data within the international study period and collected outcomes on included patients 7 days later. Local investigators only observed participating patients, and did not provide treatment. All patients were managed by clinical staff according to local hospital standards and protocols. However, if local ACIOS investigators observed that a patient needed urgent care, clinical staff were immediately notified. The primary ethics approval was provided by the Human Research Ethics Committee of the University of Cape Town, South Africa (HREC 260/2023). Ethics approval processes varied between countries, with all participating hospitals formally ethically approved for participation. A summary of the ethical approval processes is shown in the appendix (p 42). ACIOS was prospectively registered on ClinicalTrials.gov (NCT06051526). Our findings are reported in accordance with the STROBE statement.^23^

Any acute hospital, regardless of funding mechanism, admitting acutely unwell patients was eligible to participate. Hospitals only admitting patients for elective surgery, psychiatric illness, or rehabilitation were excluded. Where acute hospitals incorporated a ward designated exclusively for patients with psychiatric conditions, the psychiatric ward was excluded. Hospitals were categorised as level 1 (district), level 2 (regional), or level 3 (university, central, or national). All adult patients aged 18 years or over receiving inpatient care in any department or ward in a participating hospital on the day of data collection were included. This included inpatients in the maternity and emergency departments. Patients with a primary psychiatric diagnosis and patients who had not been admitted for in-hospital treatment (ie, outpatients, and emergency department patients who were managed without admission to a hospital ward) were not included. All national ethics committees approved a waiver of informed consent as the dataset only included variables documented as part of routine clinical care. ‘Broadcasting’ signage was used to inform patients and families that the hospital was participating in the study (appendix p 43). All patients were included unless they opted out of participation. Some hospital wards opted out of participation.

### Data collection

Hospital-level data were collected once for each hospital, including health facility characteristics and the available resources for EECC (appendix pp 44–47). Data were recorded on paper case record forms at one point in time, when the clinician investigators were at the patients’ bedside. All vital signs, (respiratory rate, oxygen saturation, blood pressure, heart rate, and conscious level), were measured by the clinician investigators, unless it was not possible in which case they were then taken from the documented clinical observations. All patients were followed up at 7 days to determine death or survival. Data were pseudoanonymised using a unique numeric code before entry onto an internet-based electronic case record form. Identifiable patient data were stored in a locked office in each hospital.

### Outcomes

The coprimary outcome measures were critical illness at the time of assessment, and 7-day in-hospital mortality. Critical illness was defined using an existing definition from an international concept analysis.^1^ Patients were classified as critically ill if one or more vital signs were severely deranged, as in previous international studies.^17^, ^24^, ^25^, ^26^ Severe derangements were defined as respiratory rate less than 8 breaths per minute or greater than 30 breaths per minute, oxygen saturation less than 90% (pulse oximetry), systolic blood pressure less than 90 mm Hg, heart rate less than 40 beats per minute or greater than 130 beats per minute, and reduced conscious level (responsive to pain or unresponsive on the alert, voice, pain, unresponsive scale [AVPU]). For women in active labour, vital signs were assessed between contractions. In a small number of cases where it was inappropriate to measure a particular vital sign, such as blood pressure in a patient on an end-of-life care pathway, the most recent previously recorded value was taken instead. Patients were followed up for 7 days to assess mortality and the secondary outcome of length of hospital stay. An additional secondary outcome was the provision of EECC to critically ill patients. The hospital-level outcome was the availability of resources for EECC within the hospitals.^19^ The case record form and definitions are shown in the appendix (pp 48–49).

### Statistical analysis

There was no prespecified sample size, as we aimed to recruit as many hospitals as possible, and every eligible patient from each hospital. A post-hoc sample size calculation confirmed that the study was adequately powered to enter all 16 candidate parameters into the regression models as the outcome event of mortality exceeded ten events per parameter. Based on an expected in-hospital mortality of 5%,^17^ a sample size of 8000 patients would provide a 95% CI of 1% around the point estimate (ie, 4*·*5–5*·*5%). Data analysis was performed according to a prespecified statistical analysis plan (appendix pp 50–60). The resources available for EECC in the hospitals was calculated as the number of resources always available divided by the total number of EECC resources. Multivariable logistic regression models were constructed to determine the relationship between patient factors and mortality. As this was a pragmatic study, we only collected data on patient (explanatory) factors we considered clinically important for in-hospital mortality (the response variable), and all these factors were entered into the multivariable regression models. The explanatory factors entered into the model were: age, sex, admission category (urgency of admission, and main category of admission: non-communicable disease, trauma, infection, or maternal health), chronic diseases (hypertension, diabetes, cancer, chronic obstructive pulmonary disease or asthma, heart disease, HIV/AIDS, tuberculosis, or other), pregnancy, and critical illness. For the logistic regression models, random effects are assumed to be normally distributed, and the default link function is the logit function. We used a three-level generalised mixed model with patients at the first level, hospital at the second level, and country at the third level, to account for the expected correlation in outcomes within hospitals and countries. All factors were entered into the model, as the number of reported deaths was sufficient to provide ten events (deaths) per parameter. Collinearity was assessed using the variance inflation factor with a cut point of 5. The variance inflation factor did not exceed 2 for any of the variables. No collinearity was detected, and hence no variables were either excluded or combined. The model fit was evaluated. We present the risk of mortality in those with critical illness at the time of census, compared with those without critical illness using the adjusted logistic regression. A post-hoc sensitivity analysis was conducted with a Cox regression to account for time-to-mortality. A Kaplan–Meier graph was constructed for in-hospital mortality for critically ill and non-critically ill patients with the start time as the time of clinical assessment for critical illness, and the end time as 7 days later. We performed a log-rank test to assess the difference between the survival of patients who were, and were not, critically ill. All analyses were complete case analyses without imputation of missing data due to a low rate of missing data (691/19872 [3*·*5%] had at least one missing variable in the regression model). Model diagnostics and fit for all logistic regression models was assessed using simulated residuals generated by the DHARMa package in R. Patients with missing outcomes data were included without imputation and reported descriptively. Data are presented as mean (SD), median (IQR), n (%), or odds ratios (OR) with 95% CIs. Statistical analyses were performed using the Statistical Package for the Social Sciences (SPSS) version 24 (SPSS, Chicago, USA) and R statistical software package version 3.4 (R Foundation for Statistical Computing, Austria) and R packages ‘coxme’ and ‘ggsurvfit’.

### Sensitivity analyses

We conducted the following prespecified sensitivity analyses for the definition of critical illness. We included all patients meeting the primary definition of critical illness above, and also patients receiving a critical care treatment as this could have resulted in physiological correction of a severely deranged vital sign, falsely classifying a patient as not critically ill. We conducted a sensitivity analysis excluding patients with treatment limitations (eg, not for resuscitation in the event of a cardiac arrest, or not for intensive care unit in the event of deterioration). For patients with missing critical illness data, we performed best case and worst case sensitivity analyses where missing data were imputed as normal (ie, critical illness absent) or abnormal (ie, critical illness present), respectively.

### Role of the funding source

The funder of this study had no role in study design, data collection, data analysis, data interpretation, writing of the report, or the decision to submit for publication.

## Results

**Figure 1:**
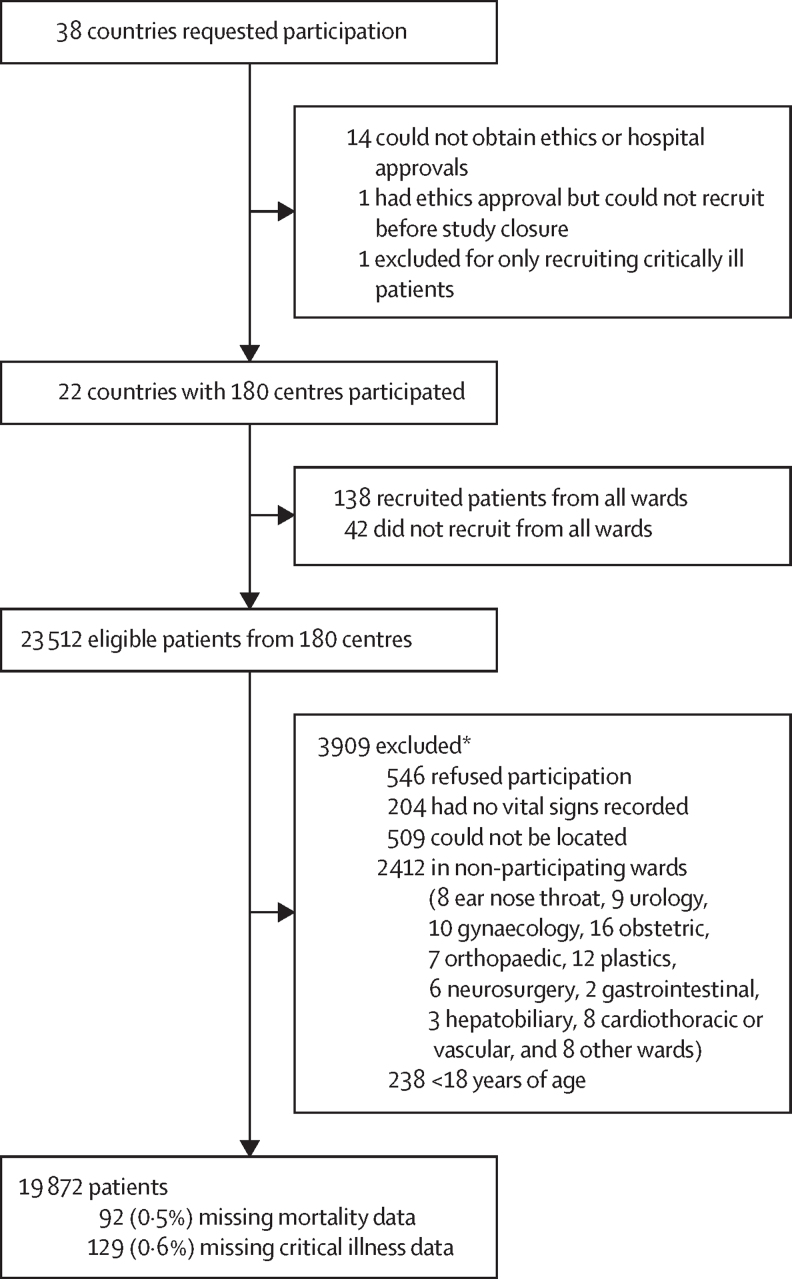
Study profile ACIOS=African Critical Illness Outcomes Study. *Some patients were excluded due to multiple reasons.

Between Sept 6, and Dec 27, 2023, we recruited 19 872 patients from 180 hospitals across 22 countries or territories in Africa (Botswana, Burkina Faso, Congo, Democratic Republic of the Congo, Egypt, Ethiopia, The Gambia, Ghana, Lesotho, Libya, Morocco, Mozambique, Namibia, Nigeria, Somalia, Somaliland, South Africa, Sudan, Tanzania, Tunisia, Uganda, Zimbabwe; figure 1 and appendix pp 61–62). Hospital-level data were provided for 173 (96·1%) of 180 hospitals. There were 56 (32·3%) of 173 level 1 hospitals (representing 2657 [13·3%] of 19 920 participants), 38 (22·0%) of 173 level 2 hospitals (representing 3846 [20·0%] of 19 920 participants), and 79 [45·7%] of 173 level 3 hospitals (representing 12 717 [66·32%] of 19 920 participants). 152 (71*·*4%) of 173 hospitals were government funded, 19 (11·0%) of 173 were privately funded, and five (2*·*9%) of 173 were charitable organisations. Hospitals had a median of 265 (IQR 122–519) standard beds, with 7 (3–16) beds in designated high care units, and 7 (2–12) beds designated as intensive care unit beds.

The median age of all patients was 40 (IQR 29–59) years, and 11 078 (55·8%) of 19 862 patients were women (table 1). Most hospital admissions were acute or emergency admissions (15 115/19 771 [76·4%]) with non-communicable disease being the most common indication for admission (9353/19 794 [47·2%]). Hypertension and diabetes were the most common comorbidities (5015/19 872 [25·2%] and 2668/19 872 [13·4%], respectively). Most admissions were to general wards (17 626/19 814 [89·0%]) and to medical and surgical disciplines (7427/19 864 [37·4%] and 7477/19 864 [37·6%], respectively). Only 807 (4·1%) of 19 814 patients were admitted to an intensive care unit. No patient-level variable had a missingness rate of more than 0·5% (appendix p 64).

**Table 1:** Baseline characteristics

		**All patients (n=19 872)**	**Patients with critical illness (n=2461)**	**Patients without critical illness (n=17 282)**	**Patients with critical illness without treatment limitations (n=2238)**	**Patients with critical illness with treatment limitations (n=223)**	**Patients who died (n=967)**	**Patients who survived (n=18 813)**
Age (years)		40 (29–59)	48 (32–65)	40 (29–58)	47 (32–65)	53 (36–70)	57 (40–71)	40 (29–58)
Sex								
	Male	8784/19 862 (44·2%)	1206/2460 (49·0%)	7513/17 274 (43·5%)	1073/2225 (48·2%)	126/223 (56·5%)	521/967 (53·9%)	8218/18 805 (43·7%)
	Female	11 078/19 862 (55·8%)	1254/2460 (51·0%)	9761/17 274 (56·5%)	1152/2225 (51·8%)	97/223 (43·5%)	446/967 (46·1%)	10 587/18 805 (56·3%)
Known chronic illness or pregnancy								
	Pregnant	2620/19 872 (13·2%)	140/2461 (5·7%)	2468/17 282 (14·3%)	135/2226 (6·1%)	5/223 (2·2%)	11/967 (1·1%)	2600/18 813 (13·8%)
	Hypertension	5015/19 872 (25·2%)	702/2461 (28·5%)	4276/17 282 (24·7%)	637/2226 (28·6%)	64/223 (28·7%)	351/967 (36·3%)	4641/18 813 (24·7%)
	Diabetes	2668/19 872 (13·4%)	378/2461 (15·4%)	2273/17 282 (13·2%)	339/2226 (15·2%)	39/223 (17·5%)	185/967 (19·1%)	2464/18 813 (13·1%)
	Cancer	1226/19 872 (6·2%)	174/2461 (7·1%)	1049/17 282 (6·1%)	150/2226 (6·7%)	23/223 (10·3%)	131/967 (13·5%)	1093/18 813 (5·8%)
	COPD or asthma	791/19 872 (4·0%)	209/2461 (8·5%)	575/17 282 (3·3%)	185/2226 (8·3%)	22/223 (9·9%)	60/967 (6·2%)	730/18 813 (3·9%)
	Heart disease	1291/19 872 (6·5%)	275/2461 (11·2%)	1011/17 282 (5·9%)	245/2226 (11·0%)	27/223 (12·1%)	117/967 (12·1%)	1170/18 813 (6·2%)
	HIV/AIDS	2197/19 872 (11·1%)	310/2461 (12·6%)	1864/17 282 (10·8%)	269/2226 (12·1%)	39/223 (17·5%)	127/967 (13·1%)	2046/18 813 (10·9%)
	Tuberculosis	657/19 872 (3·3%)	171/2461 (6·9%)	480/17 282 (2·8%)	135/2226 (6·1%)	35/223 (15·7%)	61/967 (6·3%)	589/18 813 (3·1%)
	Other	2947/19 872 (14·8%)	474/2461 (19·3%)	2446/17 282 (14·2%)	405/2226 (18·2%)	66/223 (29·6%)	253/967 (26·2%)	2667/18 813 (14·2%)
Urgency of admission								
	Elective	4656/19 771 (23·5%)	285/2449 (11·6%)	4344/17 291 (25·1%)	266/2218 (12·0%)	19/223 (8·5%)	70/964 (7·3%)	4570/18 726 (24·4%)
	Emergency or acute	15 115/19 771 (76·5%)	2164/2449 (88·4%)	12857/17 201 (74·7%)	1952/2218 (88·0%)	204/223 (91·5%)	894/964 (92·7%)	14 156/18 726 (75·6%)
Main category for admission								
	Non-communicable disease	9353/19 794 (47·3%)	1316/2452 (53·7%)	7973/17 220 (46·3%)	1179/2223 (53·0%)	133/223 (59·6%)	596/961 (62·0%)	8722/18 747 (46·5%)
	Maternal health	3741/19 794 (18·9%)	207/2452 (8·4%)	3517/17 220 (20·4%)	203/2223 (9·1%)	4/223 (1·8%)	18/961 (1·9%)	3713/18 747 (19·8%)
	Trauma	3461/19 794 (17·5%)	345/2452 (14·1%)	3099/17 220 (18·0%)	319/2223 (14·3%)	24/223 (10·8%)	90/961 (9·4%)	3350/18 747 (17·9%)
	Infection	3239/19 794 (16·4%)	584/2452 (23·8%)	2631/17 220 (15·3%)	522/2223 (23·5%)	61/223 (27·4%)	257/961 (26·7%)	2962/18 747 (15·8%)
Airway patency								
	Normal	19 201/19 848 (96·7%)	2103/2456 (85·6%)	16 987/17 279 (98·3%)	1938/2233 (86·8%)	165/223 (74·0%)	790/965 (81·9%)	18 320/18 791 (97·5%)
	Partial obstruction	574/19 848 (2·9%)	295/2456 (12·0%)	277/17 279 (1·6%)	246/2233 (11·0%)	49/223 (22·0%)	153/965 (15·9%)	420/18 791 (2·2%)
	Complete obstruction	73/19 848 (0·4%)	58/2456 (2·4%)	15/17 279 (0·1%)	49/2233 (2·2%)	9/223 (4·0%)	22/965 (2·3%)	51/18 791 (0·3%)
Conscious level (AVPU)								
	Alert	18 113/19 846 (91·3%)	1461/2457 (59·5%)	16 550/17 282 (95·8%)	1359/2235 (60·8%)	102/223 (45·7%)	495/966 (51·2%)	17 533/18 788 (93·3%)
	Responds to voice	949/19 846 (4·8%)	212/2457 (8·6%)	732/17 282 (4·2%)	179/2235 (8·0%)	33/223 (14·8%)	183/966 (18·9%)	760/18 788 (4·0%)
	Responds to pain	491/19 846 (2·5%)	491/2457 (20·0%)	0/17 282	444/2235 (19·9%)	47/223 (21·1%)	155/966 (16·0%)	335/18 788 (1·8%)
	Unresponsive	293/19 846 (1·5%)	293/2457 (11·9%)	0/17 282	253/2235 (11·3%)	40/223 (17·9%)	133/966 (13·8%)	160/18 788 (0·9%)
Heart rate								
	Beats per minute	87 (76–99)	100 (84–119)	86 (76–97)	100 (84–119)	100 (83–120)	96 (82–112)	87 (76–99)
Oxygen saturation								
	Percentage	97 (96–99)	95 (89–98)	98 (96–99)	96 (89–98)	93 (89–98)	96 (92–98)	97 (96–99)
Respiratory rate								
	Breaths per minute	20 (17–22)	23 (19–31)	19 (17–21)	22 (19–30)	25 (20–34)	22 (18–28)	20 (17–22)
Blood pressure								
	Systolic blood pressure (mm Hg)	123 (110–133)	113 (92–133)	120 (110–133)	114 (92–133)	110 (92–130)	119 (101–138)	120 (110–133)
	Diastolic blood pressure (mm Hg)	75 (65–83)	70 (58–81)	75 (66–84)	70 (59–81)	68 (56–81)	71 (60–85)	74 (65–83)
Ward type								
	Medical	7427/19 864 (37·4%)	1427/2459 (58·0%)	5939/17 276 (34·4%)	1279/2224 (57·5%)	141/223 (63·2%)	617/966 (63·9%)	6765/18 807 (36·0%)
	Surgical	7477/19 864 (37·6%)	658/2459 (26·8%)	6770/17 276 (39·2%)	597/2224 (26·8%)	56/223 (25·1%)	252/966 (26·1%)	7190/18 807 (38·2%)
	Maternal	3672/19 864 (18·5%)	184/2459 (7·5%)	3472/17 276 (20·1%)	182/2224 (8·2%)	2/223 (0·9%)	15/966 (1·6%)	3646/18 807 (19·4%)
	Other	1288/19 864 (6·5%)	190/2459 (7·7%)	1095/17 276 (6·3%)	166/2224 (7·5%)	24/223 (10·8%)	82/966 (8·5%)	1206/18 807 (6·4%)
Ward level								
	General ward	17 626/19 814 (89·0%)	1688/2459 (68·6%)	15817/17 231 (91·8%)	1551/2225 (69·7%)	131/223 (58·7%)	637/964 (66·1%)	16 922/18 774 (90·1%)
	High care unit	1381/19 814 (7·0%)	350/2459 (14·2%)	1030/17 231 (6·0%)	306/2225 (13·8%)	43/223 (19·3%)	147/964 (15·2%)	1228/18 774 (6·5%)
	Intensive care unit	807/19 814 (4·1%)	421/2459 (17·1%)	384/17 231 (2·2%)	368/2225 (16·5%)	49/223 (22·0%)	180/964 (18·7%)	624/18 774 (3·3%)

Data are n/N (%) or median (IQR). Denominators vary with the completeness of the data. AVPU=alert, voice, pain, unresponsive. COPD=chronic obstructive pulmonary disease.

The complete set of vital signs necessary to define critical illness were reported for 19 743 (99·4%) of 19 872 patients, of which 17 533 (88·8%) of 19 743 were measured by the clinician investigators, and the remainder were recorded from patients’ charts.

There were 2461 (12·5%) of 19 743 critically ill patients (table 2), and of these 1688 (68·6%) of 2459 were in general wards, 350 (14·2%) of 2459 were in high care units, and 421 (17·1%) of 2459 were in intensive care units. Sensitivity analyses indicate that the point prevalence for critical illness ranged from 2847 (14·4%) of 19 745, if patients with normal vital signs but receiving critical care treatment (384 patients) were classified as critically ill, to 2238 (11·3%) of 19 746 when classifying the 223 patients with treatment limitations as not critically ill (table 2).

**Table 2:** Point prevalence of critical illness and mortality

	**n/N (%)**
**Prevalence of critical illness**	
Critically ill patients	2461/19 743 (12·5%)
**Sensitivity analyses**	
Critically ill patients (excluding patients with treatment limitations)	2238/19 746 (11·3%)
Critically ill patients (definition including patients receiving EECC or intensive care treatment)	2847/19 745 (14·4%)
Critically ill patients (best case scenario: missing data, considered not critically ill)	2461/19 872 (12·4%)
Critically ill patients (worst case scenario: missing data, considered critically ill)	2590/19 872 (13·0%)
**7 day in-hospital mortality**	
Mortality (whole cohort)	967/19 780 (4·9%)
Mortality (critically ill patients)	507/2450 (20·7%)
Mortality (non-critically ill patients)	458/17 205 (2·7%)

Denominators vary with the completeness of the data. EECC=essential emergency and critical care.

Overall, 967 (4·9%) of 19 780 patients died. Most deaths occurred in patients with acute admissions (894 [92·7%] of 964), patients admitted for non-communicable diseases (596 [62·0%] of 961), or patients admitted to medical wards (617 [63·9%] of 966). Of the critically ill patients, 507 (20·7%) of 2450 died within 7 days, compared with 458 (2·7%) of the 17 205 non-critically ill patients. Among the critically ill patients, most deaths occurred among patients who had been in general wards on the census day (268 [52·9%] of 507), compared with in high care units and intensive care units (102 [20·1%] of 507; and 137 [27·0%] of 507, respectively). Among the critically ill patients in general wards, 268 (15·9%) of 1682 died, compared with 102 (29·4%) of the 347 critically ill patients in high care units, and 137 (32·6%) of the 420 critically ill patients in intensive care units. To assess the potential impact of non-participating wards on the coprimary outcomes, a post-hoc analysis was conducted comparing hospitals with study participation from all wards with hospitals with some wards not participating in the study. The analysis demonstrated a similar point prevalence of critical illness (1916 [12·6%] of 15 243 patients in hospitals with study participation from all wards and 545 [12·1%] of 4500 patients in hospitals with some wards not participating in the study) and 7-day mortality (747 [4·9%] of 15 273 and 220 [4·9%] of 4507, respectively).

**Figure 2:**
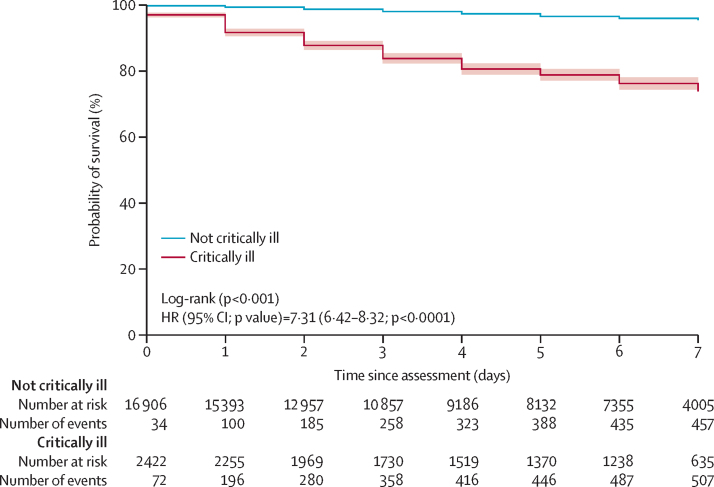
In-hospital survival among critically ill and not critically ill patients HR=hazard ratio. Shaded bands show 95% CI.

Most critically ill patients (2052 [83·8%] of 2450) fulfilled the definition based on one critical illness criterion (ie, level of consciousness, circulatory, or respiratory), with the most common being respiratory criteria (1154 [5·8%] of 19 776; table 3). Mortality was highest among the critically ill patients who fulfilled the level of consciousness diagnostic criteria (288 [36·8%] of 783), and patients who fulfilled two critical illness criteria (144 [40·3%] of 357) or three critical illness criteria (20 [50·0%] of 40), which was more than double that of patients who only fulfilled one criterion of critical illness. The median length of hospital stay was 4 days (IQR 2–6) for all patients, 4 days (2–6) for non-critically ill patients, and 6 days (2–7) for critically ill patients (p<0*·*0001). The Kaplan–Meier curve for in-hospital mortality is shown in figure 2. Patients were censored if they were discharged before 7 days after assessment, or if they were still alive and in hospital at 7 days. This included 1987 (82·0%) of 2422 critically ill patients (1352 discharged, and 635 in hospital at 7 days), and 16 486 (97·5%) of 16 909 non-critically ill patients (12 481 discharged, and 4005 in hospital at 7 days).

**Table 3:** Critical illness categories and outcomes

	**n (%)**
**Critical illness categories**	
Patients defined as critically ill by conscious level criteria	784/19 846 (4·0%)
Patients defined as critically ill by circulatory criteria	965/19 850 (4·9%)
Patients defined as critically ill by respiratory criteria	1154/19 776 (5·8%)
Patients defined as critically ill by one criterion only	2052/2450 (83·8%)
Patients defined as critically ill by two criteria	358/2450 (14·6%)
Patients defined as critically ill by three criteria	40/2450 (1·6%)
**Mortality associated with critical illness categories**	
Death of critically ill patients fulfilling conscious level criteria	288/783 (36·8%)
Death of critically ill patients fulfilling circulatory criteria	163/962 (16·9%)
Death of critically ill patients fulfilling respiratory criteria	240/1146 (20·9%)
Death of critically ill patients defined by one criterion of critical illness	338/2042 (16·6%)
Death of critically ill patients defined by two criteria of critical illness	144/357 (40·3%)
Death of critically ill patients defined by three criteria of critical illness	20/40 (50·0%)

Data are n/N (%). Denominators vary with the completeness of the data.

Critical illness had the strongest association with in-hospital mortality (unadjusted OR 10*·*13 [95% CI 8*·*80–11*·*66], adjusted OR 7*·*72 [6*·*65–8*·*95]; table 4). Other independent associations with mortality included increasing age, cancer, HIV infection, emergency surgery, and admission for infection, non-communicable disease, or trauma. The Cox regression is shown in the appendix (pp 65–66) and is consistent with the logistic regression. A post-hoc decision to conduct a sensitivity analysis excluding the two countries providing more than 10% of the patients to the dataset (Nigeria and South Africa) was also consistent with the overall analysis (appendix pp 67–70). The multivariable logistic regression model showed acceptable fit, and no significant violations of any model assumptions (appendix pp 71–72).

**Table 4:** Unadjusted and adjusted generalised mixed-effects model of factors associated with in-hospital mortality

		**Unadjusted**			**Adjusted**		
		Odds ratio	95% CI	p value	Odds ratio	95% CI	p value
Age per 10 years		1·39	1·34–1·43	<0·0001	1·23	1·17–1·28	<0·0001
Sex							
	Male	1·47	1·29–1·68	0·0001	1·14	0·98–1·32	0·083
	Female	Reference	..	..	Reference	..	..
Known chronic illness or pregnancy*							
	Pregnant	0·07	0·04–0·12	<0·0001	0·64	0·30–1·38	0·25
	Hypertension	1·75	1·53–2·01	<0·0001	1·11	0·93–1·33	0·23
	Diabetes	1·56	1·32–1·85	0·0001	0·96	0·78–1·17	0·66
	Cancer	2·84	2·33–3·48	<0·0001	2·81	2·22–3·54	<0·0001
	COPD/Asthma	1·63	1·24–2·16	0·0006	0·79	0·58–1·08	0·13
	Heart disease	2·07	1·68–2·55	<0·0001	1·10	0·86–1·39	0·45
	HIV/AIDS	1·39	1·12–1·72	0·0024	1·36	1·07–1·73	0·013
	Tuberculosis	2·08	1·57–2·76	<0·0001	1·15	0·83–1·60	0·49
	Other	2·09	1·80–2·44	<0·0001	1·70	1·43–2·02	<0·0001
Urgency of admission							
	Emergency or acute	4·13	3·22–5·30	<0·0001	3·04	2·34–3·95	<0·0001
	Elective	Reference	..	..	Reference	..	..
Main category for admission							
	Infection	18·54	11·49–29·90	<0·0001	4·79	2·55–8·97	<0·0001
	Non-communicable disease	14·77	9·25–23·57	<0·0001	3·89	2·10–7·22	<0·0001
	Trauma	5·65	3·40–9·39	<0·0001	2·37	1·24–4·44	0·009
	Maternal health	Reference	..	..	Reference	..	..
Critical illness							
	Critically ill	10·13	8·80–11·66	<0·0001	7·72	6·65–8·95	<0·0001
	Not critically ill	Reference	..	..	Reference	..	..

COPD=chronic obstructive pulmonary disease.

* Reference: absence of risk factor.

Of the critically ill patients, 557 (48·5%) of 1148 defined as critically ill by respiratory criteria were receiving oxygen, 521 (54·0%) of 965 defined as critically ill by circulatory criteria were receiving intravenous fluids or vasopressors, and 387 (49·4%) of 784 defined as critically ill by conscious level were receiving an airway intervention or were placed in the recovery position (table 5). Data for patient position at time of examination are shown in the appendix (p 63). All indicated EECC treatments required to manage critical illness were provided in 1092 (44·4%) of 2461 critically ill patients, with 1369 (55·6%) of 2461 critically ill patients only receiving partial or no EECC treatment.

**Table 5:** The EECC treatments given to critically ill patients

	**Critically ill patients (n=2461)**
**EECC treatment given for critical illness**	
All indicated EECC treatments given	1092/2461 (44·4%)
Partial or no indicated EECC treatments given	1369/2461 (55·6%)
**Patients defined as critically ill by respiratory criteria**	
Receiving oxygen	557/1148 (48·5%)
**Patients defined as critically ill by circulatory criteria**	
Receiving intravenous fluids	514/965 (53·3%)
Receiving vasopressors	82/965 (8·5%)
Receiving intravenous fluids or vasopressors	521/965 (54·0%)
**Patients defined as critically ill by conscious level criteria**	
Receiving an airway intervention	328/781 (42·0%)
Placed in the recovery position	79/784 (10·1%)
Receiving an airway intervention or placed in the recovery position	387/784 (49·4%)

Data are n/N (%). Denominators vary with the completeness of the data. EECC=essential emergency and critical care.

The resources available for EECC are shown in the appendix (pp 73–76). Hospitals had a median of 54 (80·6%) of 67 resources (IQR 44–63) available for EECC. Only 13 (7·5%) of 173 hospitals had all the EECC resources available. The availability of all the resources for each EECC domain (ie, equipment, consumables, drugs, human resources, training, guidelines, and infrastructure) ranged from a low of 31 (18·0%) of 172 for the EECC consumable domain to a high of 121 (69·9%) of 173 for the EECC human resources domain (appendix p 76). Training was low (39 [22·5%] of 173), management guidelines were only available in a third of hospitals (63 [36·4%] of 173), access to all drugs required for EECC was absent in half the hospitals (93 [53·8%] of 173), and the equipment and consumables necessary were available in less than a third of hospitals.

On the advice of peer reviewers, a post-hoc decision was taken to present the prevalence of critical illness and 7-day mortality by the Human Development Index of the participating countries to demonstrate the impact of country resources on critical illness and mortality in Africa (appendix p 77). Countries with a low Human Development Index had a higher prevalence of critical illness and mortality when compared with middle and upper Human Development Index countries.

## Discussion

To our knowledge, this is the first epidemiological study of critical illness across Africa. By including data from 180 hospitals across 22 countries or territories, we provide robustly generalisable data describing the prevalence, care provision, outcomes, and the resources available for critical illness in Africa to inform health policy across the continent. The principal finding of this study is that one in eight inpatients in acute hospitals in Africa are critically ill, and one in five of these patients subsequently die. Two-thirds of these critically ill patients are managed in general wards rather than in high care units or intensive care units. We found that the provision of the most fundamental care of critical illness is low, with only half of patients requiring fluid resuscitation, airway management, or oxygen therapy receiving the necessary treatments. Our data confirm the shortage of resources in terms of the hospital infrastructure, equipment, staffing, training, treatment guidelines, consumables, and drugs required to treat critically ill patients effectively. Our overall findings suggest a high incidence of preventable deaths from critical illness in Africa.

Critical illness is a challenging field of epidemiological study, particularly in low-resource environments. In high-income countries, the burden of critical illness is often defined by the number of patients receiving care in an intensive care unit, or a similar enhanced care facility. However, the definition of an intensive care bed varies widely between countries as does the number of intensive care beds. Consequently, the calculated burden of critical illness is artificially inflated in resource-rich health systems with generous critical care provision.^27^ Meanwhile, in most African acute hospitals, there are few critical care beds^10^, ^11^, ^12^ and this study shows that most critically ill patients are treated in general hospital wards. We identified only two previous small studies in Africa with which to compare our findings,^17^, ^18^ and one hospital-wide study of the prevalence of critical illness in a high-income country.^5^ Our definition of critical illness is sensitive,^5^, ^17^, ^25^ is endorsed by the International Federation for Emergency Medicine and the World Federation of Intensive and Critical Care,^7^ and can be pragmatically operationalised by identifying patients with one or more severely deranged vital signs. Although this approach might be considered by some to overestimate prevalence, our study confirms that in a risk-adjusted model this definition of critical illness is independently associated with in-hospital mortality, with an adjusted OR of 7·72, and over 20% of critically ill patients died in-hospital (ie, this definition identifies a very high-risk group of patients). Crucially, our definition identifies patients whose outcomes could be improved by the most fundamental critical care actions which do not necessarily require admission to a high care or intensive care unit.

Improving the care of critically ill patients throughout hospitals, likely through being a higher priority in the health system, training clinical staff in EECC, ensuring the fundamental resources are available, and improving the processes of care in general wards, could have a substantial impact on patient outcomes across medical specialties, particularly as our study demonstrates there is a short supply of resources and poor provision of EECC treatments. EECC should be prioritised by key stakeholders, for example to underpin efforts towards universal health coverage; included in national health benefit packages; used in the global operationalisation of the 2023 World Health Assembly resolution 76.2 on integrated emergency, critical, and operative care; and included in strategies, recommendations, and guidelines by global health funders, institutions, and professional societies.

Efforts to improve the care of critical illness should align closely with sepsis and HIV initiatives. One in four critically ill patients in our study had infection as the main category of admission and one in eight had known HIV. Infection and HIV were associated with 7-day mortality among all patients, (adjusted OR 4·79 and 1·36, respectively). Infectious diseases are an important and preventable cause of critical illness and mortality with an estimated 48·9 million incident cases of sepsis and 11·0 million sepsis-related deaths each year.^28^ Another finding from the study is that nearly one in ten critically ill patients have treatment limitations. The need for palliative care and pain relief in Africa is likely to be substantial.^29^

A strength of this study is that every patient in the participating hospitals was assessed by a clinician investigator to identify critical illness,^1^ by using patient physiology, and not defined by the patient's diagnosis or the area within the hospital in which they were being treated.^30^ By including data describing 95% of hospital inpatients in 180 hospitals across 22 African nations, we have provided a robust and highly generalisable dataset to inform ongoing research and improvements to health policy. The findings can be used to highlight a neglected area of health policy and practice, and to strengthen the care of critically ill patients in countries in Africa. Our sensitivity analyses confirm minimal bias in the findings of our primary analyses. This study does have some limitations. Although we approached collaborators in 38 countries, only 23 countries were able to secure research ethics approval in time to take part, therefore the data might not be representative of all African countries. We excluded data from one country because data were only collected describing patients who met the definition of critical illness. In addition, there were some wards in the hospitals which were eligible for ACIOS but unable to participate. Our experience from previous continent-wide studies suggests that some countries, hospitals, and wards are often unable to participate due to insufficient infrastructure and research resources. This is likely to reflect the low resources in these health systems, hospitals, and wards, which could be an indicator of worse patient outcomes than in those that were able to participate. Data were collected between September and December and there might be seasonal variations in some locations. However, Africa is a large geographical area with many different climate zones, and we do not expect that similar changes in critical care burdens would occur across all involved countries at the same time. Our data only reflect the prevalence of critical illness on a single day in each hospital and might be affected by a range of local factors including weather, public holidays, transport failure, and armed conflict. The point prevalence method might provide a lower estimate of critical illness burden than would other methods such as a period prevalence over 24 hours, as it misses patients who are stable at the time of data collection but were critically ill either before or after this timepoint. Patients with habitually deranged vital signs due to chronic diseases could have been misclassified as critically ill, however the prevalence of this is low and would not impact the findings. Finally, we did not define the characteristics of the intensive care units, but rather collected data on the areas designated as intensive care units in the participating hospitals. However, the level of care provided in areas designated as intensive care units in Africa might be low, as described in Ethiopia.^31^ It is possible that some of the hospitals that reported no high care beds or intensive care beds could have had beds that were closed to clinical care at the time of the study due to resource constraints. A further limitation of the analysis includes unmeasured confounding due to socioeconomic status and access to health care, which impact in-hospital outcomes.

In conclusion, the prevalence of critical illness and associated mortality is high in hospitals in African countries. One in eight hospital inpatients is critically ill, of whom one in five patients die. Most critically ill patients are cared for in general hospital wards. Critically ill patients frequently do not receive the fundamental treatments they require to avert mortality. Critical illness has been neglected in health-care policy, research, and implementation, and improving the care of critically ill patients has the potential to save many lives from acute diseases of every aetiology.

### The ACIOS Investigators

### Contributors

### Data sharing

Data sharing requests are welcome from bona fide researchers from 2 years after publication of ACIOS and will be considered by the ACIOS Steering Committee. Requests should be sent to the corresponding author.

## Declaration of interests

TB declares technical consultancies with UNICEF, the World Bank, USAID, and PATH, all outside the submitted work. GB has received scholarships from PainSA, the National Research Foundation (South Africa), and the Oppenheimer Memorial Trust, speakers’ fees for talks on pain and rehabilitation, and travel grants for conferences from the University of Cape Town and National Research Foundation (South Africa). COS has received travel support from WHO for attending critical care workshops. RMP has received research grants and honoraria from Edwards Lifesciences. All other authors declare no competing interests.
