# Department of Error

**DOI:** 10.1016/S0140-6736(25)00688-9

**Published:** 2025-04-12

*The African Critical Illness Outcomes Study (ACIOS) Investigators. The African Critical Illness Outcomes Study (ACIOS): a point prevalence study of critical illness in 22 nations in Africa.* Lancet *2025; **405:** 715–24*—In the appendix of this Article, the names of the ACIOS Investigators have been updated. This correction has been made to the online version as of April 10, 2025.

