# Supplementary material for: The African Critical Illness Outcomes Study (ACIOS): a point prevalence study of critical illness in 22 nations in Africa
Source: Lancet. 2025 Mar 1;405(10480):715–24. doi: 10.1016/S0140-6736(24)02846-0 (PMC11872788; doi:10.1016/S0140-6736(24)02846-0)
Supplement: Supplementary appendix [file mmc1.pdf]

THE LANCET

Supplementary appendix

This appendix formed part of the original submission and has been peer reviewed. We post it as supplied by the authors.

Supplement to: The African Critical Illness Outcomes Study (ACIOS) Investigators. The African Critical Illness Outcomes Study (ACIOS): a point prevalence study of critical illness in 22 nations in Africa. *Lancet* 2025; **405:** 715–24.

APPENDIX

The African Critical Illness Outcomes Study (ACIOS):
a point prevalence study of critical illness in 22 nations in Africa

The African Critical Illness Outcomes Study (ACIOS) Investigators

Table of Contents

Members of the African Critical Illness Outcomes Study (ACIOS) Investigators 2

Steering Committee: 3

Supplementary Material S1. Ethical approval processes for the African Critical Illness Outcomes Study 42

Supplementary Material S2. Broadcasting document 43

Supplementary Material S3. Hospital data record form 44

Supplementary Material S4. Case record form 48

Supplementary Material S5. African Critical Illness Outcomes Study (ACIOS) definitions 49

Supplementary Material S6. The African Critical Illness Outcomes Study (ACIOS) Statistical Analysis Plan . 50

Supplementary Material S7. Participating countries, hospitals and participants recruited 61

Supplementary Material S8. Patient position at time of examination 63

Supplementary Material S9. Data missingness for the generalised linear mixed model 64

Supplementary Material S10. Unadjusted and adjusted Cox mixed effects model of factors associated with in hospital mortality 65

Supplementary Material S11. Unadjusted and adjusted generalized mixed effects model of factors associated with in hospital mortality. Sensitivity analysis: excluding the Nigerian and South African cohorts. 67

Supplementary Material S12. Unadjusted and adjusted Cox mixed effects model of factors associated with in hospital mortality. Sensitivity analysis: excluding the Nigerian and South African cohorts. 69

Supplementary Material S13. Model diagnostics and fit for the logistic regression model 71

Supplementary Material S14. Resources available for the provision of Essential Emergency and Critical Care (EECC) 73

Supplementary Material S15. Summary of domain resources available for Essential Emergency and Critical Care (EECC) 76

Supplementary Material S16. Point prevalence of critical illness and mortality in the African Critical Illness Outcomes Study (ACIOS) by the Human Development Index (HDI) of participating countries 77

Members of the African Critical Illness Outcomes Study (ACIOS) Investigators

Writing Committee: Tim Baker[1], Juan Scribante[2], Muhammed Elhadi[3], Adesoji O Ademuyiwa[4], Babatunde Osinaike[5], Christian Owoo[6], Daniel Sottie[7], Karima Khalid[8], Adam Hewitt-Smith[9], Arthur Kwizera[10], Fitsum Kifle Belachew[11], Degsew Dersso Mengistu[12], Yared Boru Firissa[13], Tirunesh Busha Gemechu[14], Gaudencia Dausab[15], Unotjari Kauta[16], Kaveto Sikuvi[17], Nahla Kechiche[18], Kelan Bertille Ki[19], Martin Mukenga[20], Dolly Munlemvo[21], Mustapha Bittaye[22], Abubacarr Jagne[23], Mohamed Abdinor Omar[24], Hassan Ali Daoud[25], Mohamed Faisal[26], Mahmoud Elfiky[27], Mpho Seleke[28], Tarig Fadalla[29], Alshaima Koko[30], Alemayehu Bedada[31], Gilles Niengo Outsouta[32], Marie Elombila[33], Ahmed Rhassane El Adib[34], Meryem Essafti[35], Dino Lopes[36], Atilio Morais[37], Pisirai Ndarukwa[38], Newten Handireketi[39], Fred Bulamba[40], Busisiwe Mrara[41], Hyla-Louise Kluyts[42], Marian Kinnes[43], Gillian J Bedwell[44], Hanel Duvenage[45], Gwendoline Arendse[46], Luke Hannon[47], Landon Myer[48], Anneli Hardy[49], Carl Otto Schell[50], Rupert M. Pearse[51], Bruce M Biccard[52]

1. Tim Baker, PhD, Department of Emergency Medicine Muhimbili University of Health and Allied Sciences, Dar es Salaam, Tanzania; and Department of Global Public Health, Karolinska Institutet, Stockholm, Sweden; and Queen Mary University of London, United Kingdom; and Clinical Research Department, London School of Hygiene and Tropical Medicine, UK.
2. Juan Scribante, PhD, Associate Professor, Surgeons for Little Lives, Department of Paediatric Surgery, University of the Witwatersrand, Johannesburg, South Africa.
3. Muhammed Elhadi, MBBCh, Faculty of Medicine, University of Tripoli, Tripoli, Libya.
4. Professor Adesoji O Ademuyiwa, FACS, Professor of Surgery (Paediatric and Surgical Epidemiology), Department of Surgery, College of Medicine, University of Lagos and Lagos University Teaching Hospital, Lagos, Nigeria.
5. Professor Babatunde Osinaike, FMCA, Professor of Anaesthesia and Intensive Care, University of Ibadan/University College Hospital, Ibadan, Oyo State, Nigeria.
6. Christian Owoo, MV, MBChB, FGCS, MPH, Senior lecturer, University of Ghana Medical School; Director of the Medical Training and Simulation Centre, University of Ghana Medical Centre; and Consultant Anaesthesiologist & Head of ICU, Korle Bu Teaching Hospital, Accra, Ghana.
7. Daniel Sottie, MBChB, DA (WACS), FGCS (Anaesthesia), Korle-Bu Teaching Hospital, Accra, Ghana.
8. Karima Khalid, MD, MMed, Department of Anaesthesia and Intensive Care, Muhimbili University of Health and Allied Sciences, Dar es Salaam, Tanzania.
9. Adam Hewitt-Smith, MBBS, Lecturer, Faculty of Health Sciences, Busitema University, Busitema, Uganda, Queen Mary University of London, United Kingdom, EC1M 6BQ.
10. Arthur Kwizera, MBChB, M.Med, Makere University, Kampala, Uganda.
11. Fitsum Kifle Belachew MSc Med, Network for Perioperative and Critical Care, Debre Berhan University Asrat Woldeyes Health Sciences Campus, Debre Berhan, Ethiopia and Global Surgery Division, Department of Surgery, University of Cape Town, Cape Town, South Africa.
12. Degisew Dersso Mengistu, MPH, National Emergency, Injury and Critical Care Program Coordinator, Ministry of Health, Ethiopia
13. Yared Boru Firissa, MD, ALERT Comprehensive Specialized Hospital, Addis Ababa, Ethiopia.
14. Tirunesh Busha Gemechu, MD, Honorary staff, Black Lion Hospital, Addis Ababa, Ethiopia.
15. Gaudencia Dausab, MMED(STEL), Ministry of health and social services Intermediate Hospital Katutura Consultant, Windhoek, Namibia.
16. Unotjari Kauta, MBBS, Ministry of Health and Social Services, Senior Medical Officer in the Medical Intensive Care Unit Intermediate Hospital Katutura, Windhoek, Namibia.
17. Kaveto Sikuvi, FCEM (SA), Ministry of Health Namibia, Lecturer, University of Namibia School of Medicine, Windhoek, Namibia.
18. Nahla Kechiche, MD, Associate Professor, Department of Pediatric Surgery, University Hospital of Monastir, Monastir, Tunisia.
19. Kelan Bertille Ki, MMed, Associate Professor, Joseph Ki-Zerbo University, CHU Pédiatrique Charles de Gaulle, Ouagadougou, Burkina Faso.
20. Martin Mukenga, MD, Specialist in Anesthesiology, University Hospital of Kinshasa, KINSHASA, Democratic Republic of Congo.
21. Dolly Munlemvo, MD, Specialist in Anesthesiology, University Hospital of Kinshasa, KINSHASA, Democratic Republic of Congo.
22. Mustapha Bittaye, FWACS, University of The Gambia, SerreKunda. The Gambia.
23. Abubacarr Jagne, MBchB, , Fellow WACP and ISN fellow of Nephrology, Deputy Chief Medical Director and Chair of Medical Advisory Committee EFSTH, and lecturer, University of The Gambia, SerreKunda, The Gambia.
24. Mohamed Abdinor Omar, MBBS, Global Surgery Emergency Department Federal Ministry of health and Human Services Somalia, Mogadishu, Somalia.

25. Hassan Ali Daoud, MD, Amoud University, Geneva Graduate Institute, Somaliland.
26. Mohamed Faisal, MD, College of Medicine and Health Science, University of Hargeisa. Somaliland Emergency Medicine Association, Hargeisa, Somaliland.
27. Professor Mahmoud Elfiky MD, Cairo University, Cairo, Egypt.
28. Mpho Seleke, MBBCh, District Medical Officer, Ministry of Health, Lesotho.
29. Tarig Fadalla, MBBS, Ribat University Hospital, The National Ribat University, Khartoum, Sudan.
30. Alshaima Koko, MBBS, Pediatric Surgery Center, Ribat University Hospital Khartoum, Sudan.
31. Alemayehu G Bedada, MD,Associate Professor, University of Botswana, Gaborone, Botswana.
32. Gilles Niengo Outsouta, MD, Hospital Practitioner in Polyvalent ICU at CHU Brazzaville, Brazzaville, Congo.
33. Marie Elombila, Professeur Agrégé, Université Marien Ngouabi CHU de Brazzaville, Brazzaville, Congo.
34. Professor Ahmed Rhassane El Adib, MD, Head of Anesthesia and Critical Care in Gynecology-Obstetrics Department, Mohammed VI University Hospital, Cadi Ayyad University, Marrakesh. Dean of Faculty of Medicine, Mohammed VI University of Sciences and Health, Casablanca, Morocco.
35. Meryem Essafti, MD, Assistant professor, Anesthesia and Critical Care in Gynecology-Obstetrics Department, Mother and Child Hospital, Mohammed VI University Hospital, Faculty of Medicine and Pharmacy of Marrakech, Cadi Ayyad University of Morocco.
36. Dino Lopes, MD, Maputo Central Hospital, Tutor of the Emergency Medicine residency, Maputo, Mozambique.
37. Atilio Morais, PhD, Director de Departamento de Cirurgias, Hospital Central de Maputo, Faculdade de Medicina, Universidade Eduardo Mondlane, Maputo, Mozambique
38. Pisirai Ndarukwa, PhD, Bindura University of Science Education, Bindura, Zimbabwe and University of KwaZulu Natal, KwaZulu Natal, South Africa.
39. Newten Handireketi, MSc, Bindura University of Science Education and National Institute of Health Research, Ministry of Health and Child Care-Zimbabwe, Harare, Zimbabwe.
40. Fred Bulamba, MMed, Department of Anaesthesia and Critical Care, Faculty of Health Sciences, Busitema University, Tororo, Uganda, William Harvey Research institute, Queen Mary University of London, London, United Kingdom.
41. Busisiwe Mrara, MMed, Department of Anaesthesiology, Nelson Mandela Academic Hospital, Walter Sisulu University (WSU), Mthatha, South Africa
42. Hyla-Louise Kluyts, MMed (Anaes), Department of Anaesthesiology, Sefako Makgatho Health Sciences University, Ga-Rankuwa, South Africa.
43. Marian Kinnes, Department of Anaesthesia and Perioperative Medicine, Groote Schuur Hospital, Faculty of Health Sciences, University of Cape Town, Cape Town, South Africa.
44. Gillian J Bedwell MSc, Department of Anaesthesia and Perioperative Medicine, Groote Schuur Hospital, Faculty of Health Sciences, University of Cape Town, Cape Town, South Africa.
45. Hanel Duvenage MSc Molecular Medicine and Bioinformatics, MSc Nutrition, Research and Development Manager, Safe Surgery South Africa.
46. Gwendoline Arendse, MPH, research assistant, Department of Anaesthesia and Perioperative Medicine, Groote Schuur Hospital, Faculty of Health Sciences, University of Cape Town, Cape Town, South Africa.
47. Luke Hannan, MPH, statistical consultant, Division of Epidemiology and Biostatistics, School of Public Health, Faculty of Health Sciences, University of Cape Town, South Africa.
48. Professor Landon Myer, PhD, Professor, statistical consultant, Division of Epidemiology and Biostatistics, School of Public Health, Faculty of Health Sciences, University of Cape Town, South Africa.
49. Anneli Hardy, MS Statistics, statistical consultant, Department of Statistical Sciences, Faculty of Science, University of Cape Town, Cape Town, South Africa.
50. Carl Otto Schell, PhD, Department of Global Public Health, Karolinska Institutet, Stockholm, Sweden, Centre for Clinical Research Sörmland, Uppsala University, Eskilstuna, Sweden, Department of Medicine, Nyköping Hospital, Sörmland Region, Nyköping, Sweden.
51. Professor Rupert M Pearse MD(Res), Professor of Intensive Care Medicine, Queen Mary University of London, United Kingdom, EC1M 6BQ.
52. Professor Bruce M Biccard PhD, Professor and 2nd Chair, Department of Anaesthesia and Perioperative Medicine, Groote Schuur Hospital, Faculty of Health Sciences, University of Cape Town, Cape Town, South Africa.

Steering Committee:

NIHR Global Health Group on Perioperative and Critical Care, co-Directors and Senior Investigators: Rupert M Pearse, Bruce M Biccard

Chief Investigator: Tim Baker

Statistical analysis: Bruce M Biccard, Gwendoline Arendse, Luke Hannan, Landon Myers, Anneli Hardy

Data administrators: Hanel Duvenage

APPRISE (African Partnership for Perioperative and CRitIcal Care ReSEarch) Project Management (NIHR133850):

Mohammed Adem, Elvis Amunyo, Gwen Arendse, Tim Baker, Upendo Bandeke, Godfrey Barabona, Gillian J Bedwell, Tizeta Belachew, Ermiyas Belay, Aneel Bhangu, Bruce Biccard, David Bishop, Happines Biyengo, Yared Boru, Fred Bulamba, Turunesh Busha, Marcelle Crowther, Lucy Cunnama, Alma O Damasy, Selam Daniel, Justine Davies, Abiy Dawit, Brook Demissie, Degsew Dersso Mengistu, Yamkela Desemela, Kokeb Desta, Kelem Desta, Priyanthi Dias, Hanel Duvenage, Rowan Duys, Robert Dyer, Ryan Ellis, Charles Fadipe, Margot Flint, Alexander Fowler, Aphelele Futshane, Desta Galcha, Richard Gamubaka, Nuroadis Guchima, Simphiwe Gumede, Thomas Hamborg, Anneli Hardy, Adam Hewitt-Smith, Sabra Hussein, Anna Hvarfner, Anab F Issa, Kasandji Kabambi, Aneth Kaliza, Assen Kamwesigwe, Muzamiru Kawiso, Peniel Kenna, Karima Khalid, Justine Khanyalano, James Kidulu, Fitsum Kifle, Tewodros Kifleyohanes, Marian Kinnes, Juma Kitwara, Herbert Kiwalya, Hyla-Louise Kluyts, Charles Machumu, Edson Mafana, Peter Magala, Salome Maswime, Rev. Mzubanzi Mdunyelwa, Borislava Mihaylova, Elibariki Mkumbo, Linda Mlunde, Catherine Mokotla, Steve Molaoa, Lebogang Moloi, Jolene Moore, Busisiwe Mrara, Lwandile Mtshabe, Betelehem Mulye, Joanitah Nakibuule, Joan Naluyima, Hasifah Namutebi, Rose Nandutu, Juliana Nanimambi, Ezile Ninise, David Okanya, Rupert Pearse, Linda Pohl, Rosalind Prinsloo, Rafael S Shayo, Timothy Stephens, Nicola Vickery, Cecilia Vindrola, John Walutsyo, Bezaye Zemdkun.

ACIOS Investigators:

Hospital Lead Investigators*

Botswana
National leader: Alemayehu Ginbo Bedada

Princess Marina Hospital
- Shingirai Muzondiwa*
- Kagiso Wadikonyana
- Tariro Mubika
- Wabotlhe Ntwayapelo

Burkina Faso
National Leader: Kelan Bertille Ki

Centre Hospitalier Universitaire Yalgado Ouédraogo, Ouagadougou (CHUYO)
- Abdramane Ouattara*
- Papougnezambo Bonkoungou
- Lankoundé Martin

Centre Hospitalier Universitaire de Tengandogo, Ouagadougou (CHUT)
- Salam Savadogo*
- Nathanael Bamogo
- Bayé Nébié

Centre Hospitalier Universitaire Sourô Sanou (CHUSS)
- Ismael Guibla*
- Daouda Barro
- Latifa Aida Sawadogo
- Guy Thierry Ki
- Bego-wene Isai Bado
- Ibrahim Alain Traore

Centre Hospitalier Universitaire de Bogodogo
- Salah Idriss Séif Traore*
- Wendelamita Lydie Rosine Kaboré
- Salifou Napon
- Arouna Louré
- Marame Diop

Congo
National Leader: Gilles Niengo Outsouta, Marie Elombila

CHU de Brazzaville
- Marie Elombila*
- Otiobanda Gilbert Fabrice
- Niengo Outsouta Gilles
- Mpoy Emy Monkessa Christ Mayick
- Iwoba Rebet Sarrah Armanda
- Koumou Banga Fernand Régis

Democratic Republic of Congo
National Leader: Dolly Munlemvo, Martin Mukenga

Biamba Marie Mutombo Dikembe
- Jean Jacques Kalongo*

Centre Hospitalier Initiative Plus (CHIP)
- Patrick Mukuna*
- Frank Nguvulu*

Cliniques Universitaires de Kinshasa
- Berthe Barhayiga*
- Patrick Mukuna
- Martin Mukenga

Clinique Kinoise
- Jeremie Kalambayi*

El Rapha Clinic
- Ted Likongo*

Egypt
National Leader: Mahmoud Elfiky

Aswan University Hospital
- Sarah Magdy Abdelmohsen*
- Mohie El-Din Mostafa Madany

Giza International Hospital
- Ahmed Saber Abdelrahman*
- Sarah Mansour

Ethiopia
National Leaders: Tirunesh Busha Gemechu, Yared Boru Firissa, Fitsum Kefle, Degisew Mengistu

AaBET Hospital
- Ayalew Zewdie Tadesse*
- Daniel Adugna Dashura
- Alemayehu Beharu
- Seble Ashagre Awoke

Alert Hospital
- Tsegay Gebreanenia Hagos*
- Eyuel Teshome
- Amare Yohans Mekonen
- Abnet Ewnetie Emirie

Arbaminch University Hospital
- *Desta* Galcha Gerbu*
- Weldemichael Yigebahal Belay
- Aregahegn Mulgeta Munka
- Begashaw Melesse Dicha
- Addisu Tesfaye Koster
- Moges Tessema Hesbeto

Dilla University Teaching Hospital
- Zerihun Tesfaye*
- Wondimu Dori
- Roba Kebede
- Mihret Kaleb
- Selamawit Yizelkal
- AbrahamT/markos

Haramaya University Hiwot Fana Comprehensive Specialized Hospital
- Melaku Getachew*
- Fanta Wondimneh
- Tilahun Teshager
- Gamachu Bekuma

Hawassa Hospital
- Emnet Tesfaye Shimber*
- Kindie Nigatu Woubshet
- Kibru Kifle Karicha
- Melaku Teshale

Jimma University Medical Center
- Gemechis Melkamu Fufa*
- Addis Ketemaoboro
- Mastewal Yiseni Yimer
- Tilahun Mebre Defersha
- Tewodros Assefa Gossa

Wolkite University Hospital
- Dereje Zewdu*
- Tilahun Temesgen
- Khedir Fikadu
- Shimelis Getu
- Sime Assefa
- Feleke Habte

The Gambia
National Leaders: Mustapha Bittaye, Abubacarr Jagne

Bundung Maternal and Child health Hospital (BMCH)
- Dado Jabbie*
- Marie Saal Mendy
- Isatou Bobo Jallow
- Fanta Fofana

Farafenni General
- Lamin FTT Fadera *
- Bubacarr Jabbie
- Karimadi Fofana
- Divine Nko-Onye Moseri

Edward Francis Small Teaching Hospital (EFSTH):
- Sheikh Omar Bittaye*
- Fatou Jatta
- Anna Donkor
- Awa Mboob
- Abubacarr Jagne
- Fatou Jallow

Kanifing General Hospital
- Kajali Camara*
- Majula Ceesay
- Lamin Sanyang
- Kawsu Fatty
- Muhammed Touray
- Lamin O Beyai
- Ebrima A Jallow
- Modou Lamin Conteh
- Bubacarr Sowe
- Roheyatou Jawara
- Ndumbeh Manneh

Ghana
National Leaders: Christian Owoo, Daniel Sottie

Cape Coast Teaching Hospital, Cape Coast
- Oluwayemisi Esther Ekor*
- Emmanuel Owusu Ofori*
- Patience Koggoh
- Luke Adagrah Aniakwo
- Evans Kofi Agbeno
- Ganiyu Adebisi Rahman
- Kwasi Agyen-Mensah
- Thomas Agyen
- Martin Tangnaa Morna
- Michael Nortey
- Makafui Yigah
- Ansumana Bockarie
- Temabore Victoria Daboner
- Yvonne Ayerki Nartey
- Abigail Serwaa Boateng
- Prince Arthur
- Quainoo Arnold Kofi
- Papa Kojo Mbroh
- Edwina Okaikai Obodai

Dunkwa Municipal Hospital (DMH)
- John Benjamin Annan*
- Kwaku Nti Acquah
- Kwadwo Amoah
- Gloria Ababio
- David Sackey
- Emmanuel Tagoe
- Jonathan Kinphul

Eastern Regional Hospital, Koforidua (ERHK)
- Arko Akoto Ampaw*
- Foster Amponsah-Manu

- Philip Lartey
- Gabriel Attipoe-Djagmah
- Ijeoma Aja
- Francis Wuobar
- Audrey Addy
- Kwesi Edwin-Tenkorang
- Ransford Aduah
- Denutsui Etornam Rhoda
- Ekuamoah Violet
- Esther Adjei-Acquah
- Agnes Avorwulanu
- Ruth Dzifa Ntumi
- Charles Edem Tsivanyo
- Tetteh A. Kwame
- Pinto Henry Kabukie
- Andrea Asante

Effiduase Government Hospital (EfGH)
- Kingsley Damilare Adeoye*
- David Antwi-Agyei
- David Abass
- Mercy Asare
- Kwame Danquah Diaw

Ejisu Government Hospital (EjGH)
- Isaac Asenso Brobbey*
- Rose Mensah
- Dominic Awuah
- Afia Kyere

Ga East Municipal Hospital (GEMH), Accra
- Ebenezer Oduro-Mensah*
- Roxana Segborwotso
- Marion Amo-Mensah
- Reuben Grumah
- Alberta Twumasi-Boakye
- Aubrey Tigwii
- Selywn Agyemang
- Mercy Akoto
- Agnes Amonoo-Mends
- Martha Dsane-Lamptey

Greater Accra Regional Hospital (GARH), Accra
- Ambe Obbeng*
- Henrietta Nana Serwaa Fiscian
- Simeon Biney
- Dorcas Osei-Poku
- Nana Ama Christian
- Frederickson Pobee
- Dorinda Norbetha Lee
- Magdalene Boamah
- Ayishetu Ndeogo
- Mamm-Mavies Tsotsoo Tetteh
- Adu-Brempong Carl
- Zelda Robertson
- Linda Addai
- Gideon Appiah-Boadu
- Al-Hassan Dasana Andani
- Salamatu Nantongma

- Joseph Oliver-Commey
- Lawrence Ofori-Boadu

Komfo Anokye Teaching Hospital (KATH), Kumasi
- Irene Bandoh*
- Akwasi Antwi-Kusi
- Moses Siaw-Frimpong
- Eunice Aboagye
- Sophia Naa Ayikailey Ankrah
- Stephen Opoku Ofori
- Pearl Nyarko Korang
- Samuel Nana Prempeh Agyeman-Gyebi
- John Adabie Appiah
- Yaw Ampem Amoako
- Felix Owusu Osae
- Stephen Sarfo
- Kwaku Gyasi Danso
- Oppong Philip Peprah
- Martha Poku
- Maame Temah Appiah-Berko
- Ebenezer Akomea Agyin
- Anita Eseenam Agbeko
- Paa Ekow Hoyte-Williams
- Emmanuel Osei Kankam
- Joseph Bonney
- Shiela Afua Kedze
- Dennis Dwomoh Nkrumah
- Eva Adu-Boakye
- Yaw Larbi
- Kwabena Addow Opare-Addo
- Amanda Badjo Amankwata

Korle-Bu Teaching Hospital (KBTH), Accra
- Christian Owoo*
- Kwadwo Opoku-Darko*
- Daniel A. Sottie
- Pokua Sarpong
- Grace-Imelda Obeng-Adjei
- Lorraine Baffour-Awuah
- Ernest Aniteye
- Desmond Seshie
- Simitsewa Amoo-Aidoo
- Adjoa Ofei
- Supriya Wassiamal
- Anita Ohenewa Yawson
- Nancy Asiedua Larbi
- Kwame Afriyie Gyamera
- Kelvin Asamoah-Agyepong
- Kwaku Obeng
- Naa Martekuor Vanderpuye
- Cornelia Quarcoopome
- Khadijah Bandawu
- Aisha Sumaila
- Nuhaila Alhassan
- Lydia Apraku-Peprah
- Leslie I. Adam-Zakariah
- Kwadwo Darko
- Bless-Michael Bonney
- Raphaela Agyarko

- Felicia Birch Freeman
- Henry Kwesi Bulley
- Nana Serwaa Agyeman Quao
- Steve Blankson
- Aba Yorke
- Priscilla Allotey
- Theophyllus Addo
- Gifty Addo
- Theodore Boafor
- Maryanne Zuolo
- Kofi Adzi Gudugbe
- Henry Kumi
- Ken Atobrah-Apraku
- Eric Tetteh
- Efua Thompson
- Jerry Coleman
- William Klah
- Michael Ntumy
- Kwaku Asah-Opoku
- Gordon Amoh
- Nicholas Siklere
- Precious Owoo
- Eunice Nyankah
- Faisal Adjei
- Elvis Ohemeng-Mensah
- Jefferson Owusu-Adae
- Prince Larvie
- Clarence Beareba Basogloyele
- Latif Saiba
- Dela Dinku
- Ernest Yorke
- Yaw Ofori-Adjei
- Jane Afriyie-Mensah
- Fiifi Duodu
- Beatrice Baaye
- Woedem Tettey
- Akosua Agyen Frimpong
- Vishnu Abayateye
- Kwame Darko
- Michael Kwapong-Nyarko
- Solomon Atindama
- Joachim Amoako
- Kenneth Baidoo
- Estella Bilson-Amoah
- Eugene Owusu-Achaw
- Evans Sefa Asiedu
- Daasebre Ahensan
- Kow Entua Mensah
- Innocent Adzamli
- Charles Kofi Amoah
- Desrie Gyan
- Matthew Owusu Boamah
- Paa Kwesi Blankson
- Prudence Nutsuklo
- Daniel Baddoo
- Isaac Asiedu
- Dela Fiagbe
- Esinu Akosua Agbeli
- Kingsley Abankwa

- Patrick Bankah
- Omane Acheamfour Okrah
- James Evans Mensah
- George Darko Brown
- Doreen Anderson
- Elikem Ametepe
- Geoffrey Birikorang
- Priscilla Vandyke
- George Nketiah
- Alfred Seedah
- Adejoke Aiyenigba
- Barbara Boi
- Serwah Amoah
- Comfort Gaituah
- Edith Ntumy
- Imoro Braimah Zeiba
- Rachel Kumi Adamson
- Nana Yaa Asuama Afful
- Emily Martha Nortey
- Freda Amoateng
- Senyo Fumador
- Priscilla Agyemang-Duah
- Esther Brobbey
- Samuel Antwi Oppong
- Alfred Edwin Yawson

Kumasi South Hospital
- Kwame Ofori Boadu*
- Rita Larsen-Reindorf*
- Emmanuel Sam Kwabena Baffoe
- Fredrick Gyamfi Apraku
- Nana Takyiaw Borki Owusu
- Margie Kyei
- Yaw Akyina

Kumawu Government Polyclinic
- Alex Agbanu*
- Joshua Dennis Krah
- Osei Amponsah-Kwatiah

Manhyia District Hospital
- Kwame Danso*
- Kamarudeen Korku Hussein
- Appiah Minka
- Rosemond Boah
- Louis Osei
- Hajara Alhassan
- Sarah Dwomoh
- Ernestina Sarfo
- Rita Amponsah

Maternal and Child Health Hospital, Kumasi
- Prosper Kwaku Gbekor*
- Laila Adutwum
- Yaa Bema
- Mabel Osei-Tutu

Nkawie Toase Government Hospital (NTGH)
- Eric Kwame Detoh*

- Seth Obiri
- Michael Gyamfi
- Paul Afriyie
- Seth Asante Egyin

SDA Hospital, Kwadaso
- Randolph Baah Adu*
- Victor Vandel Adjadeh
- Albert Amissah Asiedu

Swedru Municipal Hospital (SMH), Swedru
- Julius Abuku*
- Joycelyn Maame Esi Darkwah
- Richard Dankwah
- Edmund Oko Codjoe
- Dorcas Rockson
- Bernard Nyamekye Antwi
- Gifty Obo-Ninsin Baidoo

Trauma and Specialist Hospital, Winneba (TSHW)
- George Kwame Prah*
- Petrina Bingab
- Emmanuel Koomson
- Emmanuel Ghartey
- Frederick Duah Yao
- Panyin Benyimah Avemee
- Anyemedu Asare Fredovich
- Isaac Korku Akotoye
- Josephine Okine
- Agnes A. Anane
- Akosua Owusu Sarpong
- Marion Okoh-Owusu

University of Ghana Medical Centre (UGMC), Accra
- Amoako Duah*
- Kwame Ekremet*
- Nana Akosua Oppong-Nkrumah
- Joseph Tanlongo
- Dela Andrea Nutsugah
- Kordai Mould
- Susan Siabi
- Kwame Anim-Boamah

Libya
National Leader: Muhammed Elhadi

Abosleem Tripoli Hospital
- Mohamed Fathi Khmera*
- Aya Rasem Khmir
- Abdulrahman Mohammed Alghziwi

Abusitta Hospital for Respiratory Diseases
- Khayri Karban*
- Areej Dakshi

Al-Afia Clinic (AAC)
- Mohamed Saleh Addalla*
- Taha Khaled Elfaituri
- Taha Alkabat

- Ahmed Muhsin Alatiweel
- Mohammed Salaheddin Dabaie
- Abdulhamid Shaban

Aldiaa Hospital
- Ali Abdulnasir Kredan*
- Abdulalim Ramadan Kuridan
- Abdurrahman Abdussalam Haddud

Alfardous Clinic
- Ahmed Eldeeb*
- Ahmed Jreibi

Alfath Medical Center
- Nafati Taher Alnafati*
- Nabeel Ateeyah Faraj Atiyah

Aljala Hospital
- Mohamed Naser Lawgali*
- Aml Emran Khalleefah Alwirfili
- Mustafa Khalil Abdallah Elrgeig
- Rawan Othman
- Fatma Ali IKday

Aljalaa Maternity Hospital (AMH)
- Marwa I M Shoukrie*
- Msara Jamal Haider
- Majdi Ehmeda S Kamil
- Ameerah Ali Hassan Rahoumah

Al-Khoms Teaching Hospital
- Kamla Ali*
- Honayda Almuakkif
- Shahed Alaref
- Aya Haddad
- Amal Nasser
- Maram Abdulgani

Almarj Teaching Hospital
- Alsnosy Abdullah Khalefa Mohammed*
- Osama Alemenefie

Alshahid Attia Alkasah General Hospital
- Ahlaam M. Ali Ayad*

Alshaimaa Clinic
- Ahmed Elfaituri*
- Anwar Salah Hussain Mohamed
- Reem Abdullah Salim Alkikli
- Alaedden Abdalla Akhmag
- Saleh Abdulla Hadia

Al-Zawia Teaching Hospital
- Salim Almaqtouf*
- Mohamed Alsori Alharari
- Ali Alsouri Alharari
- Rawia Adel Mohamed Draa
- Abdulmunem Mustafa Olu
- Marwa Almabrouk Alazomi

- Randah Dhu Omar Aldeeb

Benghazi Medical Center
- Arwi Kara*
- Ayoub Akwaisah
- Mohamed Al Gharyani
- Mostafa El Awami
- Aya Essa
- Abtisam Alharam
- Aeshah Aboukaleesh
- Fatimah Aboukaleesh
- Noura Kareem
- Narjis Husayn
- Mohamed Adel
- Fatimah Mohammed Alenani
- Iesra Eldagheili
- Abdul-muhaymin Almuquryaf
- Rabab Alkurghali
- Sara Alsaeiti
- Hadeil Abd Elaziz
- Salma Akhlaif
- Fawzia Alferjani
- Wijdan Sayfulnasr
- Dalal Salah
- Muna Denini
- Ahmed Ahmayda
- Saja Almugla
- Salwa Ali Awidat
- Abdulsalam Albadri
- Fatima Elfeituri
- Arwi Adrees
- Mohammed Abosedra
- Ameerah Abraheem
- Fathiah Elferjani
- Malak Barghathi
- Yasmin Elmgawob
- Amani Zoubi
- Nourfan Altarhouni
- Asia Bolifa
- Malak Areibe
- Ehab Othman
- Suhaib Issa
- Tasnim Hasan
- Salem Senussi
- Marwah Aleidhah
- Fadelalla Elmozoghi
- Nisren Alsalme
- Fatma Elashhab
- Almotasem Bellah Elsharif
- Abdulmuez Abdulmalik
- Rana Shembesh
- Eman Bureziza
- Sara Abdelmaged
- Hana Faraj
- Najla Alaguri
- Mohammed Salem
- Mohamed Alamrony
- Mustafa Amraja
- Mohammed Elferjani

- Mohammed Alabeedi

Brak General Hospital
- Mohammad Yahmad
- Mubarka Alzarouq

Crown Healthcare Clinical (CHC)
- Hamida El Magrahi*
- Abir Ben Ashur
- Salem Ali

Gharyan Central Hospital (GCH)
- Hibah Bileid Bakeer*
- Akram Alkaseek
- Haitam Shames
- Aya Alqaarh
- Hashim Aborkhis
- Widad Nouralddeen Faraj Amhimmid
- Hala Misbah Ali
- Taha Husayn Alhadi Alfeeras
- Sundes Daba
- Ahmed Abdurrahman Algeblawi

Misrata Medical Center
- Abdulwahab Alzeldeen Abdalei
- Fatima Asedeq Abdulali
- Ahmed Ismael Saleh
- Abdulhamid Mohamed Alailesh
- Mohamed Moftah Assalhi
- Mohanad Taher Bintaher
- Boshra Basher Hashim
- Weam Mohammed Drah
- Abdelaziz Mahjub Gobbi
- Eman Mohammed Abdalhafit Alabani
- Hamdan Bashir Hilan
- Omar Mohamed Ertaiba
- Ahmed Muftah Taweel

National Heart Centre, Benghazi
- Wesal Tarik Yahya Hamad *
- Hadia Mohamed Omar Aldilfaq
- Aya Adam Hassan Mousa

Preventative Medicine Hospital
- Abdurraouf Abusalama*
- Kusay Ayad
- Abdurrahim Elzoubi
- Mohammed Altarabulsi
- Alabas Almigheerbi
- Ahmad Alfayad
- Hatim Elgbaili
- Abdulmuhaymen Elmeshrgie
- Mohammed Albashri
- Mouna Ahmed A Abuhshaima

Sabha Medical Center
- Khadeja Mohammed Mohammed Alawal
- Essraa Ali Alhudhiry
- Anwaar Ali Othman Eshnaf

- Safia Adem Abdulla
- Firdous Assadeq Mllie
- Mona Abdu Alsalam
- Etidal Ali Abuanniran
- Bushra Qezo

Sabratah Teaching Hospital
- Sara Egreara*
- Nabila Abdalkader
- Huda Abdelmajed
- Hajer Abdulhamid
- Ghazala Abouklaish
- Ahmed Alajeeli
- Khawla Alalage
- Ibtehaj Albaewi
- Malak Albusayifi
- Azhaar Aldeeb
- Ali Algarradhi
- Najwa Alkowash
- Safa Almaakef
- Mohammed Almahjoubi
- Hana Altam
- Masarah Ateeyah
- Sahar Azzaz
- Hajir Bahroun
- Doha Blaou
- Safa Barayik
- Bilqays Habhab
- Mohamed Hassan
- Saja Khalifa
- Aya Maiw
- Yasmeen Meelad
- Safiya Mohamed
- Ali Omar
- Ali Tirihbat

Tabarak Private Hospital
- Saifaleslam Elsahli*
- Abdulwahhab Fakroun

Tobruk Medical Center
- Manal A. Mohammed Zaglam
- Areej Alhassan Abdullah Benghuzi
- Shaymaa Moftah Salem
- Sari Sulayman Abdulhafith
- Hana Adrees Hasan
- Najwa Ibrahim
- Mohammed M. M. Abdaljalil
- Eman Moftah Faraj
- Amani Mousay Abdulmawlay Mohammed
- Malak Mashery
- Eman Ahmed Moftah
- Halima Soliman Abdualnabi
- Eman Gaith Ahwaia
- Fatama Mohamed Salem Selaman
- Esra Abdulaziz Hamad Albarani
- Dua Aljali
- Aminah Eid Abd Alsameea
- Morad G Rahel

- Murajia Mahmoud
- Aisha Mabrouk
- Marzouga Mabrouk
- Rehab Rejab
- Ayyah Emran

Tripoli Central Hospital
- Anas Mohammed Aboutartour*
- Batool Ahmed Abdulkarim
- Buthuynah Mohammed Alsaeh Alqahwash
- Laila Ramadan Askar
- Nada Ali Omran Dhem
- Amer Mohamed Abdulaziz Mosbah
- Khalid Asad Bin Qanad
- Abdurraouf Musbah Khalifa Said
- Qabs Madi
- Amal Ibrahim Furjane Khalifa
- Mohamed Hamed Said
- Abdulrhim Omar Mohammed Almeshri
- Marwa Muftah Daloub
- Yara Talal Mohammed Bariun
- Ahmed Nureddin Ben Shaban
- Dareen Ahmed Elmahdi
- Dawoud Amhimmid Saeid
- Firas Nureddin Hammas
- Mahad Mahmoud Qaeim
- Feras Mohamed Shneib
- Nouri Khalefah Afheej

Tripoli University Hospital
- Muad Fathi Abuhallalah*
- Abdalla Mustafa Abdalla Hdidan
- Abdulmalik Ibrikat
- Aisha Abdulfatah Gehimi
- Aisha Alwarfally
- Randah Dhu Omar Aldeeb
- Mawada Mohammed Elgeriane
- Rabeeah Abd Aldaem AbuAlneeran
- Abdulhamid Mohammed Alsagheer
- Safa Zaydan Ammar
- Arwa Tawfeeq Abdulnabi
- Bassam Erhoma
- Mohamed Mustafa Elghazal
- Mohamed Abdusalam Elhaderi
- Mohammmed Yousef Elimselati
- Esra Abdulhafed Abuaen
- Esra Ben Zahra
- Abduladim Omran Ezaddin
- Hidaya Badri Dozan
- Wadad Mohammed Jomaa
- Lobna Shawesh
- Mosab Emhemmed Matous
- Ghaida Abdalla Naana
- Nada Abdulmonam Elhoush
- Ola Abdulqadir Alsharif
- Rawia Adel Mohamed Draa
- Ritaj Mahmoud Ghasem Agha
- Reyam Abdalla Naana

- Abdulsabur Mohamed Salih
- Sanabil Mansour Abdullah
- Serag Lameen Almzainy
- Tasneem Musbah Fara

Zliten Medical Center (ZMC)
- Najat Ben Hasan*
- Abdulmalik Abeed
- Hajer Abusnina
- Majdolin Miloud Almahjoub
- Radhwan Alsaedi
- Lubnah Alsunousi Mohammed Alokshi
- Manal Almaqrahi
- Hudi Dalaf

Zwara Maritime Hospital
- Fahed Gareb*
- Sehar Naji Ashini
- Mwada Tarek Mehdi

Lesotho
National Leader: Mpho Seleke

Mafeteng Government Hospital
- Malefane Moysamai*
- Mpata Seleso
- Mpine Maqolo

Paray Mission Hospital
- Maphiri Ramafikeng*
- Tina Ntee Ntsane
- Thato Molahlehi
- Amohelang Moreki
- Lerato Mohlalisi
- Molahlehi Makhalanyane
- Noi Khoeli
- Lebohang Lebina

Morocco
National Leader: Ahmed Rhassane El Adib, Meryem Essafti

Hopital Arrazi, CHU Mohammed VI
- Samia Errami*
- Soulaimane Laaziri
- Ayoub Bousselham
- Iltimass Gouazar
- Mohamed-Sami Melouane
- Hind Essalim
- Rim Belarbi
- Rihab Belfouzy
- Hasna Laghmami
- Oumama Boujidi
- Salma Amahmid
- Riyad Ghailan
- Said Errahouy
- Adam Mohamed Aajly
- Oussama Ennamra
- Nouha Smily

- Raouia Fares
- Abdellah Agnaou

Mozambique
National Leader: Dino Lopes, Atílio Morais

Hospital Central de Maputo (HCM)
- Dino Lopes*
- Elka Nhaduco
- Francisco Taimo
- Nadia Estafeira
- Onésia Lucia Sérgio Chitsembe Mombassa
- Sebastião Moisés
- Mouzinho Saide
- Cesaltina Lorenzoni
- Luis Gonçalves Ferrão

Namibia
National Leader: Kaveto Sikuvi, Unotjari Kauta, Gaudencia Dausab

Intermediate Katutura Hospital
- Gaudencia Dausab
- Nasjtasha Pieterse
- Anna Hangula
- Fhenny Moongo
- Natasha Nghitukwa
- Rejoice Makongwa
- Dhruvin Das
- Njohela Mwandemele
- Christian Ndambi
- Larisa Jafta
- Hilma Uugwanga
- Maria Sibolile
- Seuna Karuaihe

Katima Mulilo State Hospital
- Bitoma Thotho Amisi*
- Munikasu Christopher
- Sinvula Lutombi
- Masongo Annie
- Matomola Chuma
- Chipman Kamilla
- Nzwile Alphoniso
- Sisamu Mwiza Daphine
- Kawana Concilia
- Estha Masasa
- Lilungwe Sonnety
- Njahi Kaiba

Keetmanshoop District Hospital
- Evans Sanga*
- Rebecka Martin
- Hilma Namuhuya
- Vute Nekwaya
- Loide Johannes

St Mary's Hospital
- Goodman Uushona*
- Daylight Manyere

- Elina Muulu
- Lindah Kabende
- Joseph Chinanga

Walvis Bay District Hospital
- Chloe Paulse*
- Natasha !Gontes
- Hambeleleni Kamati

Windhoek Central Hospital
- Frans Nambinga
- Loide Namwandi
- Nasjtasha Pieterse
- Anna Hangula
- Fhenny Moongo
- Natasha Nghitukwa
- Faith Dzenga
- Dhruvin Das
- Njohela Mwandemele
- Christian Ndambi
- Ndapunikwa Nghihalwa
- Justina Shikongo
- Tracy Mweti
- Larisa Jafta
- Hilma Uugwanga
- Melissa Spiegel
- Maria Sibolile
- Seuna Karuaihe

Nigeria
National Leader: Adesoji O. Ademuyiwa, Babatunde Osinaike.

Abubakar Tafawa Balewa University Teaching Hospital
- Ibrahim Salim Abdullahi*
- Auwal Adamu
- Rabiu Mohammed Bashir
- Abubakar Muhammad Ballah
- Isa Bashiru Ibrahim
- Yusuf Raiyanu Jasawa
- Aliyu Bashir Adamu

Ahmadu Bello University Teaching Hospital
- Saidu Yusuf Yakubu
- Bilkisu Adamu
- Oluseyi Oyebode Ogunsua
- Shafaatu Ismail Sada
- Tunde Talib Sholadoye
- Alfa Yakubu
- Halima Olufunmilola Abdulsalam
- Fomete Benjamin
- Samuel Isa Gana
- Abdulkadir Muhammad Kabiru
- Hamisu Yakubu
- Rabiu Isah Mohammed
- Sufyan Ibrahim
- Umma Suleiman Bawa
- Ganiyat Ronke Olagunju
- Babangida Salahu Mohammed

- Mohammad EL-Amin Idris
- Michael Odigbo
- Fatima Mahmud-Ajeigbe
- Aghadi Ifeanyi Kene
- Tasiu Saadu
- Sheidu Owuda Abdullahi
- Rabi'at Muhammad Aliyu
- Mudi Awaisu
- Chitumu Dotiro
- Stephen G. Gana
- Lucy. E. Okwajebi
- Muhammad Daniyan
- Abdulghaffar Adeniyi Yunus
- Olutayo A. Gana
- Oguntayo Olanrewaju Adekunle
- Muhammad Lawal Abubakar
- Ibrahim Ibrahim Lawal
- Atiku Likunga Atiku
- Ukwubile Linus
- Ahmad Bello
- Ahmad Tijjani Lawal
- Aminu Muhammad Balarabe
- Anisah Yahya
- Shehu Toro Muhammad
- Emmanuel Raphael Abah
- Fakuta Tathiya Naiwa
- Hussaini Yusuf Maitama
- Nwoye Ugo Daniel
- Elizabeth Ogboli-Nwasor
- Abdullahi Sudi
- Muhammad Raji Mahmud
- Ugwu Euphemia Mgbosoro
- Igele Agom Cletus
- Abass Oluwaseyi Ajayi
- Emmanuel Paktama Bwala
- George Duke Mukoro
- Habibu Balarabe
- Sani A. Abubakar
- Bilqis O. Muhammad
- Ademola O. Adeleye
- Sunday O Ajike

Alex Ekwueme Federal University Teaching Hospital

- Donatus O. Egwu*
- Richard L. Ewah
- Promise O. Ubanatu
- Cletus U. Onwe
- Nathan O. Mbawike
- Joshua A. Adebayo
- Chijioke Udu
- Nneka N. Chiege
- Stephen O. Nwafor
- Stephen C. Eke
- Pearl C. Eke
- Chinyere S. Onoka
- Juliana Chi-Nwogo
- Mary Okoyari
- Oluchi F. Ogah

- Chinenye T. Agbo
- Obiageli A Obi
- Mercy N. Nwangwu
- Loveth N. Ugochukwu
- Kelechi Adindu
- Stella A Ugochukwu
- Obiora C. Bernard
- Sunday V. Nwali

Aminu Kano Teaching Hospital

- Abubakar Bala Muhammad*
- Lofty-John Anyanwu
- Datti Alhassan Muhammad
- Salahu Dalhat
- Bashir Yunusa
- Mustapha Ibrahim Usman
- Abdulrahman Muhammad
- Abdulrazak Ajiya
- Ademola Babatunde
- Ramalan Mansur Aliyu
- Aisha Nalado
- Alfa Mika'il Abdullahi
- Aliyu Ibrahim
- Amal Galadanci
- Aminu Abba
- Mamuda Atiku
- Atiku Jibrilla
- Bello Muideen Abodunde
- Hamza Muhammad
- Ibrahim Musa Idris
- Isma'il Jibrin
- Baba Ahmad
- Kabir Adamu Musa
- Shehu Kana
- Mahmoud Kawu Magashi
- Mohammad Aminu Mohammad
- Musa Zango
- Mustapha Miko Abdullahi
- Muzzammil Abdullahi
- Usman Abubakar Nagoma
- Nasiru Ishaq
- Oseni Ganiyu
- Saminu Muhammad
- Shamsudeen Muhammad
- Sulaiman Daneji
- Idris Usman Takai
- Zynat Sani Alhassan
- Tijjani Nasiru Nagwamutse
- Misbahu Haruna
- Abdulrahman Abba Sheshe
- Garba Ilyasu
- Musa Babashani
- Suleiman Abdulrashid
- Musa Baba Maiyaki

Babcock University Teaching Hospital

- Omotayo Felicia Salami*
- Clifford Imonitie

- Usman Kolawole Ajayi
- Ayodeji Emmanuel Babalola

Federal Teaching Hospital Iddo-Ekiti
- Abiodun Idowu Okunlola*
- Cecilia Kehinde Okunlola
- Segun Alex Atolani
- Olumuyiwa Ariyo
- Tesleem Olayinka Orewole
- Olakunle Fatai Babalola
- Adedayo Idris Alawu
- Paul Olukayode Abiola
- Omagbeitse Henry Abiyere
- Augustine Adebayo Adeniyi
- Adewumi Bakare
- Ajayi Adeleke Ibijola

Federal Teaching Hospital Katsina
- Ibrahim Salisu*
- Auwal Mohammed Abdullahi
- Naziru Garba Shuaibu
- Ibrahim O. Shuaibu
- Akeem Ibiyemi
- Jafar Halliru
- Sanusi Bala
- Sani Kasim Inuwa
- Gamaraddeen Abdullahi Muhammed
- Auwal Haruna
- Amina Umar Akeel
- Yahaya Lawal Kankara

Irrua Specialist Teaching Hospital
- Irene Irenosen Akhideno*
- Kelvin Salami
- Peter Itua
- Patrick Iniaghe
- Rachael Izekor
- Christian Uanzekin
- Ehizojie Fidelis

Jos University Teaching Hospital
- Samuel Isaiah Nuhu*
- Henry Yammoh Embu
- Mangai Audu Ngeh
- Kefas Thomas Malau
- Musa Abdullahi Aliyu
- Husseina Amina Aliyu
- Udoka Okorie

Lagos State University Teaching Hospital
- Oluwaseun David Oladokun*
- Obashina Ayodele Ogunbiyi
- Oluwayemisi Bamidele Oluwadun
- Oluwafunmilayo Aderemi Ikotun
- Akintayo Olugbemi Ogunjuboun
- Yetunde Adebimpe Oyeyode
- Nasiru Akobe Suleiman
- Folayinka Ayofunke Ogunmuyiwa
- Ilochi Uchechukwu Nnaji

- Ibrahim Olajide Dada
- Fatima Ajuma Ojabo

Lagos University Teaching Hospital
- Muyiwa Rotimi*
- Adesoji Ademuyiwa
- Damilola Awotunde
- Chisom Agwu
- Oluwajuwon Afolayan
- Jennifer Okei
- Abidat Ashimi
- Oluwadarasimi Adeboyeku
- Abimbola Ogunbadejo
- Daniel Lawal
- Shakirat Adejumo
- Goodness Donye
- Tobiloba Lawal
- Gideon Osadare
- Titilola Awosika
- Jared Oseghale
- Danielle Obiwulu
- Christianah Otegbola
- Emmanuel Williams

Memfys Hospital
- Maduabuchi Paul Ufoegbunam*
- Chika A. Ndubuisi
- Friday G Okonna
- Obioma Richards Akwada
- Donald E. Ogolo

Olabisi Onabanjo University Teaching Hospital, Sagamu
- Fatungase Oluwabunmi Motunrayo*
- Shoyemi Ramotalai Oluwatoyin
- Shotayo Oluwakemi Adenike
- Adefuye Bolanle Olufunlola.
- Ogunjimi Luqman Opeoluwa
- Nwokoro Chigbundu Collins
- Ogundele Ibukunolu Olufemi
- Amosun Lukmon

University College Hospital, Ibadan (UCHI)
- Olusola Kayode Idowu*
- Babatunde B Osinaike
- Afolabi Adebayo Oladeji
- Oluwaseun Kehinde Adebayo
- Mutiu A Jimoh
- David A Aderinto
- Yemi Raheem Raji
- I Adeola Fowotade
- Ajibola Oladiran
- Oluwakemi Badejo
- Kehinde Abraham Ojifinni
- Adigun Tonitomi
- Mosimabale J. Balogun
- Taiwo A Lawal
- Oyeyemi E. Dada
- Yewande Olaoye Babalola
- Oluwasanmi Adekunle Ajagbe

- Olurotimi Olaolu Akinola
- Oluseun O. Saanu
- Olatunji Okikiola Lawal
- Olayinka Ramotu Eyelade
- Adegbolahan Jacob Fakoya
- Olukemi A Adekanmbi
- Mukaila O Akinwale
- Arinola Adeyoola Sanusi
- Foluke Oladele Sarimiye
- James Ayokunle Balogun
- Matias Ogbonia Orji
- Afieharo I. Michael
- Rukiyat Adeola Abdus-Salam
- Dare Isaac Olulana
- Omobolaji Oladayo Ayandipo
- Oludolapo Olawunmi Afuwape
- Sikiru Adekola Adebayo
- Tarela Frederick Sarimiye
- Oluseyi O. Agboola
- Thankgod C Okonkwo
- Augustine Oghenewveyin Takure
- Adekunle Daniel
- Douglas Efe

University Of Benin Teaching Hospital (UBTH)
- Akpabio Uforo Ezekiel
- Imuetinyan Rashida Edeki
- Aluya Eseosa Faith
- Ehiorobo Samson Edohen
- Omorogiuwa Idemudia Oduware
- Aighobahi George Akpede
- Peter Ikponmwosa Agbonrofo
- Michael Ediale
- Joel Enaholo
- Otasowie Osagie
- Oduware Emmanuel Ehigiegba
- Noruwa Patience Ekhator
- Omorogbe Scott Osahon
- Osaheni Osayomwanbo*

University Of Calabar Teaching Hospital (UCTH)
- Stella A. Eguma
- Sunday O. Sangolade
- Chinedu J. Anachunam
- Nkoyo E. Enyenihi
- Victoria E. Ndoma
- Augustine O. Odemwingie

University Of Ilorin Teaching Hospital
- Olanrewaju Olubukola Oyedepo
- Benjamin Olusomi Bolaji
- Christianah Iyabo Oyewopo
- Abdulrasheed Adegoke Nasir
- Olayide Sulaiman Agodirin
- Lookman Oluwatosin. Lawal
- Anne Oluwabunmi Mokuolu

University Of Maiduguri Teaching Hospital, Maiduguri (UMTH)
- Audu Idrisa*

- Ahmed Hamman Gabdo
- Nuhu Ali
- Jamila Audu Idrisa
- Mohammed A Ahmed
- Mohammed A S Abdullahi
- Ali Mohammed Ramat
- Ibrahim Musa Kida
- Babagana Bako
- Hassan M Dogo
- Babagana Usman
- Jibril Khalil
- Hamman Ibrahim Garandawa
- Sulaiman M Maina
- Olayinka Adewunmi
- Maryam Usman Kashim

University Of Port Harcourt Teaching Hospital (UPHTH)
- Job Otokwala*
- Vernatious Aniobi
- Busola Alagbe-Briggs
- John Udo
- Ageh Hannah

Somalia
National Leader: Mohamed Abdinor Omar

Madina Hospital
- Hilal Mohamed Nor*
- Abdishakor Mohamud Ahmed
- Mohamed Abdi Ahmed

Mogadishu Somalia - Turkey Recep Tayyıp Erdoğan Training and Research Hospital (TRTETRH)
- Abdullahi Said Hashi*
- Mohamed Sheikh Hassan
- Mohamed Farah Yusuf Mohamud
- Nasra Mohamud Hilowle
- Marian Muse Osman

Kalkaal Hospital
- Sakariye Abdullahi Hassan*
- Suleyman Abdullahi Mohamed
- Timothy Kimutai
- Abdullahi Mohamed Mohamud

Somaliland
National Leader: Hassan Ali Daoud, Mohamed Faisal

Borama Regional Hospital
- Mouna Ahmed Abdillahi*
- Fatima Eid Ibrahim

Burao General Hospital
- Omar Aden Yusuf*
- Mohamed Said Awil
- Hamse Awil Mohamoud

Hargeisa Group Hospital
- Ayoub Mohamed Suleiman

- Mohamoud Abdullahi Ismail*
- Samira Maxamed Macalin
- Abdirahman Abdirisak Ahmed

South Africa
National Leader: Juan Scribante

Provincial Leaders: Busiswe Mrara, Sade Hendricks, Nondwe Mgoqo, Jenny Nash, Fathima Paruk, Zainub Jooma, Zane Farina, Marie Oosthuizen, Estie Cloete

Addington Hospital
- Sebenzile Sikhakhane*
- Usha Singh
- Jayd Kanjee
- Nishen Gokal
- Nqobile Zulu
- Sabihah Murchie
- Mausum Beeput
- Nokukhanya Shange
- Sarisha Haripersad
- Leona Ravinath
- Kerissa Naidoo
- Thabang Kolanyane
- Njabulo Ntuli
- Lusanda Magwenyane
- Fezile Mkhize
- Thubelihle Nsele
- Lethuxolo Shange

Adelaide Hospital
- Angela Mary Thain Hartwig*
- Relebohile Joyce Khoabane
- Mfundo Gubhela

Butterworth Hospital
- Bongeka Mfecane*
- Musa Sarile
- Thobile Dlalisa
- Nashlen Naidoo

Charles Johnson Memorial Hospital
- Shepherd Nzenza*
- Jacob Myeni
- Kwanele P Majozi
- Mlungisi P Shange
- Zakhele Nxumalo
- Ziphokuhle Khumalo
- Mujinga Sylvie Biaya

Church of Scotland Hospital
- Siphelele Kubheka*
- Rodney Magwenya
- Thuba Mazibuko
- Nkanzimulo Dlamini
- Khayelihle Jobe
- Mondli Mfeka
- Lindokuhle Sangweni
- Snegugu Zondi

- Mbongeleni Zuma
- Siphesihle Njoko
- Mounir Raddadi
- Lindokuhle Zungu

Cloete Joubert Hospital
- Judy-Liesel Hardie*
- Marion Irene Frost
- Mandisa Sfundo Ziqubu

Cradock Provincial Hospital
- Elsje Bester*
- Anli van Niekerk
- Heinri Edwards
- Joy Awokiyesi
- Sinazo Ngini
- Carli Whitehead

Dr Pixley Ka Isaka Seme Memorial Hospital
- Tessa Korda*
- Theroshnie Kisten
- Halalisiwe Khanyi
- Shakeel Kader
- Matthew Jones
- N Buthelezi
- Chris Hooper
- Urekha Ballasur
- Susan Brown
- Alicia Ackerman
- Ahmad Asmal
- Muhammad Asmall
- Nico Hudson
- Rene Kruger
- Kedibone Mbanga
- Vuyisa Mdingi
- Molifi Moshoadiba
- Zanine Moyce
- Fezile Mthimkhulu
- Akhie Narain
- Marsha Ramburuth
- Tapiwa Sibanda
- Sivenderen Thaver
- Cherade Wilson
- Nondumiso Zondi
- Mkholisi Gama
- Genevie Borrageiro

Eerste River Hospital
- Martha Elizabeth Bronkhorst*
- Danielle Charlotte Hendricks

Emmaus Hospital
- Mampho Mochaoa*
- Yonela Tsewu
- Mbuso Nkala
- Lwanzo Mathe
- Deliwe Nkosi

General Justice Gizenga Mpanza Regional Hospital

- Roel Matos-Puig*
- Ernest Muragijeyesu
- Ria Devi Naidoo
- Nompumelelo Precious Sibiya
- Nthabiseng Precious Molokwane
- Shilendra Harripersad
- Dyavan Singh
- Vajra Chandrasekhar

Grey's Hospital
- Arisha Ramkillawan*
- Nomandla Tsibiyane
- Nandini Gramoney
- Thubelihle Jali
- Michelle Terry Dolores Smith
- Sinovuyo Madikane
- Ayanda Ntinga
- Sinenhlanhla Mkhize
- Zukiswa Cabangana
- Sakhisosenkosi Zamimpilo Hlela
- Parvania Munthree
- Takudzwa Shava
- Lieze Geldenhuys
- Dilshad Shaik
- Jithin Mohan
- Mihir Patel
- Sabeeha Khan
- Fathima-Zahra Rahiman
- Richard Tatenda Chikosi
- Alexandra Bench
- Marcel Simpson
- Natalie Hendricks
- Freda-Heléne Leuvennink
- Ingebor Jäger

Groote Schuur Hospital
- Rowan Duys*
- Margot Flint
- Simphiwe Gumede
- Abhilash Nair
- Aleta Sibi
- Aliya Bhorat
- Ayabonga Yedwa
- Chloe Ash
- Danika Govender
- Darren Piaray
- Dineo Kotu
- Jacob Blou
- Jani Gerber
- Jenna Piercy
- Johann Fourie
- Joseph Ruiz von Walter
- Joshua Louis
- Khelan R Dheda
- Khulile Singata
- Mahdiya Bhayat
- Mayilan Chetty
- Nabiha Ebrahim
- Naho Khorombi

- Nina Mabusela
- Olachi Emeruem
- Phi Nguyen
- Rahul Joseph
- Robyn Brown
- Samiya Bhorat
- Suvina Chanerika
- Tiashan Moodley
- Zaeem Ebrahim
- Zander Skye Isaac

Harry Gwana Hospital

- Mariette Grobbelaar*
- Dave Bishop
- Marzanne Nel
- Leesa Bishop
- Sanele Magagula
- Kirsten Marais
- Victor Ruhinda
- Suhail Sayad
- Nerlicia Sewnarain
- Aphiwe Mtshengu
- Tsakani Mabunda
- Sisanda Tiya
- Dipo Adeyemi
- Muhummed Uwais Moosa
- Lwazi Goso
- Anika Lucas
- Emma Scheepbouwer
- Nabeela Motala
- Glenda Watt
- Nok'zotha Njoko
- Gaby Nel
- Bonga Mthembu
- Melissa Purdon
- Previn Johnson
- Humeshan Naidoo
- Jan Kriel
- Howard Wain
- Buhlebonke Skhakhane
- Asheeq Soobader
- Abdur Razaaq Lamera
- Melanie Lesch
- Cara Dunn
- Hilde Turkstra
- Marcello Leita
- Tajil Manmohan
- Natanael Janse van Rensburg
- Nkosi Nxumalo
- Thandiwe Mchunu
- Jisheel Kanaye
- Tamsin Singh
- Bonga Khoza
- Melissa Yolanda Hippolite
- Nonhlanhla Zimase
- Nonhlanhla Madlala
- Stephanie Scriba

Isimelela Hospital

- Graeme Hofmeyer*
- Richard Southey
- Michaela Peters
- Muetu Kapena

King Edward Hospital
- Kasandri Govender*
- Alishka Naidoo
- Fiona Lourens
- Andisiwe Ngcobo
- David Govender
- Yasheen Maharaj
- Lwazi Mntungwa
- Dhivendra Singh
- Suman Mewa Kinoo
- Ruvashni Naidoo
- Kishan Anand Naidu
- Nhlakanipho Ngubane
- Maaneka Ramadhin
- Randolph Green-Thompson
- Nyameka Maluleke
- Karthik Naidoo
- Sne F Mkhize
- Patrick Kanana
- Brian Ubisi
- Mfundo Ndlela
- P Hiralal
- S Lakhani
- Balungile Dzingwe
- Andile Mfene
- Tumisho Bokgobelo
- Nithin Khoon Khoon
- Aadi Modi
- Chanel Dalais
- Zubair Omar
- Meegashen Narainsamy
- Nombuso Mlawu
- Saba Sivuyisiwe
- Taskeen Mather

Ladysmith Hospital
- Nozipho P. Mabaso-Langa*
- Brain Sibusiso Blakie Mbhele
- Sihlengiwe Zandisile Nyawose
- Jabulani Agrippa Nzimande
- Pholoso Prince Moele
- Mxolisi Brian Ndimande
- Nonhlanhla Zulu

Madadeni Hospital
- Marcin Kopieniak*
- Christian Kyanda-Kaboza
- Farrah Khan
- Etienne Venter
- Nondumiso Masondo
- Bonginkosi Nene
- Abiodun Lamina
- Nkosi Sibahle
- Samukelesiwe Kubheka

- Tilou Thobane
- Mzamo Mnguni

Madwaleni Hospital
- Andrew Miller*
- Steyn Botha
- Melandie Fourie
- Reinhard Schmidt
- Chris Westwood

Mahatma Gandhi Memorial Hospital
- Devandiran Harriraman Rungan*
- Devarani Naidoo
- Adushan Govender
- Zizipho Noluthando Madikizela
- Siddharth Mohan
- Bavna Hira
- Keshree Naidoo
- Valsura Ramsundar
- Brendan Moonsamy

Manguzi Hospital
- Mlibo Mthembu*
- Mark Blaylock
- Eddie Tembe

Mthatha Regional Hospital
- Busisiwe Cawe*
- Michael Ojonimi Ameh
- Osahon Daniel Erebor
- Sijabulile Cassius Sosibo
- Sikelela Siyibane
- Charles Choto
- Mlamli Dotye
- Zolelwa Zibuye Nandi
- Sanelisiwe Sivuyile Ngceba
- Thulisa Anela Qaziyana
- Chwayita Katshwa
- Unathi Simamkele Ntshongwana
- Sivuyise Gwazela
- Lerato Pakade
- Siphosihle Msutu
- Singleton Luxolo Sandla
- Thobeka Portia Ngcobo
- Sandiso Yekani
- Vuyolwethu Sotashe
- Khanya Galela

Nelson Mandela Academic Hospital
- Abongile Sukwana*
- Busisiwe Mrara (Provincial Leader)
- Lindubuhle Beba
- Mawande Mayibenye
- Samuel Alomatu
- Siqhamo Magadla
- Gloria Hyera
- Siphesihle Khoza
- Lelethu Jwambi
- Mzomhle Kiza

- Athandiwe Qhonono
- Lungisa Petse
- Nomvuzo Saqu
- Lwandile Mtshabe
- Sibi Joseph
- Yondela Sidoyi
- Banele Kokose
- Anele Dayimani
- Chwayita Makrexeni
- Emma Muendo Loko
- Apelele Futshane
- Sinesipho Ndedwa
- Nosiphiwo Semane
- Aphiwe Mqhayi

New Somerset Hospital
- Jacob Blou*
- Rahul Joseph
- Joseph Ruiz von Walter
- Phi Nguyen
- Joshua Louis
- Chris Pearce
- Robin Veitch
- Samiya Bhorat
- Aliya Bhorat
- Dineo Kotu
- Margot Flint
- Nina Mabusela

Newcastle Hospital
- Sandile Dube*
- Makura Ndhlovu
- Siphiwe Ngema
- Kaajal Brijlall
- Thabani Nyathikazi
- Kashal Ramsaroop
- Ameer Beeharry

Ngwelezana Hospital
- Stefan van der Walt*
- Chiara Anne Baars
- Karishma Heeramun
- Sabrina Pillay
- Xander Botha
- Christopher Brits
- Chad Young
- Andrew Monahan
- Mikaeel Abdool
- Brendon Chetty
- Anele Thabiso Ndlangisa
- Asanda Marawu
- Phumzile Khuboni
- Philiswa Nkondlo
- Siphe Xabendlini
- Abu Bakr Arbee
- Michael Robert Cooke
- Hsin-Nua Wang
- Jeandré Malherbe
- Craig Sydney Scholtz

- Michael Santana
- Jo Shadwell
- Julia Tooke
- Paul Martin Pretorius
- Zamancwango Mncwango
- Luke Miguel Fourie
- Daniel McElhenny
- Riza Ehlers
- Andile Twele
- Julia Kenmuir
- Rory Livanos
- Gregory Wiid
- Lwande Gongota
- Anele Thabiso
- Zaza Shange

Northdale Hospital

- Dela Maiwald*
- Henri van der Merwe
- Reena Panicker
- Saidur Molla
- Sarel Kruger
- Sanam Chuturgoon
- Laila Choonara
- Werner Erasmus
- Aashiqui Ramith
- Alana Williams
- Anathi Sokanyile
- Dane Rampini
- Emma Howes
- Gabriella Kwant
- Heilisha Dehaloo
- Kaylin Adams
- Seelo Mvelase
- Mthokozisi Nsele
- Ridwa Hajee
- Saahil Bhanial
- Tajil Manmohan
- Sajit Alladeen

Port Shepstone Hospital

- Robert Stevenson*
- Preston Moodley
- Shirish Sewpersad
- Spheh Ndlovu
- Marli Ellis

Prince Mshiyeni Memorial Hospital

- Rajesh Ramjee*
- Myint Aung
- Prashant Gokal
- Omishka Hirachund
- Sizwe Zungu
- Allison Smith
- Wayne Rees
- Nishan Pillay
- A Hirjee
- Khaya Matanzima
- Pavan Kishendutt

Queen Nandi Hospital
- Ravi Mishra*
- Yoshua Bwambale
- Mphathiseni Dlamini
- Dedi Mutondo
- Mbayo Yuma
- Chane Retief
- Wandile Shandu
- Precious Mzobe
- Tando Mpotulo
- Emily du Plessis

Robert Mangaliso Sobukwe Hospital
- Judith Maria Oosthuizen*
- Nicquin Nervin Adams
- Andrea Snyman
- Sunnet Ellis
- Jacobus Rademan
- Lisa Combrink
- Herman Hendrik van der Linden
- Pieter Gerhardus Marais
- Matshidisho Nodoba
- Thazriq Eksteen
- Yusuf Ameen
- Sidharth Singh
- Tamilla Nieuwoudt
- Michaela Bredenkamp
- Nitesh Naranbhai
- Anke van der Linden

St Mary's Hospital
- Adam Asghar*
- Nazreen Ahmed
- Lungile Mthethwa
- Zein Elabideen Darwish
- Aavishkar Rooplall
- Kamintha Govender
- Kershan Munien
- Mohamed Nadeem Ali
- Zahraa Vahed
- Thembelihle Pretty Hloni
- Siyabonga Ximba
- Phumelele Ntinga
- Leeyen Singh
- Sunhera Sukdeo
- Jabulilie Solomon
- Nikeziwe Ncwane
- Nokwanda Mabaso
- Pumla Zondi

Steve Biko Academic Hospital
- Nhlakanipho Shabalala*
- Tiffany Pratt
- Mmapali Mokapela
- Chuene Hlahla
- Nkateko Chauke
- Petrus Jansen van Vuuren
- Chloe Howell

- Sanjula Pillay
- Dineo Mashigwane
- Fred Mulunda

Uitenhage Provincial Hospital
- Jannes Moolman*
- André Oosthuizen
- Allison Muller
- Maryll Stuurman

Victoria Hospital
- Zahnne Fullerton*
- Ziyaad Limalia
- Abigail Davies
- Kelsey Bester
- Margot Flint
- Simphiwe Gumede

Wentworth Hospital
- Mergan Naidoo*
- Dylan Barnard
- Jadon Haridutt
- Leigh Coetzee
- Shanaaz Fortune
- Deveena Jasmin Maharaj
- Muhammad Yusuf Sayed Essop

Zithulele Hospital
- Nicholas Fine*

Sudan
National Leader: Tarig Fadalla, Alshaima Koko

Merowe Daman Hospital
- Mazin Mahir*
- Alhussein Hamid
- Abdelfatah Abdelmageed

Wadi-Halfa Hospital
- Sara Abdalla*
- Shadan Elsayied
- Moaj Ahmed
- Alamin Ali

Tanzania
National Leader: Tim Baker, Karima Khalid, Bernard Mbwele

Muhimbili University of Health and Allied Sciences
- Aneth C Kaliza
- Linda Mlunde
- Elibariki Mkumbo
- Rafael S Shayo
- Godfrey Barabona
- Happines Biyengo
- Alma O Damasy
- Anna Hvarfner
- Sabra Hussein

- Anab F Issa
- Charles Machumu

Bahi District Hospital
- Edson Gaston Luvakubusa*
- Atupyane Mati
- Simforosa A. Atanas
- Monica N. Lucas
- Michael J. Munuo
- Zulfa S. Matola

Chemba District Hospital
- Greyson Victor Joseph*
- Asadulillah Kidusi
- Scholastica B. Bitesigirwe
- Dionisia A. Mkonga

Ilsofu District Hospital Sumbawanga
- Jenitha Joseph Cheru*
- Petronela Malema
- Shija Mathias

Kilimanjaro Christian Medical Center
- Maynard Samuel Makere*
- Chrystal Tarah Munyanyi
- Kelvin Fidelis Rutahoile
- Linnah Abraham Njau
- Rutendo Gina Mutimukulu
- Precious Nicole Gunha

Mawenzi Regional Refferal Hospital
- Leonard Joseph Mrosso*
- Serena Silayo
- Flora Mbuyu
- Joyce Materu
- Mary John Chuwa

Mbalizi Designated Regional Hospital
- Mathayo Thomas*
- Grace Charles
- Dina J. Lihangaka
- Herieth Mwenda
- Bebatus Nyamahanga

Mbeya Regional Referral Hospital
- Cholela Braison Augustino*
- Pascal Robert Dalama*
- Remigius Richard Apolinary
- Edward Mathias Nsemwa
- Magreth Gitige Mangi
- Catherine Gatwa
- Geofrey Oscary Kayombo
- Winfrida Yena Ndebele
- Keneth Mashauri Kyando
- Stella Maneno Jacob

Mbeya Zonal Referral Hospital (MZRH)
- Sixtus Ruyumbu Safari*

- Filbert Francis Ilaza

Muhimbili Orthopedic Institute
- Shafi Hamis*
- Monica Peter
- Rehema Mushi
- Sajda Ally
- Heneriko Benedicto
- Gustavu Mbunda
- Reuben Andeshi
- Abel Elias
- Willium Anstone
- Fransesco Aron
- Saguda Matondo
- Suzana Constantine

Mwalimu Nyerere Regional Hospital
- Ezekiel Komanya*
- Samwel Asantaeli
- Francis Joseph Gwejo
- James Gambaseni

Mwananyamara
- Hubert August Ngowi*
- Lucky Harran Mkocha
- Sesilia Michael
- Onesmo Seth Laizer
- Dede Dotto Chapajuja

Rubya Catholic District Hospital
- Arserius Rutaiwa*
- Jackline P. Kokuhirwa
- Efgenia M. Bombo
- Domina J. Muikila
- Leah D. Mwelinde

St. Joseph Designated District Hospital
- Deusdedith William*
- Liberatus Mushi
- Bonphace B. Msimbano
- Clement J. Mchomvu

Sumve Designated District Hospital
- Charles Charles Petro*
- Pendo N. Yombo
- Tekla Timotheo
- Christian L. Kusekwa
- Servat Ikigijo
- Patricia Osward

Utete Hospital Rufiji
- Ephrem Tahhani
- Stamili Mkumba
- Senso William
- Hadija Wanguvu

Uwata Hospital
- Edwin Edward Ernest*

- Edwin Edward
- Eliab J. Daud
- Innocent B. Sanga
- Anna J. Kalinga

Tunisia
National Leader: Nahla Kechiche

Habib Bourguiba University Hospital
- Rania Ammar*
- Mounir Bouaziz
- Fatma Kolsi
- Rahma Daoued
- Mouna Jerbi
- Noureddine Rekik
- Amel Bouzid

Hedi Chaker Hospital of Sfax
- Hayet Zitouni*
- Sahar Elleuch
- Salma Toumi
- Fatma Khanfir
- Sana Omri
- Kamel Kolsi
- Mohamed Ben Hmida
- Moamed Maalej
- Kais Chaabane
- Riadh Mhiri

Hospital of Gabes
- Samar Bellil*
- Houda Belmabrouk

University Hospital of Mahdia
- Oussama Jaoued*
- Imen Bannour*

University Hospital of Monastir
- Sawsen Chakroun
- Nessrine Ben Saad
- Marwen Baccar
- Lassaad Sahnoun

Uganda
National Leaders: Adam Hewitt-Smith, Arthur Kwizera

Budaka HCIV
- John Kiboma Wogabaga*
- Mercy Logose
- Yaya Miriam Jackline

Busiu HCIV
- Roggers Odongo *
- Aruho Moses

Busolwe Regional Hospital
- Innocent Musiime*

- Joan Naluyima
- Peter Magala

Buwasa HCIV
- Moses Tommy Wobudubire*
- Mary Nambuba
- Gerald Waniala
- Joram Esemu
- Getrude Nakiria
- Emily Chesang
- Zamu Logose
- Salim Nabila

Hoima Regional Hospital
- Sophie Namasopo*
- John Kalungi
- Isooba Safiyu Ayub
- Amon Asindu
- Elizabeth Namuyala
- Isaac Muyanja
- Ivan Mwebesa

Jinja RRH
- Ruth Muhindo*
- Anthorny Wasukira
- Mary Innocent Waswa
- Ronald Aruho
- Moses Mwebaza Kakooza
- Grace Nambuya
- Brenda Balungi

Katakwi General Hospital
- Joseph Emuron*
- Clare Ameri
- Roggers Kedi
- Constance Amulen

Lubaga Hospital
- Gladys Nabukenya*

Mbale RRH
- John Paul Ochieng*
- Joan Nachuka
- Priscilla Mercy Adikin
- Peace Draleru
- Naume Etoko Akello
- Federeth Nabisubi
- Hasifah Namutebi
- Glades Isina
- Jennifer Kipwola
- Derrick Muhwana
- Fred Salya
- Harriet Patricia Asekenye
- Christine Tino
- Doreck Nahurira
- Assen Kamwesigye
- Jude Mulowoza
- Ronald Aridriga
- Sam Orech

- Brenda Namugga
- Richard Gamubaka
- Fred Maiso
- Joshua Orikiriza
- Andrew Lemu
- Jena Amos Chebet
- Moses Chelangat
- Bruno Onen Chan
- Zainab Kabasemeza

Mulago National Referral Hospital
- Jane Nakibuuka*
- Paul Omagor
- Lynn Martha Nattabi
- Vanessa Nantale Lubulwa
- Benard Oyang
- Moses Arinaitwe
- Eddy Cantong
- Vanessa Nanono
- Dramanigo Kodjo
- Maria Assiimwe
- Bright Nagaba
- Ceasor Julius Owor
- Nicholas Kabugo
- Emmanuel Eemu
- Dickson Kamoga
- Charles Emuduko
- Sheif Semakula
- Cornelius Sendagire

Nsambya Hospital
- Tomanya Kakuru Kenneth*
- Susan Nabunya
- Joyce Wamala N.
- Claire Namuwaya
- Chelsea Edwards Sanyu

St Mary's Hospital Lacor
- Kenneth Innocent Nyeko*
- Mwesigwa Lukwago Seezi
- Allan Phillip Barigye
- Herman Tabula Mpumbu
- Derrick Mukurasi
- Ronnie Omoro
- Maurine Lenia

Uganda Heart Institute
- Catherine Namutebi*
- Sarah Namatovu

Zimbabwe
National Leader: Pisirai Ndarukwa, Newten Handireketi

Marondera Hospital
- Ndaiziwei Masukhume*
- Samson Pomo

Supplementary Material S1. Ethical approval processes for the African Critical Illness Outcomes Study

Country	National ethics committee approval	National ethics number	University ethics approvals	Hospital ethics approvals
Botswana				1
Burkina Faso	Ministerie de la Sante et de L'Hygiene Publique	No number. 2 August 2023		1
Congo	Ministerie l'Enseignement Superieure de la Recherche Scientifique et de l'Innovation Technologique	053-40/MESRIT/DGRST/CERSSA/-23		
DRC			1	
Egypt			1	2
Ethiopia	AHRI/ALERT	PQ49/23		
Gambia				1
Ghana			1	2
Lesotho	Ministry of Health	Ref: ID 211-2023		
Libya	Ministry of Health	NBC:002.H-23.10		
Morocco				1
Mozambique				1
Namibia	Ministry of Health and Social Services	Ref: 22/3/1/1		
Nigeria	National Health Research Committee of Nigeria	NHREC/01/01/2007-10/08/2023	1	14
Somalia	Ministry of Health and Human Services	Ref/MOHHS/DGO/0697/Sep2023		
Somaliland	Ministry of Health Development	Ref: MOHD/VM:3		
South Africa	South African National Clinical Trial Registry	DOH-27-072023-9030	3	33
Sudan				2
Tanzania	National Institute for Medical Research	NIMR/HQ/R.8a/Vol.IX/4473		
Tunisia			1	4
Uganda				1
Zimbabwe	Medical Research Council of Zimbabwe	MRCZ/E/346		

Supplementary Material S2. Broadcasting document

ACIOS African Critical Illness Outcomes Study — APORG African Perioperative Research Group — APPRISE African Partnership for Perioperative and Critical Care Research

IMPORTANT PATIENT INFORMATION

A research study is being conducted at …………………..Hospital.

The research study is being done by Dr ………. from the Department of ………..

Why is this research study being done?

To understand how sick patients are in hospital.

Why are we telling you about this research study?

All patients in this hospital are part of the research study. It is a requirement that some details pertaining to your clinical care are entered into a research study folder. Information from this folder will be used anonymously to understand how sick patients are in hospital, and what we might be able to do to improve the care for sick patients.

Will this research study affect my care while I am in hospital?

No. You will still receive the same care while you are in hospital.

Will my name or any personal details be kept by this research study?

No. Your name and personal details will not be kept as part of this research study. All information from the notes will be kept strictly confidential.

Are there any risks or benefits associated with this project?

No. There are no risks or direct benefits associated with this research study.

Who should I contact if I have any questions or concerns?

Please contact Dr …………….. on telephone……………….

If you have questions about your rights or welfare as a participant, please contact the UCT Faculty of Health Sciences Human Research Ethics Committee on +27 (0)21 406 6338.

ACIOS Study Broadcasting signage

Supplementary Material S3. Hospital data record form

African Critical Illness Outcomes Study (ACIOS) – Hospital CRF

Section 1: Hospital characteristics

1. Language preference: ☐ English ☐ French ☐ Arabic ☐ Portuguese ☐ Other ……………………….
2. Hospital name: ………………………………………………………
3. Country: ………………………………………………………
4. Level of hospital:

   ☐ First-level (e.g. district) ☐ Second-level (e.g. Regional) ☐ Third-level (e.g. University/Central/National)

5. Type of hospital: ☐ Government ☐ Private ☐ Charitable
6. Total number of hospital beds: Total ☐☐☐☐
7. Number of beds in High Care Units: Total ☐☐☐☐
8. Number of beds in ICUs: Total ☐☐☐☐
9. Population served (catchment) of the hospital: ☐☐☐☐☐☐☐

Section 2: Available Resources

Are the following available in your hospital?

	EQUIPMENT	Always	Sometimes	Never	Don’t know
1	Clock with second hand	○	○	○	○
2	Pulse oximeter & probe	○	○	○	○
3	Blood pressure measuring equipment (eg. sphygmomanometer with a stethoscope)	○	○	○	○
4	Blood pressure cuffs of different paediatric and adult sizes	○	○	○	○
5	Light source (lamp or flashlight)	○	○	○	○
6	Thermometer	○	○	○	○
7	Suction machine (electric or manual)	○	○	○	○
8	Oxygen supply 24h/day (cylinder, concentrator (with electricity supply) or piped oxygen)	○	○	○	○
9	Flow meter (if using cylinder or piped oxygen)	○	○	○	○
10	Leak-free connectors from oxygen source to tubing	○	○	○	○
11	Bag Valve Mask (resuscitator) – neonatal, paediatric and adult sizes	○	○	○	○
12	Sharps disposal container	○	○	○	○
13	External heat source	○	○	○	○

	CONSUMABLES	Always	Sometimes	Never	Don’t know
14	Soap or hand disinfectant	○	○	○	○
15	Examination gloves	○	○	○	○
16	Suction catheters of paediatric and adult sizes	○	○	○	○
17	Guedel airways of paediatric and adult sizes	○	○	○	○
18	Pillows	○	○	○	○
19	Oxygen tubing	○	○	○	○
20	Oxygen nasal prongs	○	○	○	○
21	Oxygen face masks of paediatric and adult sizes	○	○	○	○
22	Oxygen face masks with reservoir bags of paediatric and adult sizes	○	○	○	○
23	Masks for Bag Valve Mask (resuscitator) – neonatal, paediatric and adult sizes	○	○	○	○
24	Compression bandages	○	○	○	○
25	Plasters or tape	○	○	○	○
26	Gauze	○	○	○	○
27	Intravenous cannulas of paediatric and adult sizes	○	○	○	○
28	Intravenous giving sets	○	○	○	○
29	Skin disinfectant for cannulation	○	○	○	○
30	Syringes	○	○	○	○
31	Nutrition	○	○	○	○
32	Nasogastric tubes	○	○	○	○
33	Lubricant for nasogastric tube insertion	○	○	○	○
34	Intramuscular needles	○	○	○	○
35	Intraosseous cannulas of different sizes	○	○	○	○
36	Blankets	○	○	○	○
37	Facemasks for Infection Prevention and Control	○	○	○	○
38	Aprons or gowns	○	○	○	○
39	Charts/notes for documentation	○	○	○	○
40	Pens	○	○	○	○

	DRUGS	Always	Sometimes	Never	Don’t know
41	Oral rehydration solution	○	○	○	○

42	Intravenous crystalloid fluids (eg. normal saline or Ringer’s Lactate)	○	○	○	○
43	Intravenous dextrose fluid (eg. 5%, 10% or 50%)	○	○	○	○
44	Oxytocin	○	○	○	○
45	Adrenaline	○	○	○	○
46	Appropriate antibiotics	○	○	○	○
47	Diazepam	○	○	○	○
48	Magnesium sulphate	○	○	○	○
49	Paracetamol	○	○	○	○
50	Local anaesthetic (eg. 2% lignocaine) (eg. for intraosseous cannulation)	○	○	○	○

	**HR**	Always	Sometimes	Never	Don’ t know
51	Health workers (eg nurses) with the ability to identify critical illness 24h/day	○	○	○	○
52	Health workers with the (eg nurses) ability to care for critically ill patients 24hrs/day	○	○	○	○
53	Senior health worker (eg doctor) who can be called to assist with the care of critically ill patients 24hrs/day	○	○	○	○

	**TRAINING**	Always	Sometimes	Never	Don’ t know
54	The health workers are trained in the identification of critical illness	○	○	○	○
55	The health workers are trained in the care of critically ill patients	○	○	○	○

	**ROUTINES**	Always	Sometimes	Never	Don’ t know
56	The hospital has well-defined routines for the identification of critical illness	○	○	○	○
57	The hospital has well-defined routines for managing critically ill patients	○	○	○	○
58	There is a routine for the provision of EECC without taking into account patients’ ability to pay	○	○	○	○
59	There are routines for who and how to call to seek senior help 24hrs/day, 7 days/week	○	○	○	○
60	There are routines for integrating EECC with other care including the definitive care of the underlying condition (eg. use of condition-specific guidelines)	○	○	○	○

	**GUIDELINES**	Always	Sometimes	Never	Don’ t know
61	There are written guidelines for the identification of critical illness	○	○	○	○
62	There are written guidelines for the essential care of critically ill patients	○	○	○	○

	INFRASTRUCTURE	Always	Sometimes	Never	Don’ t know
63	Designated triage area (area for the identification of critical illness) in the Out-Patient Department or Emergency Unit (area of the hospital where patients arrive)	○	○	○	○
64	Running water	○	○	○	○
65	Designated space for the care of critically ill patients (eg. a bay, ward, high care unit)	○	○	○	○
66	Areas for separating and managing patients with a suspected or confirmed contagious disease from those without	○	○	○	○

Supplementary Material S4. Case record form

ACIOS African Critical Illness Outcomes Study — APORG AFRICAN PERIOPERATIVE RESEARCH GROUP — APPRISE African Partnership for Perioperative and CRitical Care ReSEarch

African Critical Illness Outcomes Study (ACIOS) – Patient CRF

Clinical assessment

Date of assessment: d d / m m / y y y y

Vital signs done by the investigating team: ☐ Y ☐ N   If no, specify ☐ Vital signs from patient records ☐ Not done

Position of patient:

☐ Lying flat on back(<30°) ☐ Lying on side ☐ Head-up(30°-60°) ☐ Sitting(>60°) ☐ Head-down ☐ Other

Airway patency: ☐ Normal ☐ Partial obstruction ☐ Complete obstruction

Conscious level (AVPU): ☐ Alert ☐ Responds to Voice ☐ Responds to Pain ☐ Unresponsive

Heart rate ☐☐☐ /min   Oxygen saturation ☐☐ %   Respiratory rate ☐☐ /min

Systolic blood pressure ☐☐☐ /mmHg   Diastolic blood pressure ☐☐☐ /mmHg

Currently receiving IV fluids ☐ Y ☐ N   Receiving oxygen (now) ☐ Y ☐ N

Receiving vasopressor/inotrope (now) ☐ Y ☐ N   Airway action (now) ☐ Y ☐ N

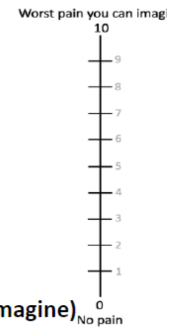

Circle the worst pain you had in the last 24 hours: 0 (no pain) 1 2 3 4 5 6 7 8 9 10 (worst pain you can imagine)

Other information

Date of hospital admission: d / m m / y y y y   Urgency of admission: ☐ Emergency/acute ☐ Elective

Age ☐☐☐ years   Sex ☐ M ☐ F

Ward type: ☐ Medical ☐ Surgical ☐ Maternal ☐ Other

Ward level: ☐ General ward ☐ High care unit/HDU ☐ ICU

Main category for admission: ☐ Non-communicable disease ☐ Maternal health ☐ Trauma ☐ Infection

Known chronic disease or pregnancy (tick all that apply):

☐ Pregnant ☐ Hypertension ☐ Diabetes ☐ Cancer ☐ COPD / Asthma

☐ Heart Disease ☐ HIV / AIDS ☐ Tuberculosis ☐ Other ☐ None

Any surgery during this admission: ☐ Y ☐ N   Treatment limitations (e.g. not for resuscitation): ☐ Y ☐ N

Follow-up

Total days in hospital from admission to 1. discharge/ 2. day 7 after clinical assessment (whichever occurs first) ☐☐

Status at 7th day in-hospital after clinical assessment: ☐ Discharged Alive ☐ Alive still in-hospital ☐ Died

Date of discharge or death up to and including day 7 after clinical assessment d d / m m / y y y y

ACIOS unique patient ID ☐☐☐ ☐☐☐ ☐☐☐☐

✂---

Patient name: ______________________   DOB d d / m m / y y y y

Patient hospital number______________________   Ward ______________ Bed______________

Supplementary Material S5. African Critical Illness Outcomes Study (ACIOS) definitions

Definitions:

Position of patient: The position the patient is in the bed/chair when the investigating team arrive at the bedside. The number of degrees refers to the angle of the head and body compared to the legs.

Airway patency: Normal is an unobstructed airway. Partial obstruction may be indicated by stridor, secretions in the airway identified by gurgling, or snoring. Complete obstruction is evident by a see-saw chest movement (chest down and abdomen up with attempted breathing against a closed glottis). Complete obstruction is an airway emergency and requires calling the attending clinical team immediately.

Conscious level AVPU: Measurement of conscious level. Is the patient Alert = A. If they are not alert but they respond to your voice = V. If they don't respond to voice but respond to a painful stimulus = P. If they remain unresponsive even with a painful stimulus = U (unresponsive).

Currently receiving IV fluids: At the time of clinical assessment, the patient is receiving IV fluids if IV fluids are hanging at the bedside, and currently dripping into an intravenous cannula, or the patient has been receiving fluids as described within the previous few minutes but is now finished and a new fluid is being prepared to be administered.

Receiving oxygen (now): At the time of clinical assessment, the patient is receiving oxygen if supplementary oxygen is currently flowing into a nasal cannula, face mask or other delivery device that is correctly fitted so that oxygen is entering the patient's lungs.

Receiving vasopressor/inotrope (now): Ongoing care with a vasopressor or inotrope infusion – for example noradrenaline, adrenaline, dopamine or dobutamine.

Airway action (now): An action to open the airway or maintain a free airway. For example: chin lift, jaw thrust, oro-pharyngeal airway, naso-pharyngeal airway, intubated patient.

Receiving Essential Emergency and Critical Care (EECC): Patients will be deemed to be receiving EECC if they are:

- critically ill due to the conscious level criterion and:
  - are lying in the lateral position or
  - have an oro-pharyngeal or naso-pharyngeal airway inserted in their pharynx or
  - have an ongoing chin-life or jaw-thrust or
  - have other airway protection.
- critically ill due to a respiratory criterion and:
  - are receiving oxygen.
- critically ill due to a circulatory criterion and:
  - are receiving intravenous fluids or
  - are receiving a vasopressor or inotrope.

High care unit/HDU: A unit or ward or part of a ward which is dedicated to providing an increased level of care when compared to a general ward. High care units often have increased nurse:patient ratios, more equipment and more advanced care such as oxygen, CPAP, vasopressors etc. This does *not* include units with mechanical ventilation, as that is an ICU. Includes recovery rooms providing an increased level of care.

ICU: A unit or ward which is dedicated to providing an increased level of care when compared to a general ward or high care unit including mechanical ventilation.

Main category for admission: The main diagnosis or reason that the patient is being treated in hospital.

Treatment limitations: A patient has a treatment limitation if the clinical team have made the clinical judgement that some treatments would not be in the patient's best interest. For example "DNR" (do not resuscitate in the event of a cardiac arrest), or "Not for ICU" in the event of deterioration.

Days in hospital: Total number of days in hospital from admission.

Status at hospital discharge or 7th day in-hospital after clinical assessment: The survival status of the patient at hospital discharge, or at the 7th day after clinical assessment (if the patient had not yet been discharged). The study is censored at the 7th day after clinical assessment.

Supplementary Material S6. The African Critical Illness Outcomes Study (ACIOS) Statistical Analysis Plan

The African Critical Illness Outcomes Study (ACIOS)

A prospective, multi-country, multi-centre, observational study to determine the hospital point-prevalence and mortality rates of adult patients with critical illness in acute hospitals in Africa.

Statistical Analysis Plan (SAP)

Version 1.0
Date: 13/2/2024

Registration ClinicalTrials.gov - NCT06051526
Based on "ACIOS protocol version 1.0 HREC approved"

**Persons contributing to the analysis plan**	
**Names and positions**	Assoc Professor Tim Baker, MUHAS<br>Professor Bruce Biccard, UCT<br>Professor Rupert Pearse, QMUL<br>Dr Carl Otto Schell, KI<br>Anneli Hardy (Statistician)
**Authorisation**	
**Position**	**Chief Investigator**
**Name**	Assoc Professor Tim Baker, MUHAS
**Signature**	
**Date**	13th February 2024
**Position**	**Trial Statistician**
**Name**	Anneli Hardy
**Signature**	
**Date**	2024-02-13

Remit of the SAP
The purpose of this document is to provide details of the statistical analyses and presentation of results to be reported within the principal paper of the ACIOS study. It is important to set these out and to agree them in advance of inspecting the outcome data for the study, so that data derived decisions in the analysis are avoided. Any exploratory, post hoc, or unplanned analysis will be clearly identified as such in the study analysis report.

Timing of the SAP
The SAP version 1.0 was written prior to the investigators having access to the data.

1. Study Summary

**Short title**	ACIOS
**Methodology**	A prospective, international, multi-centre, observational study.
**Research sites**	Acute hospitals in African countries.
**Objective**	To determine the hospital point-prevalence, and mortality rates of adult patients with critical illness in hospitals in Africa.
**Number of patients**	Not specified. All eligible patients in participating hospitals.
**Inclusion criteria**	All in-hospital patients aged 18 years or older in all departments and wards in participating hospitals in Africa.
**Exclusion criteria**	None
**Patient follow-up**	Until hospital discharge or death, censored at 7 days after inclusion.
**Primary outcomes**	1. The presence of critical illness<br>2. 7-day in-hospital mortality
**Data collection duration**	One day in each hospital in September-December 2023 plus 7 days follow-up in each hospital
**Proposed start date**	7th September 2023
**Proposed end date**	27th December 2023

2. Introduction

Critically ill patients – those in *a state of ill health with vital organ dysfunction, a high risk of imminent death if care is not provided, and the potential for reversibility*.[1] – have particular needs, and managing these needs is a core function of hospitals. Triage at admission and on the wards is needed to identify these patients with critical illness.[2,3] Critically ill patients need regular contact with health workers and close observation and frequent modifications to care, either in general wards, or in specialised locations such as Intensive Care Units (ICUs) and High Dependency Units (HDUs).[4] Rapid Response Teams of acute care specialists may be implemented in hospitals to provide care when called by ward staff.[5]

There are reports of gaps in the readiness and provision of critical care in hospitals in Africa.[6-8] Essential Emergency and Critical Care (EECC) has been developed and defined as the first-line care that should be provided to all critically ill patients.[9,10] Focusing on the first-line care in EECC is a strategy to address the gap in critical care. In our previous work an unmet need of EECC of 50-90% was found in hospitals in Malawi,[8] and there have been many calls to increase the coverage of EECC to address this gap.[9,11-13]

While it is accepted that critical illness and the underlying causes of critical illness are common, the number of patients with critical illness has not been accurately quantified.[14-16] In one region of Sweden we found 10.5% of hospital inpatients to be critically ill,[17] and in a Tanzanian university hospital's emergency unit, 10.7% of patients were critically ill at arrival.[18] Global estimates have been attempted by using the admission rates to ICUs but this method reflects national and local uses of ICUs which vary greatly even between high-income countries.[19] The indirect annual global estimate of 30-45 million adults made by extrapolating the incidence of common diseases leading to critical illness in North America is likely to be an underestimate as the burden of disease is greater in settings of lower resources.[20] Moreover, the mortality of critically ill patients has not been accurately quantified, with reports of 18-82% in-hospital mortality rates.[21-25]

A patient's vital signs (heart rate, respiratory rate, blood pressure, conscious level, body temperature, oxygen saturation) are commonly used measurements in hospital care. Deranged vital signs have been shown to correlate with negative outcomes such as admission to the ICU,[26,27] unexpected cardiac arrest,[27,28] and mortality,[27,29] and are pragmatic and useful as criteria for the identification of critical illness.[30,31]

This prospective, international, multi-centre, observational study of all adult in-patients in hospitals across Africa, is based on the methods we developed in the International Surgical Outcomes Study (ISOS),[32] European Surgical Outcomes Study (EuSOS),[33] African Surgical Outcomes Study (ASOS),[34] and African COVID-19 Critical Care Outcomes Study (ACCCOS)[22] studies. Using vital-signs based criteria, we will determine the hospital point-prevalence of critical illness. We will collect data on the care provided to patients, so to determine the coverage of essential emergency and critical care. We will follow the adult in-hospital patients for 7 days or until hospital discharge (whichever is sooner), allowing an estimate of the mortality rate and patients at increased risk who are critically ill in this population. The knowledge generated in the ACIOS study will assist in improving organisation of acute hospital services with the goal of averting substantial numbers of preventable deaths in African hospitals.

3. Statistical Analysis Plan

3.1 General analysis principles

Data will be presented at a continental African level. All institutional and national level data will be anonymised prior to publication. Categorical variables will be described as proportions and will be compared using chi-square tests. Continuous variables will be described as mean and standard deviation if normally distributed or median and inter-quartile range (IQR) if not normally distributed. No comparisons between groups will be performed at a univariate level.
For the analysis of the objectives, we will present the following information:

- The number of patients included in each analysis.
- Summary statistics of the outcome (e.g. median (IQR), mean (SD), number (%), range).
- A point estimate, odds ratio or hazard ratio with 95% confidence intervals.
- A two-sided p-value with a significance level of <0.05 will be used where relevant.

For data that are not necessary for each objective, imputation of missing observations will not be made and will be reported descriptively. For data necessary for each objective – see sections 3.4 to 3.8 below.

Statistical analyses will be performed using the Statistical Package for the Social Sciences (SPSS) version 28.0.1.1 (SPSS Inc., Chicago, IL, USA).

3.2 Sample Size / Recruitment

As many sites as possible will be recruited in participating countries. All adult patients will be eligible for inclusion in the sites. A sensitivity analysis will be done for each objective including only data from hospitals that recruited >90% of eligible patients. We do not have a specific sample size and statistical models will be adapted to the event rates provided by the sample recruited. Participation in the study, and completeness of follow-up will be illustrated by a STROBE flow diagram.

Patient recruitment and description will be presented as follows:

- STROBE flow diagram including i) countries, ii) number of eligible patients, iii) patients included and excluded.
- The number of participating hospitals, hospital characteristics and patients at each hospital level will be reported in a table. Detailed hospital characteristics will be provided in a Supplementary Table.
- The patient characteristics of the cohort will be presented in the table described in Section 3.4 below.

3.3 Objectives

1. To establish the proportion of adult (18 years or older) inpatients in African hospitals that are critically ill.
2. To establish the mortality rate of the critically ill patients and those who are not critically ill.
3. To estimate the proportion of critically ill patients who receive EECC.
4. To investigate the association between the provision of EECC to critically ill patients and mortality.
5. To determine the availability of resources for EECC in African hospitals.

3.4 Statistical analysis plan for Objective 1 "*proportion of patients with critical illness*"

We will present the number and proportion of included patients who have critical illness, where critical illness is defined using the severely deranged vital sign criteria specified in the protocol.

We will present a breakdown of data by vital sign derangement, main category of admission (NCD, maternal, trauma, infection), by ward type (medical, surgical, maternal, other), by ward

level (general ward, HDU, ICU), by urgency of admission (emergency, elective), by surgery during admission (yes/no), by known chronic disease/pregnancy, by treatment limitations (Y/N), age and sex. We will present the data in two tables (baseline characteristics of the cohort and vital signs and interventions) with three columns: all patients, critically ill patients, and non-critically ill patients.

Dummy Table 1. Baseline characteristics of the African Critical Illness Outcomes Study (ACIOS) patient cohort

	**All patients (n=?)**	**Patients with critical illness (n=?)**	**Patients without critical illness (n=?)**	**Patients who died (n=?)**	**Patients who survived (n=?)**
**Age**					
Mean (SD)					
Median (IQR)					
**Sex**					
Male	n/N (%)				
Female					
**Known chronic illness or pregnancy**					
Pregnant					
Hypertension					
Diabetes					
Cancer					
COPD/ Asthma					
Heart disease					
HIV/AIDS					
Tuberculosis					
Other					
**Urgency of admission**					
Elective					
Emergency/ acute					
**Main category for admission**					
Non-communicable					
Maternal health					
Trauma					
Infection					
**Ward type**					
Medical					
Surgical					
Maternal					
Other					
**Ward level**					
General ward					
High care ward					
Intensive care unit					

Data are n/N (%). Denominators vary with the completeness of the data. SD standard deviation, IQR interquartile range, HIV human immunodeficiency virus, AIDS acquired immunodeficiency syndrome

Dummy Table 2. Vital signs and essential emergency and critical care treatment interventions of the African Critical Illness Outcomes Study (ACIOS) patient cohort

	**All patients (n=?)**	**Patients with critical illness (n=?)**	**Patients without critical illness (n=?)**	**Patients who died (n=?)**	**Patients who survived (n=?)**
**Position of patient**					
Lying flat on back (<30°)					
Lying on side					
Head-up (30°-60°)					
Sitting (>60°)					
Head-down					
Other					
**Airway patency**					
Normal					
Partial obstruction					
Complete obstruction					
**Conscious level (AVPU)**					
Alert					
Responds to voice					
Responds to pain					
Unresponsive					
**Heart rate**					
Beats per minute					
**Oxygen saturation**					
Percentage					
**Respiratory rate**					
Breathes per minute					
**Blood pressure**					
Systolic blood pressure (mmHg)					
Diastolic blood pressure (mmHg)					
**Current interventions**					
Receiving intravenous fluids					
Receiving oxygen					
Receiving vasopressor or inotrope					
Airway intervention					

Data are n/N (%) or mean (SD). Denominators vary with the completeness of the data. SD standard deviation, IQR interquartile range, AVPU alert verbal pain unconscious, EECC essential emergency and critical care

Sensitivity analyses:
We will conduct a sensitivity analysis for the definition of critical illness whereby we include all of those in the primary definition of critical illness above, *plus* those who do not currently have a severely deranged vital sign but are receiving one of the EECC treatments specified in section 3.6 *or* receiving advanced critical care (e.g. receiving vasopressor/inotrope or treated in an ICU) – as the provision of these treatments may be masking a vital sign derangement.

We will conduct a sensitivity analysis where all patients with 'treatment limitations' (e.g. not for resuscitation) are removed.

We will conduct two sensitivity analyses for missing data required for an assessment of the presence of critical illness (e.g. a vital sign): a 'best case scenario' where missing data are imputed as normal (i.e. critical illness is not present), and a 'worst case scenario' where missing data are imputed as severely deranged (i.e. critical illness is present).

3.5 Statistical analysis plan for Objective 2 *"mortality"*

We will present the number and proportion of critically ill and non-critically patients who die in hospital within the 7 days of data collection. The defined time for the outcomes is from the point of inclusion of the patient into the study to hospital discharge or death, censored at 7-days. Patients discharged alive are not followed-up at home. Patients still in hospital receiving therapy at 7-days will be regarded as "alive" and included in the study.

In the patients who fulfil the criteria for critical illness, a univariate and multivariable logistic regression models will be constructed to determine the relationship between patient factors and mortality. The patient factors which will be entered into the model will include age, sex, category of admission, chronic diseases and pregnancy.

We will use a three-level generalized mixed model, with patients being at the first level, hospital at the second and country at the third level, to account for the expected correlation in outcomes within hospitals and countries. All factors will be entered into the model, unless the number of reported deaths is insufficient to provide 10 events (deaths) per variable. Should the events per variable be <10, then variables with a univariate association of $p<0.05$, and variables with biological plausibility and a low rate of missing data will be prioritized in the model. Collinearity will be assessed using the variance inflation factor. If collinearity is detected, then variables will either be excluded or combined. The model fit will be evaluated.

We will also present the risk of mortality in those with critical illness at the time of census, compared to those without critical illness using logistic regression. A univariate and multivariable logistic regression models will be constructed to determine the relationship between patient factors and mortality. The patient factors which will be entered into the model will include age, sex, category of admission, chronic diseases and pregnancy. We will use a three-level generalized mixed model, with patients being at the first level, hospital at the second and country at the third level, to account for the expected correlation in outcomes within hospitals and countries. All factors will be entered into the model, unless the number of reported deaths is insufficient to provide 10 events (deaths) per variable. Should the events per variable be <10, then variables with a univariate association of $p<0.05$, and variables with biological plausibility and a low rate of missing data will be prioritized in the model. Collinearity will be assessed using the variance inflation factor. If collinearity is detected, then variables will either be excluded or combined. The model fit will be evaluated.

A Kaplan-Meier graph will be constructed of the in-hospital mortality from Day 0 to Day 7 for critically ill and non-critically ill patients. Time will be counted from recruitment to the study until discharge, death or censored. The graph will visualize how mortality risk changes over time. A log-rank test for equality of the survival functions will be performed if the assumptions necessary for using the test hold.[35]
Missing data: patients lost-to-follow-up (missing outcome data) will be included without imputation and reported descriptively. They will not be included in the mortality analysis, but will be included in other analyses.

3.6 Statistical analysis plan for Objective 3 *"receiving EECC"*

We will present the number and proportion of critically ill patients who are receiving EECC. In critically ill patients, we define three categories of 'receiving EECC': no intervention, partial intervention (where some critical ill systems are receiving an EECC intervention, and others are not receiving a EECC intervention), and complete EECC intervention (where all critical ill systems are receiving an EECC intervention).

Patients will be deemed to be receiving EECC if they are:

- critically ill due to the conscious level criterion ***and***:
    - are lying in the lateral position ***or***
    - have an oro-pharyngeal or naso-pharyngeal airway inserted in their pharynx ***or***
    - have an ongoing chin-life or jaw-thrust ***or***
    - have other airway protection.
- critically ill due to a respiratory criterion ***and***:
    - are receiving oxygen.
- critically ill due to a circulatory criterion ***and***:
    - are receiving intravenous fluids ***or***
    - are receiving a vasopressor or inotrope.

We will present a breakdown of data by vital sign derangement, the EECC treatment received, main category of admission, ward type, ward level, urgency of admission, surgery during admission, chronic diseases/pregnancy, treatment limitations (Y/N), age and sex.

Dummy Table 3. Baseline characteristics of critically ill patients in the African Critical Illness Outcomes Study (ACIOS) receiving 'essential emergency and critical care (EECC)'

	**Critically ill patients (n=?)**	**Patients receiving no EECC intervention (n=?)**	**Patients receiving partial EECC intervention (n=?)**	**Patients receiving complete EECC intervention (n=?)**
**Airway patency**				
Normal				
Partial obstruction				
Complete obstruction				
**Conscious level (AVPU)**				
Alert				
Responds to voice				
Responds to pain				
Unresponsive				
**Heart rate**				

Beats per minute				
**Oxygen saturation**				
Percentage				
**Respiratory rate**				
Breathes per minute				
**Blood pressure**				
Systolic blood pressure (mmHg)				
Diastolic blood pressure (mmHg)				
**Current EECC interventions**				
Receiving intravenous fluids				
Receiving oxygen				
Receiving vasopressor or inotrope				
Airway intervention				

Data are n/N (%). Denominators vary with the completeness of the data. SD standard deviation, IQR interquartile range, AVPU alert verbal pain unconscious, EECC essential emergency and critical care

We will conduct a sensitivity analysis whereby all patients receiving one of the EECC treatments as described above and yet the patient still has one or more severely deranged vital sign is regarded as *not* receiving EECC (as an interpretation that the treatment provided is not sufficient).
We will conduct two sensitivity analyses for missing data required for an assessment of the presence of critical illness (e.g. a vital sign): a 'best case scenario' where missing data are imputed as normal (i.e. critical illness is not present), and a 'worst case scenario' where missing data are imputed as severely deranged (i.e. critical illness is present).

3.7 Statistical analysis plan for Objective 4 *"association between the provision of EECC and mortality"*

This objective will be addressed in a dedicated, separate manuscript. A separate SAP will be prepared for this analysis.

3.8 Statistical analysis plan for Objective 5 "availability of resources for EECC"

We will present the resources available for EECC in the hospitals through resource availability scores calculated for each hospital as the number of resources available divided by the total number of EECC resources. Summary measures for the hospitals will be presented. Domain resource availability scores will be calculated for each hospital using the same calculations with just the resources in each domain (equipment, consumables, drugs etc) and summary measures presented.

Missing data: items that are missing data required for an assessment of "resources for EECC" (e.g. a resource in a hospital) will not be included in the analysis.

4. References

1. Kayambankadzanja RK, Schell CO, Gerdin Wärnberg M, et al. Towards definitions of critical illness and critical care using concept analysis. *BMJ open* 2022; **12**(9): e060972.
2. Jenson A, Hansoti B, Rothman R, de Ramirez SS, Lobner K, Wallis L. Reliability and validity of emergency department triage tools in low- and middle-income countries: a systematic review. *European Journal of Emergency Medicine* 2018; **25**(3).
3. Kruisselbrink R, Kwizera A, Crowther M, et al. Modified Early Warning Score (MEWS) Identifies Critical Illness among Ward Patients in a Resource Restricted Setting in Kampala, Uganda: A Prospective Observational Study. *PloS one* 2016; **11**(3): e0151408.
4. Phua J, Faruq MO, Kulkarni AP, et al. Critical Care Bed Capacity in Asian Countries and Regions. *Critical care medicine* 2020.
5. Maharaj R, Raffaele I, Wendon J. Rapid response systems: a systematic review and meta-analysis. *Critical care (London, England)* 2015; **19**(1): 254.
6. Kayambankadzanja RK, Likaka A, Mndolo SK, Chatsika GS, Umar E, Baker T. Emergency and critical care services in Malawi: Findings from a nationwide survey of health facilities. *Malawi Medical Journal* 2020; **32**(1): 259-63.
7. Baker T, Lugazia E, Eriksen J, Mwafongo V, Irestedt L, Konrad D. Emergency and critical care services in Tanzania: a survey of ten hospitals. *BMC health services research* 2013; **13**: 140.
8. Kayambankadzanja RK, Schell CO, Mbingwani I, Mndolo SK, Castegren M, Baker T. Unmet need of essential treatments for critical illness in Malawi. *PloS one* 2021; **16**(9): e0256361.
9. Schell CO, Gerdin Wärnberg M, Hvarfner A, et al. The global need for essential emergency and critical care. *Critical Care* 2018; **22**(1): 284.
10. Schell CO, Khalid K, Wharton-Smith A, et al. Essential Emergency and Critical Care: a consensus among global clinical experts. *BMJ Global Health* 2021; **6**(9): e006585.
11. Crawford AM, Shiferaw AA, Ntambwe P, et al. Global critical care: a call to action. *Critical Care* 2023; **27**(1): 28.
12. Mekontso Dessap A, Richard J-CM, Baker T, Godard A, Carteaux G. Technical Innovation in Critical Care in a World of Constraints: Lessons from the COVID-19 Pandemic. *American journal of respiratory and critical care medicine* 2023.
13. Buowari DY, Owoo C, Gupta L, Schell CO, Baker T. Essential Emergency and Critical Care: A Priority for Health Systems Globally. *Critical care clinics* 2022; **38**(4): 639-56.
14. Rudd KE, Johnson SC, Agesa KM, et al. Global, regional, and national sepsis incidence and mortality, 1990–2017: analysis for the Global Burden of Disease Study. *The Lancet* 2020; **395**(10219): 200-11.
15. Haagsma JA, Graetz N, Bolliger I, et al. The global burden of injury: incidence, mortality, disability-adjusted life years and time trends from the Global Burden of Disease study 2013. *Injury Prevention* 2016; **22**(1): 3.
16. Meara JG, Leather AJ, Hagander L, et al. Global Surgery 2030: evidence and solutions for achieving health, welfare, and economic development. *Lancet* 2015; **386**(9993): 569-624.
17. Schell CO, Wellhagen A, Lipcsey M, et al. The burden of critical illness among adults in a Swedish region—a population-based point-prevalence study. *European Journal of Medical Research* 2023; **28**(1): 322.
18. Mboya EA, Ndumwa HP, Amani DE, et al. Critical illness at the emergency department of a Tanzanian national hospital in a three-year period 2019–2021. *BMC Emergency Medicine* 2023; **23**(1): 86.
19. Murthy S, Wunsch H. Clinical review: International comparisons in critical care - lessons learned. *Critical care (London, England)* 2012; **16**(2): 218.
20. Adhikari NK, Fowler RA, Bhagwanjee S, Rubenfeld GD. Critical care and the global burden of critical illness in adults. *Lancet* 2010; **376**(9749): 1339-46.

21. Kayambankadzanja RK, Schell CO, Namboya F, et al. The Prevalence and Outcomes of Sepsis in Adult Patients in Two Hospitals in Malawi. *The American journal of tropical medicine and hygiene* 2020.
22. Biccard BM, Gopalan PD, Miller M, et al. Patient care and clinical outcomes for patients with COVID-19 infection admitted to African high-care or intensive care units (ACCCOS): a multicentre, prospective, observational cohort study. *The Lancet* 2021; **397**(10288): 1885-94.
23. Ilori IU, Kalu QN. Intensive care admissions and outcome at the University of Calabar Teaching Hospital, Nigeria. *Journal of critical care* 2012; **27**(1): 105 e1-4.
24. Towey RM, Ojara S. Practice of intensive care in rural Africa: an assessment of data from Northern Uganda. *African health sciences* 2008; **8**(1): 61-4.
25. Gombar S, Ahuja V, Jafra A. A retrospective analysis of obstetric patient's outcome in intensive care unit of a tertiary care center. *Journal of anaesthesiology, clinical pharmacology* 2014; **30**(4): 502-7.
26. Subbe CP, Kruger M, Rutherford P, Gemmel L. Validation of a modified Early Warning Score in medical admissions. *QJM : monthly journal of the Association of Physicians* 2001; **94**(10): 521-6.
27. Kause J, Smith G, Prytherch D, Parr M, Flabouris A, Hillman K. A comparison of antecedents to cardiac arrests, deaths and emergency intensive care admissions in Australia and New Zealand, and the United Kingdom--the ACADEMIA study. *Resuscitation* 2004; **62**(3): 275-82.
28. Smith AF, Wood J. Can some in-hospital cardio-respiratory arrests be prevented? A prospective survey. *Resuscitation* 1998; **37**(3): 133-7.
29. Kellett J, Deane B. The Simple Clinical Score predicts mortality for 30 days after admission to an acute medical unit. *QJM-An Int J Med* 2006; **99**(11): 771-81.
30. Baker T, Blixt J, Lugazia E, et al. Single Deranged Physiologic Parameters Are Associated With Mortality in a Low-Income Country. *Critical care medicine* 2015; **43**(10): 2171-9.
31. Baker T, Gerdin M. The clinical usefulness of prognostic prediction models in critical illness. *European journal of internal medicine* 2017.
32. Global patient outcomes after elective surgery: prospective cohort study in 27 low-, middle- and high-income countries. *Br J Anaesth* 2016; **117**(5): 601-9.
33. Pearse RM, Moreno RP, Bauer P, et al. Mortality after surgery in Europe: a 7 day cohort study. *The Lancet* 2012; **380**(9847): 1059-65.
34. Biccard BM, Madiba TE, Kluyts H-L, et al. Perioperative patient outcomes in the African Surgical Outcomes Study: a 7-day prospective observational cohort study. *The Lancet* 2018.
35. Hosmer DW, Lemeshow S, May S. Applied Survival Analysis: Regression Modeling of Time-to-Event Data: Wiley; 2008.

Supplementary Material S7. Participating countries, hospitals and participants recruited

Country	Hospitals	Participants	Proportion of participants
Botswana	1/180	354/19872	1·8%
Burkina Faso	4/180	1226/19872	6·2%
Congo	1/180	82/19872	0·4%
DRC	4/180	42/19872	0·2%
Egypt	2/180	265/19872	1·3%
Ethiopia	8/180	1041/19872	5·2%
Gambia	4/180	266/19872	1·3%
Ghana	18/180	1933/19872	9·7%
Lesotho	2/180	30/19872	0·2%
Libya	28/180	1613/19872	8·1%
Morocco	1/180	246/19872	1·2%
Mozambique	1/180	363/19872	1·8%
Namibia	7/180	677/19872	3·4%
Nigeria	19/180	2286/19872	11·5%
Somalia	3/180	224/19872	1·1%
Somaliland	3/180	74/19872	0·4%
South Africa	37/180	6167/19872	31·0%
Sudan	2/180	39/19872	0·2%
Tanzania	16/180	1338/19872	6·7%
Tunisia	5/180	512/19872	2·6%
Uganda	13/180	1055/19872	5·3%
Zimbabwe	1/180	39/19872	0·2%
**DCP-3 level of hospital***			
Level 1	56/173	2657/19220	13·8%
Level 2	38/173	3846/19220	20·0%
Level 3	79/173	12717/19220	66·2%
**Type of hospital**			
Government	152/173	18472/19477	94·8%
Private	19/173	704/19477	3·6%
Charitable	5/173	301/19477	1·5%
**Number of beds**<br>Median (IQR)			
Total hospital beds	265 (122-519)		
Beds in high care units*	7 (3-16)		
Beds in intensive care units**	7 (2-12)		
Population served (catchment) of the hospital	350 000 (91 715-2 077 500)		

Data are n/N (%). Denominators vary according to completeness of data

* 8/180 hospitals did not report the number of beds in high care units. 36/172 hospitals (20·9%) reported no high care beds.

**7/180 hospitals did not report the number of beds in intensive care units. 42/173 (24·9%) reported no intensive care beds.

***Definitions of hospital levels**

<table>
<tr><td>Level of care</td><td>Alternative terms commonly found in the literature</td></tr>
<tr><td rowspan="5">First-level hospitals: Few specialties—mainly internal medicine, obstetrics and gynaecology, paediatrics, and general surgery; often only one general practice physician or a nonphysician practitioner; limited laboratory services available for general but not specialized pathological analysis; from 50 to 250 beds.</td><td>Primary-level hospital</td></tr>
<tr><td>District hospital</td></tr>
<tr><td>Rural hospital</td></tr>
<tr><td>Community hospital</td></tr>
<tr><td>General hospital</td></tr>
<tr><td rowspan="3">Second-level hospitals: More differentiated by function with as many as 5 to 10 clinical specialties; from 200 to 800 beds.</td><td>Regional hospital</td></tr>
<tr><td>Provincial hospital (or equivalent administrative area such as county)</td></tr>
<tr><td>General hospital</td></tr>
<tr><td rowspan="3">Third-level hospitals: Highly specialized staff and technical equipment—for example, cardiology, intensive care unit, and specialized imaging units; clinical services highly differentiated by function; could have teaching activities; from 300 to 1,500 beds.</td><td>National hospital</td></tr>
<tr><td>Central hospital</td></tr>
<tr><td>Academic or teaching or university hospital</td></tr>
</table>

Supplementary Material S8. Patient position at time of examination

	All patients (n=19872)	Critically ill patients (n=2461)	Patients without critical illness (n=17282)	Patients who died (n=967)	Patients who survived (n=18813)
**Position of patient**					
Lying flat on back (<30°)	6803/19857 (34·3%)	878/2458 (35·7%)	5892/17273 (34·1%)	372/967 (38·5%)	6406/18799 (34·1%)
Lying on side/ recovery position	2848/19857 (14·3%)	328/2458 (13·3%)	2506/17273 (14·5%)	122/967 (12·6%)	2713/18799 (14·4%)
Head-up (30°-60°)	4598/19857 (23·2%)	802/2458 (32·6%)	3746/17273 (21·7%)	361/967 (37·3%)	4207/18799 (22·4%)
Sitting (>60°)	5293/19857 (26·7%)	418/2458 (17·0%)	4846/17273 (28·1%)	101/967 (10·4%)	5172/18799 (27·5%)
Head-down	82/19857 (0·4%)	15/2458 (0·7%)	67/17273 (0·4%)	9/967 (0·9%)	71/18799 (0·4%)
Other	233/19857 (1·2%)	17/2458 (0·7%)	216/17273 (1·3%)	2/967 (0·2%)	230/18799 (1·2%)

Data are n/N (%). Denominators vary with the completeness of the data.

Supplementary Material S9. Data missingness for the generalised linear mixed model

	Missing (n)	Missing (%)	Valid (n)
Mortality	92	0·5%	19780
Age	16	0·1%	19856
Sex	10	0·1%	19862
Pregnant	0	0%	19872
Hypertension	0	0%	19872
Diabetes	0	0%	19872
Cancer	0	0%	19872
COPD/ Asthma	0	0%	19872
Heart disease	0	0%	19872
HIV/AIDS	0	0%	19872
Tuberculosis	0	0%	19872
Other comorbidities	0	0%	19872
Urgency of admission	101	0·5%	19771
Main category for admission	78	0·4%	19794
Patient position	15	0·1%	19857
Airway patency	24	0·1%	19848
Conscious level	26	0·1%	19846
Heart rate	23	0·1%	19849
Oxygen saturation	31	0·2%	19841
Respiratory rate	99	0·5%	19773
Systolic blood pressure	14	0·1%	19858
Diastolic blood pressure	17	0·1%	19855
Critical illness	129	0·6%	19851

Data are n/N (%). Denominators vary with the completeness of the data. COPD chronic obstructive pulmonary disease; HIV/AIDS human immunodeficiency virus/ acquired immunodeficiency syndrome

Supplementary Material S10. Unadjusted and adjusted Cox mixed effects model of factors associated with in hospital mortality

	Unadjusted			Adjusted		
	**Hazard ratio**	**95% confidence interval**	**P-value**	**Hazard ratio**	**95% confidence interval**	**p-value**
**Age per 10 years**	1·33	1·29 – 1·38	**<0·0001**	1·21	1·16 – 1·26	**<0·0001**
**Sex**						
Male	1·30	1·14 – 1·47	**0·0001**	1·11	0·97 – 1·26	0·13
Female	Reference			Reference		
**Known chronic illness or pregnancy***						
Pregnant	0·10	0·05 – 0·18	**<0·0001**	0·70	0·32 – 1·54	0·38
Hypertension	1·61	1·41 – 1·84	**<0·0001**	1·09	0·93 – 1·27	0·29
Diabetes	1·40	1·19 – 1·65	**0·0001**	0·90	0·75 – 1·08	0·25
Cancer	2·45	2·03 – 2·96	**<0·0001**	2·38	1·94 – 2·91	**<0·0001**
COPD/ Asthma	1·52	1·16 – 1·99	**0·0021**	0·79	0·59 – 1·04	0·094
Heart disease	1·84	1·51 – 2·25	**<0·0001**	1·06	0·86 – 1·31	0·59
HIV/AIDS	1·30	1·06 – 1·59	**0·012**	1·27	1·02 – 1·58	**0·032**
Tuberculosis	1·66	1·27 – 2·18	**0·0002**	1·07	0·80 – 1·43	0·65
Other	1·89	1·63 – 2·19	**<0·0001**	1·55	1·33 – 1·80	**<0·0001**
**Urgency of admission**						
Emergency/ acute	3·56	2·78 – 4·56	**<0·0001**	2·62	2·03 – 3·37	**<0·0001**
Elective	Reference			Reference		
**Main category for admission**						
Infection	12·16	7·52 – 19·68	**<0·0001**	3·61	1·91 – 6·80	**0·0001**
Non-communicable disease	10·35	6·46 – 16·59	**<0·0001**	3·09	1·66 – 5·78	**0·0004**
Trauma	3·73	2·23 – 6·22	**<0·0001**	1·75	0·91 – 3·38	0·093
Maternal health	Reference			Reference		

**Critical illness**						
Critically ill	7·31	6·42 – 8·32	**<0·0001**	5·68	4·97 – 6·49	**<0·0001**
Not critically ill	Reference			Reference		

*Reference: absence of risk factor. COPD chronic obstructive pulmonary disease; HIV/AIDS human immunodeficiency virus/acquired immunodeficiency syndrome.

Supplementary Material S11. Unadjusted and adjusted generalized mixed effects model of factors associated with in hospital mortality. Sensitivity analysis: excluding the Nigerian and South African cohorts.

	Unadjusted			Adjusted		
	**Odds ratio**	**95% confidence interval**	**P-value**	**Odds ratio**	**95% confidence interval**	**P-value**
**Age per 10 years**	1·37	1·31 – 1·43	**<0·0001**	1·18	1·12 – 1·25	**<0·0001**
**Sex**						
Male	1·57	1·32 – 1·86	**<0·0001**	1·10	0·91 – 1·34	0·31
Female	Reference			Reference		
**Known chronic illness or pregnancy***						
Pregnant	0·09	0·05 – 0·18	**<0·0001**	0·75	0·31 – 1·77	0·51
Hypertension	1·90	1·58 – 2·28	**<0·0001**	1·23	0·98 – 1·55	0·072
Diabetes	1·66	1·33 – 2·07	**<0·0001**	0·97	0·74 – 1·26	0·80
Cancer	2·72	2·05 – 3·60	**<0·0001**	2·58	1·86 – 3·57	**<0·0001**
COPD/ Asthma	1·66	1·17 – 2·36	**0·0049**	0·81	0·54 – 1·21	0·30
Heart disease	2·34	1·78 – 3·07	**<0·0001**	1·22	0·86 – 1·67	0·21
HIV/AIDS	1·95	1·40 – 2·72	**0·0001**	1·78	1·21 – 2·61	**0·0032**
Tuberculosis	2·28	1·52 – 3·42	**0·0001**	1·30	0·81 – 2·08	0·27
Other	2·25	1·85 – 2·75	**<0·0001**	1·93	1·54 – 2·42	**<0·0001**
**Urgency of admission**						
Emergency/ acute	4·24	3·12 – 5·77	**<0·0001**	2·90	2·10 – 4·01	**<0·0001**
Elective	Reference			Reference		
**Main category for admission**						
Infection	14·48	8·49 – 24·68	**<0·0001**	3·87	1·91 – 7·87	**0·0002**
Non-communicable disease	11·84	7·06 – 19·86	**<0·0001**	3·43	1·71 – 6·85	**0·0005**

Trauma	5·40	3·05 – 9·58	**<0·0001**	2·39	1·14 – 5·00	0·020
Maternal health	Reference			Reference		
**Critical illness**						
Critically ill	10·95	9·13 – 13·14	**<0·0001**	7·94	6·56 – 9·60	**<0·0001**
Not critically ill	Reference			Reference		

*Reference: absence of risk factor. COPD chronic obstructive pulmonary disease; HIV/AIDS human immunodeficiency virus/acquired immunodeficiency syndrome.

Supplementary Material S12. Unadjusted and adjusted Cox mixed effects model of factors associated with in hospital mortality. Sensitivity analysis: excluding the Nigerian and South African cohorts.

	Unadjusted			Adjusted		
	**Hazard ratio**	**95% confidence interval**	**P-value**	**Hazard ratio**	**95% confidence interval**	**P-value**
**Age per 10 years**	1·32	1·26 – 1·37	<0·0001	1·17	1·12 – 1·23	<0·0001
**Sex**						
Male	1·35	1·14 – 1·59	0·0005	1·05	0·88 – 1·24	0·61
Female	Reference			Reference		
**Known chronic illness or pregnancy***						
Pregnant	0·13	0·07 – 0·25	<0·0001	0·79	0·33 – 1·89	0·60
Hypertension	1·73	1·45 – 2·07	<0·0001	1·17	0·95 – 1·44	0·13
Diabetes	1·45	1·17 – 1·80	0·0007	0·90	0·71 – 1·14	0·37
Cancer	2·31	1·77 – 3·00	<0·0001	2·22	1·68 – 2·93	<0·0001
COPD/ Asthma	1·51	1·08 – 2·11	0·017	0·75	0·52 – 1·08	0·12
Heart disease	2·04	1·58 – 2·65	<0·0001	1·21	0·92 – 1·59	0·17
HIV/AIDS	1·72	1·26 – 2·37	0·0008	1·63	1·16 – 2·28	0·0048
Tuberculosis	1·78	1·22 – 2·61	0·0029	1·16	0·77 – 1·74	0·49
Other	1·97	1·63 – 2·39	<0·0001	1·71	1·40 – 2·08	<0·0001
**Urgency of admission**						
Emergency/ acute	3·59	2·64 – 4·87	<0·0001	2·50	1·83 – 3·42	<0·0001
Elective	Reference			Reference		
**Main category for admission**						
Infection	9·77	5·72 – 16·70	<0·0001	3·08	1·51 – 6·26	0·0019
Non-communicable disease	8·43	5·00 – 14·20	<0·0001	2·83	1·41 – 5·67	0·0034

Trauma	3·59	2·02 – 6·38	<0·0001	1·83	0·88 – 3·83	0·11
Maternal health	Reference			Reference		
**Critical illness**						
Critically ill	7·74	6·53 – 9·16	<0·0001	5·90	4·96 – 7·02	<0·0001
Not critically ill	Reference			Reference		

*Reference: absence of risk factor. COPD chronic obstructive pulmonary disease; HIV/AIDS human immunodeficiency virus/ acquired immunodeficiency syndrome

Supplementary Material S13. Model diagnostics and fit for the logistic regression model

Model diagnostics and fit for the logistic regression model was assessed using simulated residuals generated by the DHARMa package in R (Hartig F, 2024).
The multivariable logistic regression model showed acceptable fit, and no significant violations of any model assumptions (Figure S1).

Figure S1. Fit for multivariable logistic regression model

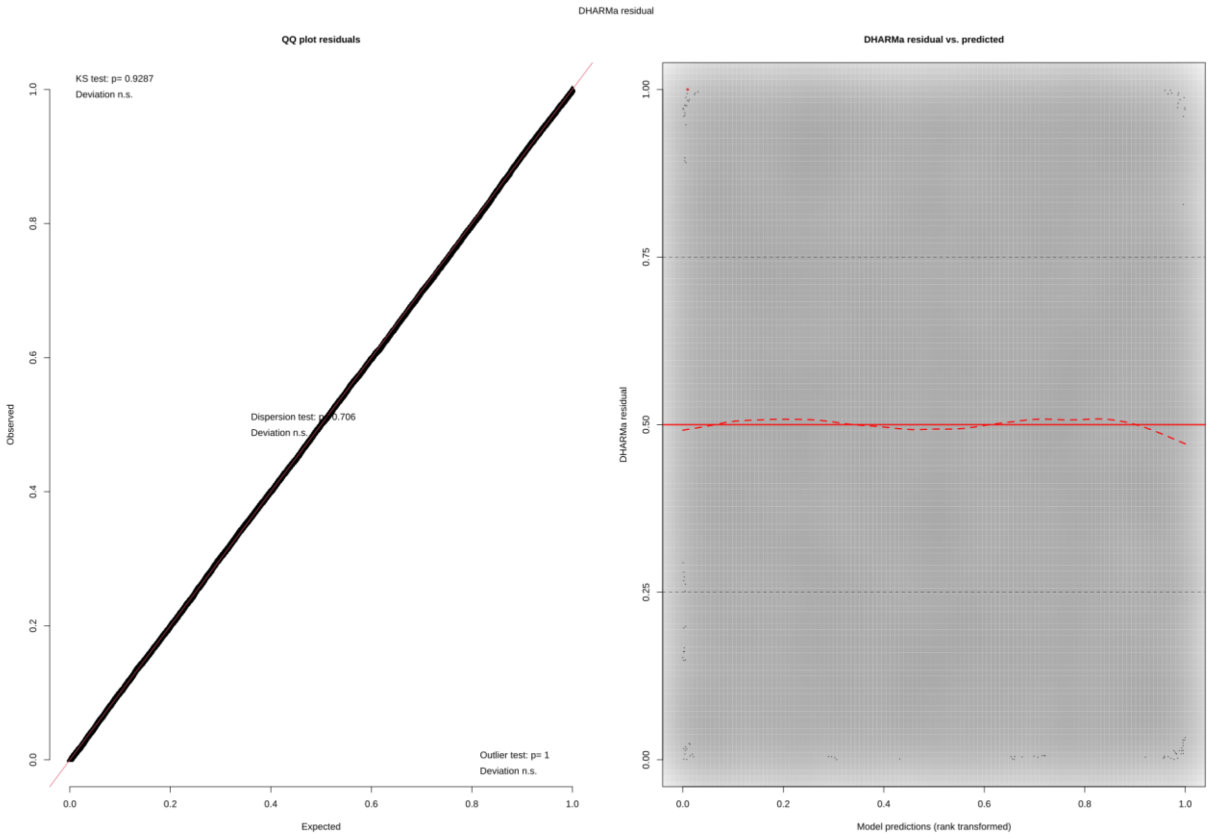

The assumption of linearity for the continuous predictors in the multivariable logistic regression model was assessed using partial-residual plots to detect deviations from linearity.

The multivariable logistic regression models did not show violations of the assumption of linearity. The partial-residual plot for age in the univariable logistic regression model including age as a covariate is shown in Figure S2a, and in the multivariable model including age as a covariate in Figure S2b.

Figure S2a. Partial-residual plot for age in the univariable logistic regression model

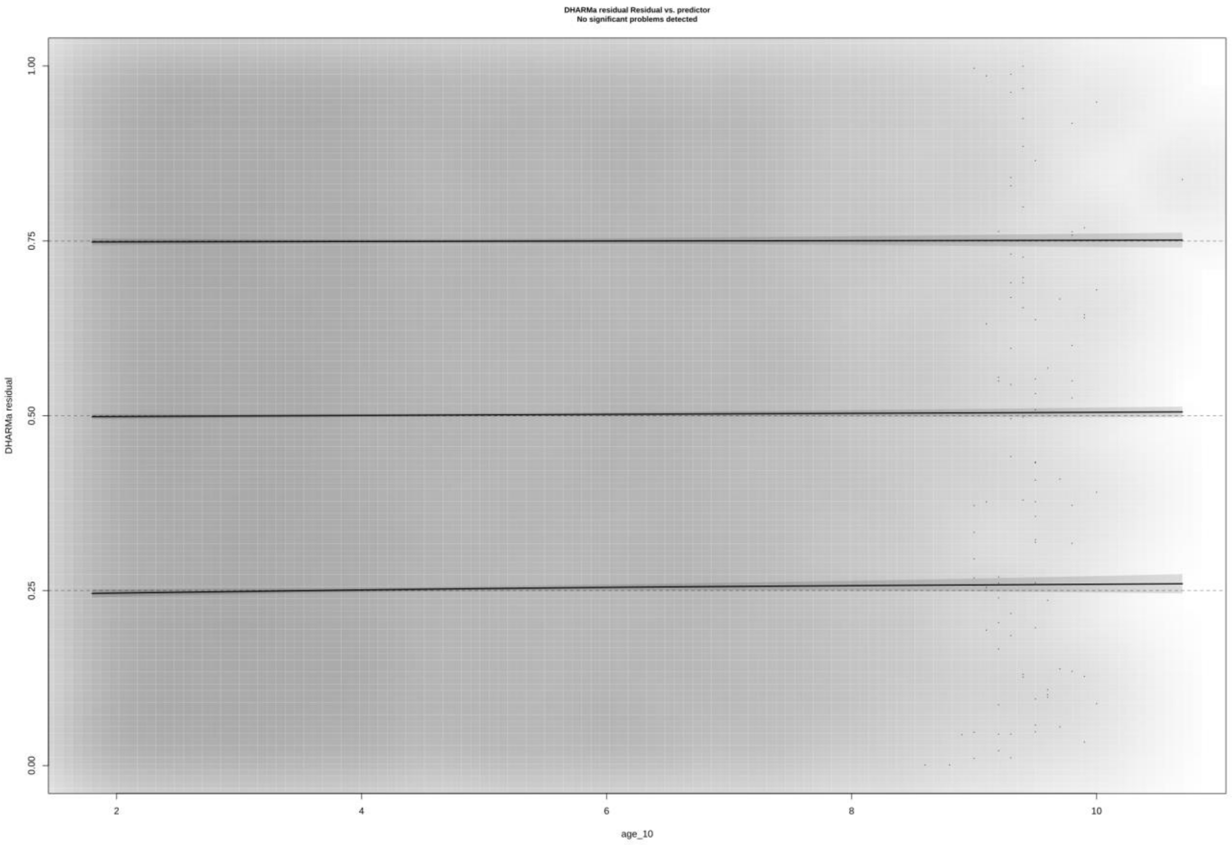

Figure S2b. The partial-residual plot for age in the multivariable logistic regression model including age as a covariate.

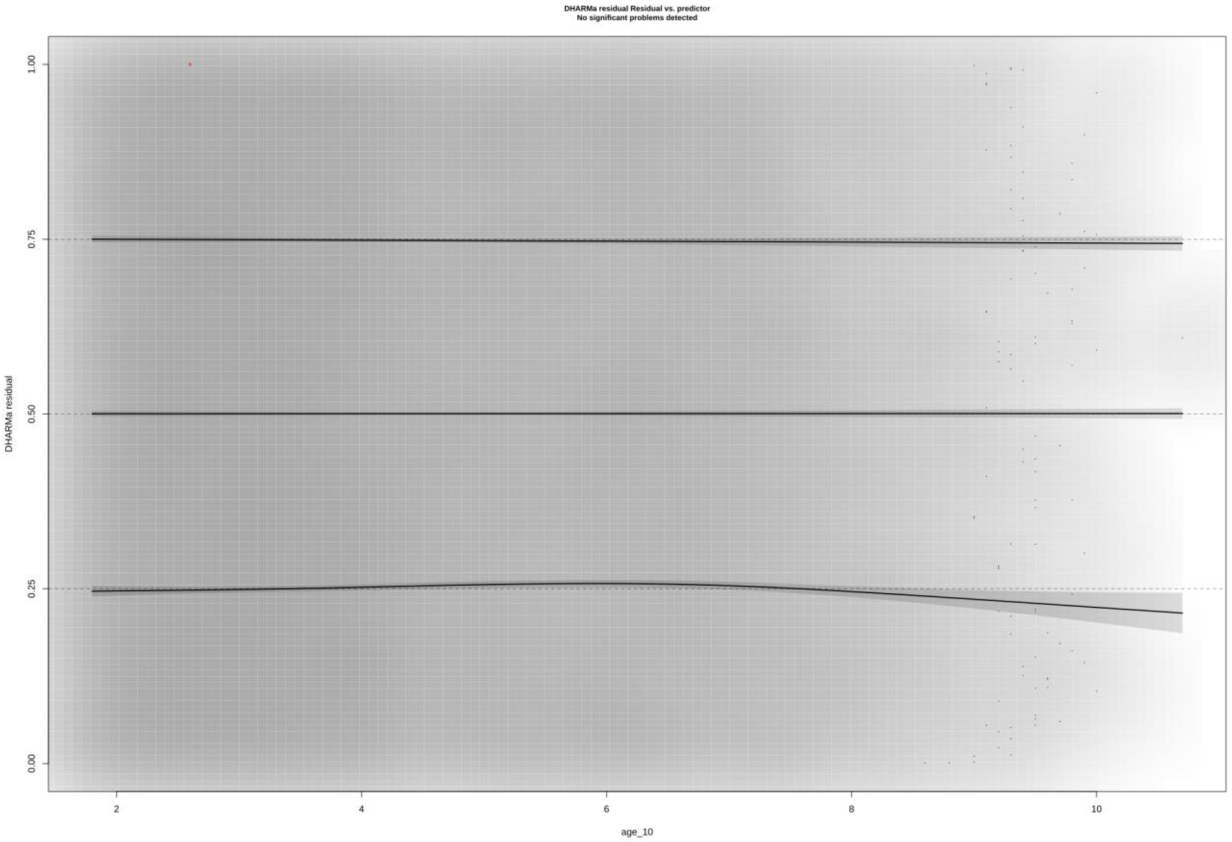

Supplementary Material S14. Resources available for the provision of Essential Emergency and Critical Care (EECC)

Equipment	Always	Sometimes	Never	Do not know
Clock with second hand	99/173 (57·2%)	60/173 (34·7%)	8/173 (4·6%)	6/173 (3·5%)
Pulse oximeter and probe	154/173 (89·0%)	18/173 (10·4%)	1/173 (0·6%)	0
Blood pressure monitoring equipment	160/173 (92·5%)	13/173 (7·5%)	0	0
Blood pressure cuffs of different sizes	116/173 (67·1%)	52/173 (30·1%)	5/173 (2·9%)	0
Light source	127/173 (73·4%)	42/173 (24·3%)	4/173 (2·3%)	0
Thermometer	155/173 (89·6%)	17/173 (9·8%)	1/173 (0·6%)	0
Suction machine	138/173 (79·8%)	33/173 (19·1%)	1/173 (0·6%)	1/173 (0·6%)
Oxygen supply 24h/day	163/173 (94·2%)	9/173 (5·2%)	1/173 (0·6%)	0
Flow meter	141/173 (81·5%)	26/173 (15·0%)	4/173 (2·3%)	2/173 (1·2%)
Leak-free connectors from oxygen source to tubing	133/173 (76·9%)	24/173 (13·9%)	10/173 (5·8%)	6/173 (3·5%)
Bag valve mask	141/173 (81·5%)	26/173 (15·0%)	4/173 (2·3%)	2/173 (1·2%)
Sharps disposal container	165/173 (95·4%)	6/173 (3·5%)	0	2/173 (1·2%)
External heat source	79/172 (45·9%)	63/172 (36·4%)	20/172 (11·6%)	10/172 (5·8%)
**Consumables**				
Soap or hand disinfectant	144/173 (83·3%)	29/173 (16·8%)	0	0
Examination gloves	158/173 (91·3%)	15/173 (8·7%)	0	0
Suction catheters of paediatric and adult sizes	128/173 (74·0%)	42/173 (24·3%)	1/173 (0·6%)	2/173 (1·2%)
Guedel airways of paediatric and adult sizes	130/173 (74·0%)	38/173 (22·0%)	1/173 (0·6%)	4/173 (2·3%)
Pillows	100/173 (57·8%)	56/173 (32·4%)	13/173 (7·5%)	4/173 (2·3%)
Oxygen tubing	153/173 (88·4%)	15/173 (8·7%)	4/173 (2·3%)	1/173 (0·6%)
Oxygen nasal prongs	149/173 (86·1%)	20/173 (11·6%)	4/173 (2·3%)	0
Oxygen face masks of paediatric and adult sizes	133/173 (76·9%)	39/173 (22·5%)	1/173 (0·6%)	0
Oxygen face masks with reservoir bags of paediatric and adult sizes	115/173 (66·5%)	54/173 (31·2%)	3/173 (1·7%)	1/173 (0·6%)
Masks for Bag Valve Mask (resuscitator) – neonatal· paediatric and adult sizes	128/173 (74·0%)	41/173 (23·7%)	3/173 (1·7%)	1/173 (0·6%)
Compression bandages	103/173 (59·5%)	56/173 (32·4%)	7/173 (4·1%)	7/173 (4·1%)
Plasters or tape	153/173 (88·4%)	17/173 (9·8%)	1/173 (0·6%)	2/173 (1·2%)
Gauze	151/173 (87·3%)	22/173 (12·7%)	0	0
Intravenous cannulas of paediatric and adult sizes	166/173 (96·0%)	7/173 (4·1%)	0	0
Intravenous giving sets	161/173 (93·1%)	11/173 (6·4%)	1/173 (0·6%)	0
Skin disinfectant for cannulation	158/173 (91·3%)	15/173 (8·7%)	0	0
Syringes	161/173 (93·1%)	12/173 (6·9%)	0	0
Nutrition	115/173 (66·5%)	48/173 (27·8%)	8/173 (4·6%)	2/173 (1·2%)
Nasogastric tubes	155/173 (89·6%)	17/173 (9·8%)	1/173 (0·6%)	0
Lubricant for nasogastric tube insertion	139/173 (80·4%)	30/173 (17·2%)	4/173 (2·3%)	0
Intramuscular needles	154/173 (89·0%)	15/173 (8·7%)	3/173 (1·7%)	1/173 (0·6%)

Intraosseous cannulas of different sizes	51/173 (29·5%)	51/173 (29·5%)	57/173 (33·0%)	14/173 (8·1%)
Blankets	111/173 (64·2%)	47/173 (27·2%)	12/173 (6·9%)	3/173 (1·7%)
Facemasks for Infection Prevention and Control	145/173 (83·8%)	22/173 (12·7%)	5/173 (2·9%)	1/173 (0·6%)
Aprons or gowns	128/173 (74·0%)	41/173 (23·7%)	4/173 (2·3%)	0
Charts/notes for documentation	157/173 (90·8%)	11/173 (6·4%)	3/173 (1·7%)	2/173 (1·2%)
Pens	130/172 (75·6%)	31/172 (18·0%)	10/172 (5·8%)	1/172 (0·6%)
**Drugs**				
Oral rehydration solution	138/173 (79·8%)	26/173 (15·0%)	7/173 (4·1%)	2/173 (1·2%)
Intravenous crystalloid fluids	159/173 (91·9%)	14/173 (8·1%)	0	0
Intravenous dextrose fluid	154/174 (89·0%)	19/173 (11·0%)	0	0
Oxytocin	147/173 (85·0%)	18/173 (10·4%)	5/173 (2·9%)	3/173 (1·7%)
Adrenaline	157/173 (90·8%)	16/173 (9·3%)	0	0
Appropriate antibiotics	119/173 (68·8%)	52/173 (30·1%)	1/173 (0·6%)	1/173 (0·6%)
Diazepam	142/173 (82·1%)	29/173 (16·8%)	1/173 (0·6%)	1/173 (0·6%)
Magnesium sulphate	141/173 (81·5%)	29/173 (16·8%)	1/173 (0·6%)	2/173 (1·2%)
Paracetamol	155/173 (89·6%)	17/173 (9·8%)	0	1/173 (0·6%)
Local anaesthetic	154/173 (89·0%)	16/173 (9·3%)	0	3/173 (1·7%)
**Human resources**				
Health workers (e.g. nurses) with the ability to identify critical illness 24h/day	148/173 (85·6%)	25/173 (14·5%)	0	0
Health workers with the (e.g. nurses) ability to care for critically ill patients 24hrs/day	143/173 (82·7%)	26/173 (15·0%)	2/173 (1·2%)	2/173 (1·2%)
Senior health worker (e.g. doctor) who can be called to assist with the care of critically ill patients 24hrs/day	141/173 (81·5%)	27/173 (15·6%)	4/173 (2·3%)	1/173 (0·6%)
**Training**				
The health workers are trained in the identification of critical illness	85/173 (49·1%)	79/173 (45·7%)	4/173 (2·3%)	5/173 (2·9%)
The health workers are trained in the care of critically ill patients	84/173 (48·6%)	77/173 (44·5%)	7/173 (4·1%)	5/173 (2·9%)
Routines				
The hospital has well-defined routines for the identification of critical illness	80/173 (46·2%)	77/173 (44·5%)	13/173 (7·5%)	3/173 (1·7%)
The hospital has well-defined routines for managing critically ill patients	92/173 (53·2%)	68/173 (39·1%)	11/173 (6·3%)	2/173 (1·2%)
There is a routine for the provision of EECC without taking into account patients' ability to pay	107/173 (61·9%)	48/173 (27·8%)	13/173 (7·5%)	5/173 (2·9%)
There are routines for who and how to call to seek senior help 24hrs/day, 7 days/week	137/173 (79·2%)	27/173 (15·6%)	7/173 (4·1%)	2/173 (1·2%)

There are routines for integrating EECC with other care including the definitive care of the underlying condition (e.g. use of condition-specific guidelines)	90/173 (52·1%)	60/173 (34·7%)	15/173 (8·7%)	8/173 (4·6%)
**Guidelines**				
There are written guidelines for the identification of critical illness	70/172 (40·7%)	62/172 (36·1%)	34/172 (19·8%)	6/172 (3·5%)
There are written guidelines for the essential care of critically ill patients	71/173 (41·0%)	62/173 (35·8%)	36/173 (20·8%)	4/173 (2·3%)
**Infrastructure**				
Designated triage area (area for the identification of critical illness) in the Out-Patient Department or Emergency Unit (area of the hospital where patients arrive)	143/173 (82·7%)	17/173 (9·8%)	12/173 (6·9%)	1/173 (0·6%)
Running water	135/173 (78·0%)	35/173 (22·0%)	0	0
Designated space for the care of critically ill patients (e.g. a bay· ward· high care unit)	135/173 (78·0%)	29/173 (16·8%)	9/173 (5·2%)	0
Areas for separating and managing patients with a suspected or confirmed contagious disease from those without	116/173 (67·1%)	46/173 (26·6%)	9/173 (5·2%)	2/173 (1·2%)

Supplementary Material S15. Summary of domain resources available for Essential Emergency and Critical Care (EECC)

**EECC resource domains**	**All resources available**
Equipment	53/172 (30·8%)
Consumables	31/172 (18·0%)
Drugs	93/173 (53·8%)
Human resources	121/173 (69·9%)
Training	39/173 (22·5%)
Guidelines	63/173 (36·4%)
Infrastructure	92/173 (53·2%)
All EECC domains	13/172 (7·6%)

EECC Essential Emergency and Critical Care

Supplementary Material S16. Point prevalence of critical illness and mortality in the African Critical Illness Outcomes Study (ACIOS) by the Human Development Index (HDI) of participating countries

**Outcomes**	**n/N (%)**	**95% Confidence interval**
**Prevalence of critical illness**		
Critically ill patients (whole cohort)	2461/19743 (12·5%)	(12·0% - 12·9%)
Critically ill patients (high HDI)	1009/8806 (11·5%)	(10·8% - 12·1%)
Critically ill patients (middle HDI)	447/3940 (11·4%)	(10·4% - 12·3%)
Critically ill patients (low HDI)	1005/6997 (14·4%)	(13·5% - 15·2%)
**7 day in-hospital mortality**		(12·0% - 12·9%)
Mortality (whole cohort)	967/19780 (4·9%)	(4·6% - 5·2%)
Mortality (high HDI)	382/8859 (4·3%)	(3·9% - 4·7%)
Mortality (middle HDI)	180/3939 (4·6%)	(3·9% - 5·2%)
Mortality (low HDI)	405/6982 (5·8%)	(5·3% - 6·4%)

Denominators vary with the completeness of the data. HDI Human Development Index
